# HARmonized Protocol Template to Enhance Reproducibility of hypothesis evaluating real‐world evidence studies on treatment effects: A good practices report of a joint ISPE/ISPOR task force

**DOI:** 10.1002/pds.5507

**Published:** 2022-10-10

**Authors:** Shirley V. Wang, Anton Pottegård, William Crown, Peter Arlett, Darren M. Ashcroft, Eric I. Benchimol, Marc L. Berger, Gracy Crane, Wim Goettsch, Wei Hua, Shaum Kabadi, David M. Kern, Xavier Kurz, Sinead Langan, Takahiro Nonaka, Lucinda Orsini, Susana Perez‐Gutthann, Simone Pinheiro, Nicole Pratt, Sebastian Schneeweiss, Massoud Toussi, Rebecca J. Williams

**Affiliations:** ^1^ Brigham and Women's Hospital Harvard Medical School Boston Massachusetts USA; ^2^ University of Southern Denmark Odense Denmark; ^3^ Brandeis University Waltham Massachusetts USA; ^4^ European Medicines Agency London UK; ^5^ School of Health Sciences University of Manchester Manchester UK; ^6^ Child Health Evaluative Sciences SickKids Research Institute Toronto Ontario Canada; ^7^ ICES Toronto Ontario Canada; ^8^ Department of Paediatrics and Institute of Health Policy, Management and Evaluation, The Hospital for Sick Children University of Toronto Toronto Ontario Canada; ^9^ Independent Consultant New York USA; ^10^ Roche Pharmaceuticals Camberley UK; ^11^ The National Health Care Institute Diemen The Netherlands; ^12^ Utrecht University Utrecht The Netherlands; ^13^ US Food and Drug Administration Silver Springs Maryland USA; ^14^ Sanofi‐Aventis US LLC North Potomac Maryland USA; ^15^ Janssen Research & Development, LLC Philadelphia Pennsylvania USA; ^16^ London School of Tropical Hygiene London UK; ^17^ Pharmaceutical and Medical Devices Agency Japan; ^18^ COMPASS Pathways Ltd Skillman UK; ^19^ RTI Health Solutions Barcelona Spain; ^20^ Quality Use of Medicines and Pharmacy Research Centre, Clinical and Health Sciences University of South Australia South Australia Australia; ^21^ IQVIA France; ^22^ Bethesda Maryland USA

**Keywords:** protocol, real world evidence, reproducibility, transparency

## Abstract

**Problem:**

Ambiguity in communication of key study parameters limits the utility of real‐world evidence (RWE) studies in healthcare decision‐making. Clear communication about data provenance, design, analysis, and implementation is needed. This would facilitate reproducibility, replication in independent data, and assessment of potential sources of bias.

**What We Did:**

The International Society for Pharmacoepidemiology (ISPE) and ISPOR–The Professional Society for Health Economics and Outcomes Research (ISPOR) convened a joint task force, including representation from key international stakeholders, to create a harmonized protocol template for RWE studies that evaluate a treatment effect and are intended to inform decision‐making. The template builds on existing efforts to improve transparency and incorporates recent insights regarding the level of detail needed to enable RWE study reproducibility. The overarching principle was to reach for sufficient clarity regarding data, design, analysis, and implementation to achieve 3 main goals. One, to help investigators thoroughly consider, then document their choices and rationale for key study parameters that define the causal question (e.g., target estimand), two, to facilitate decision‐making by enabling reviewers to readily assess potential for biases related to these choices, and three, to facilitate reproducibility.

**Strategies to Disseminate and Facilitate Use:**

Recognizing that the impact of this harmonized template relies on uptake, we have outlined a plan to introduce and pilot the template with key international stakeholders over the next 2 years.

**Conclusion:**

The HARmonized Protocol Template to Enhance Reproducibility (HARPER) helps to create a shared understanding of intended scientific decisions through a common text, tabular and visual structure. The template provides a set of core recommendations for clear and reproducible RWE study protocols and is intended to be used as a backbone throughout the research process from developing a valid study protocol, to registration, through implementation and reporting on those implementation decisions.

**Key Points**

- Ambiguity in communication of key study parameters limits the utility of real‐world evidence studies in healthcare decision‐making.
- The International Society for Pharmacoepidemiology and ISPOR–The Professional Society for Health Economics and Outcomes Research convened a joint task force, including representation from key international stakeholders, to create a harmonized protocol template for RWE studies that evaluate a treatment effect and are intended to inform decision‐making.
- The HARmonized Protocol Template to Enhance Reproducibility helps to create a shared understanding of intended scientific decisions through a common text, tabular and visual structure.

**Plain language summary**

Ambiguity in communication of key study parameters limits the utility of real‐world evidence (RWE) studies in healthcare decision‐making. The International Society for Pharmacoepidemiology and ISPOR–The Professional Society for Health Economics and Outcomes Research convened a joint task force to create a harmonized protocol template for RWE studies. The template builds on existing efforts to improve transparency and incorporates recent insights regarding the level of detail needed to enable study reproducibility. The overarching principle was to reach for sufficient clarity to achieve three main goals. One, to help investigators thoroughly consider, then document their choices and rationale for key study parameters that define the causal question, two, to facilitate decision‐making by enabling reviewers to readily assess potential for biases related to these choices, and three, to facilitate reproducibility. The HARmonized Protocol Template to Enhance Reproducibility helps to create a shared understanding of intended scientific decisions through a common text, tabular and visual structure. The template provides a set of core recommendations for clear and reproducible RWE study protocols and is intended to be used as a backbone throughout the research process from developing a valid study protocol, to registration, through implementation and reporting on those implementation decisions.

## 1 BACKGROUND

Regulatory agencies, health technology assessors, and payers are increasingly interested in studies that make use of real‐world data (RWD) to inform regulatory and other policy or clinical decision‐making.^1^, ^2^, ^3^, ^4^, ^5^ While real‐world evidence (RWE) studies using rigorous methods applied to fit‐for‐purpose RWD can provide critical, timely insights into the safety and effectiveness^6^, ^7^, ^8^ of drugs, devices, and vaccines; high‐profile cases of studies conducted with biased methods^9^, ^10^, ^11^, ^12^ or inadequate reporting on unsuitable data^13^, ^14^, ^15^ have raised concerns over the credibility of RWE studies. These concerns have led to increasing calls from the research community and decision‐makers for more transparency on the design and conduct of studies using RWD.^16^, ^17^, ^18^

Some initiatives are already in place. As an example, the European Medicines Agency (EMA) has, for over a decade, required or recommended registration of a study protocol using a template for observational post‐authorization safety studies (PASS) conducted by marketing authorization holders.^19^, ^20^ However, a large scale evaluation of the reproducibility of 150 studies highlighted that there remains a great deal of variability in transparency about critical details of RWE study implementation,^21^ and recently, the EMA endorsed a strategy for moving toward greater standardization and structure in protocols.^22^

Clear communication within multi‐disciplinary study teams and between investigators, decision‐makers and other stakeholders is necessary to increase confidence in RWE study design, conduct, and results. The rapid development of fragmented recommendations^23^ has highlighted the need for an internationally agreed upon set of core expectations regarding best practices for developing and communicating about study design, analysis, and implementation via transparent, comprehensive, and rigorous RWE study protocols. A joint task force between the International Society for Pharmacoepidemiology (ISPE) and the ISPOR–The Professional Society for Health Economics and Outcomes Research (ISPOR) was convened to meet this need by developing a harmonized protocol template for RWE studies that make secondary use of RWD, evaluate a hypothesis and are intended to inform healthcare decision‐making. The task force was comprised of core committee members from both professional societies, and included international stakeholder groups including regulatory agencies, health technology assessment (HTA) organizations, industry, and academia.

The task force was primarily focused on protocols for post‐marketing studies that deal with questions of causal inference using RWD because of their importance to decision making and the complexity of design and analysis when addressing causal questions. Examples of such studies include comparative effectiveness or safety studies associated with clinical interventions, studies of the effect of policy interventions such as benefit designs or healthcare delivery models, health care expenditures or value associated with different treatments, and so forth. While it is also important to develop protocols for non‐causal inference studies using RWD, that was not the focus of the protocol harmonization effort.

The task force met monthly from July 2021 to January 2022 to develop the harmonized template. The process of developing the harmonized template included both evaluation of external validity (through comparison of existing protocol templates or guidance developed by international multi‐stakeholder groups to ensure compatibility with agreed upon scientific principles) and internal validity (through testing and development of example use cases with different designs and data sources by five sub‐teams). The final deliverable was a standard template with embedded instruction which harmonized across existing guidance and templates and example protocols for a variety of use cases to illustrate how to use the template.

### 1.1 Identification and comparison of protocol templates

**FIGURE 1:**
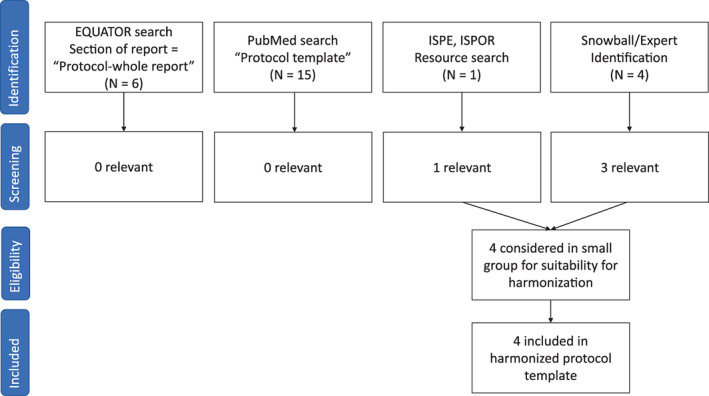
PRISMA diagram

Existing protocol templates for RWE studies were identified based on templates known to the core committee of the joint task force, coupled with a search for relevant protocol templates in PubMed and the EQUATOR network (Enhancing the QUAlity and Transparency Of health Research) (Figure 1, Appendix S1). Additionally, an extended reviewer group composed of volunteers from ISPE and ISPOR were asked to review the list of identified protocol templates and to supplement the list with other templates that they were aware of. Protocol templates that were not relevant for RWE studies that make secondary use of healthcare data or were not developed by international multi‐stakeholder groups were excluded. This resulted in four eligible protocol templates; the European Medicines Agency (EMA) Heads of Medicines Agency Guideline on Good Pharmacovigilance Practices (GVP) Module VIII ‐ post‐authorizations safety studies (PASS) template,^20^ ISPE's guidelines for good pharmacoepidemiology practice (GPP) section on protocol development,^24^ National Evaluation System for health Technology (NEST) protocol guidance,^25^ and the Structured Template and Reporting Tool for Real World Evidence (STaRT‐RWE).^26^

Section headings of the identified protocol templates were compared and mapped to each other, using the oldest guideline (EMA‐GVP Module VIII‐PASS) as the starting point (Table 1). The committee observed that at a conceptual level, the major elements of study design and analysis were already largely agreed upon and included in each of the templates. However, the templates differed on the depth and detail of guidance within each section as well as the sequencing of elements within the template. Three of the protocol templates offered a few sentences or paragraphs of guidance on what sort of information to include within each section (EMA‐GVP Module VIII‐PASS, ISPE‐GPP, and NEST), allowing the user flexibility in free‐text entries under the section header. The most recently published template (STaRT‐RWE) used structured tables to guide the user on where, what and how to specify study implementation details. The STaRT‐RWE template tables and figure also had a strong focus on clearly delineating time zero for entry into the study population and orienting baseline and follow up windows around that primary temporal anchor. A high‐level summary of other differences in format and depth of detail requested by each template is provided in supplemental appendices (Appendix S2).

**TABLE 1:** Comparison of four protocol templates for real‐world evidence studies developed by multi‐stakeholder, international organizations.

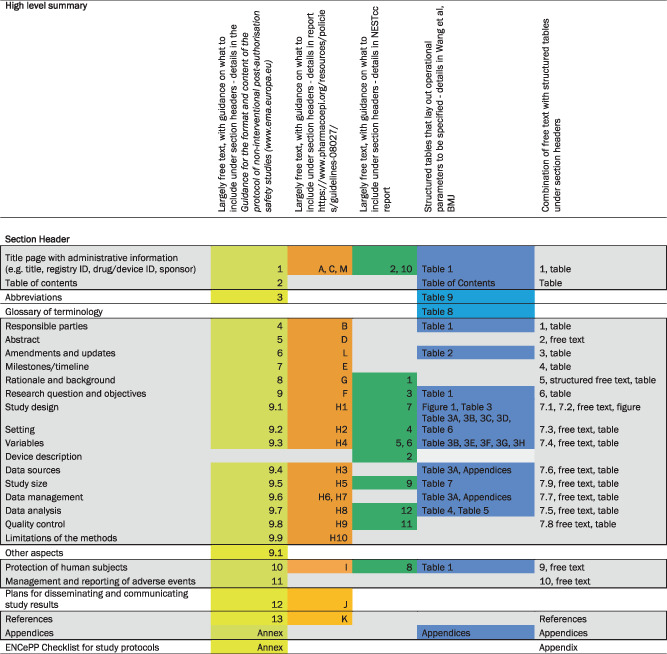

*Note*: Shaded gray area within bold black lines reflects core protocol components.

### 1.2 Creation of HARmonized Protocol Template to Enhance Reproducibility

In order to create a harmonized template, the core committee of the joint task force discussed each section header in the mapped table of protocol templates. Again, starting with the EMA‐GVP Module VIII‐PASS template, the committee evaluated the different sections, guidance and/or structure of more recently developed protocol templates under the same section header, jointly deciding how to incorporate these updates into the harmonized protocol template. The committee then categorized the sections as core elements required for any RWE study protocol and non‐core elements that may provide important context, administrative and other information (Table 1). Core elements of the protocol were defined as sections that were either considered key for the purposes of reproducibility and validity assessment or were common elements that were found in multiple protocol templates and were important to consider core for administrative or other reasons.

After populating an initial mock‐up template, the core committee discussed and concluded that a combination of free‐text and structured tables would increase the rigor and clarity in communication about study implementation decisions. Therefore, the structure of the harmonized protocol template largely follows the headers of the EMA GVP Module VIII‐PASS template, with free‐text and structured free‐text prompts (in the form of helper text) aligned with the ISPE and NEST protocol guidance. These free text sections are where context and rationale for scientific decisions are entered. In sections about study methods, free‐text is accompanied by structured tables from the STaRT‐RWE template. The tables are where details of operational study implementation are specified. The free‐text and structured tables are supplemented by detailed clinical code lists, algorithms, and descriptions of data linkage or data transformation in appendices.

### 1.3 Piloting the usability of HARPER

To pilot the usability of the draft harmonized template, the core committee formed five subgroups. These subgroups had the task of populating the draft harmonized protocol template for a variety of use cases that involved different study designs, data sources, and types of data elements. Four of these use cases were based on published effectiveness and safety studies and one was for a study that was in the planning/design phase (Table 2). The members of each subgroup worked together to populate the initial version and relayed any issues to the core committee at large for discussion. The harmonized protocol template was revised to improve usability following this group exercise. These revisions included expanding the set of sections that were considered core, re‐labeling of some structured prompts, and the addition of more helper text to guide investigators in use of the template. The abbreviated protocols for each use case was transferred onto the final version of the template to provide guidance and examples for future users (Appendix S3).

**TABLE 2:** Example use cases

Use case	Question	What is unique
Empagliflozin versus DPP4i on 3P‐MACE	Effectiveness	Existing protocol was reconstructed with more details added to match expectations in harmonized template (cohort study)
New cancer therapy compared to standard of care	Effectiveness	Harmonized template used for new protocol in development (cohort study)
Pioglitazone and risk of bladder cancer	Safety	Nested case–control design
Topiramate and oral clefts	Safety	Pregnancy cohort study with more complexity in design parameters
Palivizumab and RSV	Effectiveness	Self‐controlled design

### 1.4 Core sections of HARPER

Following the title page, abstract, and a table for amendments and updates, there are nine sections for the harmonized template, each of which includes structured free text, a structured table, or a figure (Table 3, Appendix S4). The majority of the protocol is focused on the critically important research methods (Section 1.4.7), where there are numerous subsections organized in the same way. A free‐text section to lay out context and rationale for scientific choices is coupled with a table or figure to provide details on operational definitions.

**TABLE 3:** HARPER table of contents

1. Title Page
2. Abstract
3. Amendments and updates
4. Timeline
Table 1 Milestones and Timeline
5. Rationale and background
6. Research question and objectives
Table 2 Primary and secondary research questions and objective
7. Research methods
7.1. Study design
7.2. Study design diagram
7.3. Setting
7.3.1 Context and rationale for definition of time 0 (and other primary time anchors) for entry to the study population
Table 3 Operational Definition of Time 0 (index date) and other primary time anchors
7.3.2 Context and rationale for study inclusion criteria:
Table 4. Operational Definitions of Inclusion Criteria
7.3.3 Context and rationale for study exclusion criteria
Table 5. Operational Definitions of Exclusion Criteria
7.4. Variables
7.4.1 Context and rationale for exposure(s) of interest
Table 6. Operational Definitions of Exposure
7.4.2 Context and rationale for outcome(s) of interest
Table 7. Operational Definitions of Outcome
7.4.3 Context and rationale for follow up
Table 8. Operational Definitions of Follow Up
7.4.4 Context and rationale for covariates (confounding variables and effect modifiers, e.g. risk factors, comorbidities, comedications)
Table 9. Operational Definitions of Covariates
7.5. Data analysis
7.5.1 Context and rationale for analysis plan
Table 10. Primary, secondary, and subgroup analysis specification
Table 11. Sensitivity analyses – rationale, strengths and limitations
7.6. Data sources
7.6.1 Context and rationale for data sources
Table 12. Metadata about data sources and software
7.7. Data management
7.8. Quality control
7.9. Study size and feasibility
Table 13. Power and sample size
8. Limitation of the methods
9. Protection of human subjects
10. Reporting of adverse events
11. References
12. Appendices

#### 1.4.1 Title page

The title page includes a table for administrative details, such as the title of the protocol, brief objectives, a protocol version date, names of investigators and sponsor, study registration, and potential conflicts of interest.

#### 1.4.2 Abstract

The abstract is a free text section that includes a description of the background, research question and objectives, study design, and data sources.

#### 1.4.3 Amendments and updates

The protocol is intended to be a living document over the lifecycle of the study. Therefore, it is important to keep track of what changed and why the changes were made. This table documents what is changed, when it is changed, and why. For example, over the process of developing and implementing a protocol, investigators could start with an initial version of inclusion–exclusion criteria for doing an initial set of feasibility counts (looking at outcome counts that are not stratified by exposure), in version 2 using revised algorithms to generate a second set of feasibility counts to evaluate whether there is enough power and assess diagnostics such as propensity score overlap and balance, and in version 3 using finalized algorithms to create the analytic cohort.

#### 1.4.4 Milestones

This section includes a table to outline the anticipated timeline for study milestones.

#### 1.4.5 Rationale and background

This section includes structured free‐text prompts to encourage inclusion of important key contextual information. For example, a paragraph about what is known about the condition and the exposures being investigated, knowledge gaps, and the expected contribution from the study described in the protocol.

#### 1.4.6 Research question and objectives

The prompts ask the user to summarize PICOT information – that is the population, intervention/exposure, comparator, outcome, and time horizon for the study (when follow up begins and ends) ‐ as well as the main measure of effect. The text prompts closely align with the information needed to compare a RWE study design to a theoretical trial designed to address the same question (e.g., a target trial^27^). This section includes structured free‐text prompts to specify the primary and secondary objectives, as well as the hypotheses being tested for each.

#### 1.4.7 Research methods

##### Study design and study design diagram

This section includes structured free‐text prompts that ask the user to name the design and rationale for the choice of design. The structured free‐text prompts are coupled with a design diagram, which the joint task force members agreed would be a critically important part of the harmonized protocol template because this figure provides a concise visual abstract to summarize the design of the study. We recommend a recently developed graphical framework for depicting study design for studies conducted with RWD,^28^ but other visualizations can be used as appropriate. Conceptual models or directed acyclic graphs may be included as well.

##### Setting and variables

These sections include a free‐text component to discuss rationale and context for choices relating to setting (selection of time zero [1], inclusion [2], exclusion criteria [3]) and variables (exposure [a], outcome [b], follow up [c], and covariates [d]). Each free‐text component is followed by a structured table which prompts users to specify what is measured, the timing of measurement, the care setting (e.g., inpatient, outpatient, emergency department), type of codes that are used to define the measure (for example, drug, diagnosis, procedure or lab codes), as well as the sources for any algorithms used to derive study measures, for example, defining exposures, outcomes or covariates (whether that be from a publication or clinician review). For algorithms based on diagnosis codes, there is a section to define whether codes are required to be in the primary position (suggesting that the diagnosis is the main reason for the encounter). The clinical codes used to define each measure are specified later, in structured, machine‐readable appendices as part of Section 1.9. Each table also includes fields to indicate whether the study parameter was pre‐specified, whether it was varied for sensitivity, and the source of the algorithm to define that measure. Examples of how to populate the template for algorithms that are not based on clinical codes are available in the examples provided in supplemental appendices (Appendix S3).

In the outcome table (b), there is an additional field to specify the performance or validation of outcome algorithms, as well as a field to indicate which are the primary and which are the secondary outcomes. For the covariate table (d), there are also fields to specify things like how the variables are modeled (for example, as continuous, or categorical variables).

The structure of the follow up table (c) is different. The table uses structured fields to define when follow up begins relative to cohort entry and how it ends. The prompts help the investigator to consider each option and also makes it clear for the reviewer what is and is not used to end follow up. The table has fields to specify a variety of conditions that could end follow up such as death, disenrollment, a fixed calendar date, or end of exposure, with prompts to provide details. For example, on how duration of therapy is defined, it can be helpful to specify decisions regarding how to handle early refills or conversely short gaps in between dispensations.

##### Data analysis

This section is where the primary, secondary and subgroup analyses are specified. The context and rationale are discussed in the free‐text component. The structured table includes fields for the hypothesis being tested, software packages, the specific models that are fit, the type of confounding adjustment, with prompts for specification of key parameters such as matching ratio and caliper for matched analyses, formulas for weights, trimming and truncation rules. Also reported in this table are fields to specify how missing data are handled in the analysis and subgroup analyses. For example, the investigator might choose to exclude patients with missing or unknown age and use multiple imputation for missing laboratory values.

There is also a structured table for detailing what sensitivity analyses are conducted and the rationale for conducting them (in other words, stating what might be learned from the sensitivity analysis). This rationale is especially important to help end users make sense of and interpret the results, particularly in a discipline where it is easy to run many sensitivity analyses.

##### Data sources

There is a free text component followed by a structured table for specifying data sources. The free text includes structured prompts to state the reasons for selecting the data, strengths and limitations of the data source(s) and information about data source provenance/curation. As shown in the examples, users may refer to detailed materials developed by data providers. This section can also include a detailed evaluation of the fitness‐for‐purpose of data source options, as outlined in the SPIFD^29^ framework for identifying fit‐for‐purpose data or the EUnetHTA REQUEST^30^ tool. The structured table outlines details such as the data source name, the data version, extraction date, sampling criteria (if relevant), data linkage,^31^, ^32^ or conversion to a common data model^33^, ^34^, ^35^ are summarized in this table with more detail and data dictionaries in appendices as needed.

##### Data management

This section includes only a free‐text component where the investigator can specify procedures for securely receiving, quality checking, storing, backing up and preparing data.

##### Quality control

This section includes only a free‐text component where the investigator describes steps for quality assurance or quality check procedures, such as double programming or assessment of the reliability of the data (e.g., missing or miscoded data).

##### Study size and feasibility

This is a free text section where the appropriate precision, power and study size calculations are delineated to address the research questions, with description of the assumptions being made and sources that were used to make the assumptions. A table may be used to provide the selected parameters used in the power/sample size calculation if relevant.

#### 1.4.8 Limitation of the methods

This section is free‐text and provides space for the investigators to summarize the anticipated limitations of the methods and data described in Section 1.4.4.

#### 1.4.9 Protection of human subjects

This free‐text section is intended for the investigators to describe patient privacy protections and the plan to maintain data confidentiality or prevent re‐identification. For example, investigators may report how the data were anonymized or pseudo‐anonymized, whether small cell sizes were suppressed (if the data holder requires), and/or whether the study protocol underwent ethics review. For many studies using RWD, the latter may not be applicable. If the study is considered exempt by the relevant ethics board this should be stated with the reason it is considered exempt.

#### 1.4.10 Reporting of adverse events

This free‐text section is for investigators to state the plan to report adverse events. This reporting is mandated for certain types of post‐authorization studies.^36^ If it is not applicable, that can be stated here.

#### References

1.4.11

This section is for providing a bibliography for cited work.

#### 1.4.12 Appendices

The structured, human readable tables in the harmonized template are intended to be accompanied by appendices that list out the clinical code algorithms in a way that can be directly read in by programming code to facilitate creation of study variables. An example is provided in supplemental Appendix S3 Example 1. Appendices to detail other things, like decisions made when converting source data to a common data model or doing data linkage may also be relevant, depending on the study. Some appendices (e.g., specifying clinical code algorithms used for covariates), may not be developed until later versions of the protocol as the study progresses. Likewise, over the course of the conduct of the study, algorithms included in the appendices may be amended, with the changes documented in the amendments table. Some investigators may use code algorithms that they consider proprietary. If that is the case, this should be so noted in the protocol, thus allowing the reviewer to weigh the potential impact of not having this information on their ability to evaluate the validity or relevance of the study results.

## 2 DISCUSSION

A joint task force between ISPE and ISPOR, including representation from key international stakeholders was formed to create a harmonized protocol template for RWE studies that evaluate a treatment effect and are intended to inform decision‐making. HARmonized Protocol Template to Enhance Reproducibility (HARPER) builds on existing efforts, providing clarity, structure, and a common denominator regarding the level of operational detail, context, and rationale necessary in a protocol to produce a transparent, reproducible study and to support assessment of fitness‐for‐purpose. The overarching principle was to reach for sufficient clarity in the protocol regarding data, design, analysis, and implementation over the lifecycle of a study to achieve three main goals. One, to help investigators thoroughly consider, then document their decisions and rationale regarding key study parameters that define the causal question (e.g., target estimand^37^). In this way, the template could help investigators to think more carefully about their choices and be used to help train a future generation on best practices. The second goal was to facilitate decision‐making by enabling reviewers to readily assess potential for biases related to the clearly communicated investigator choices and rationale. The third goal was to facilitate reproducibility of results.

While the primary focus was on hypothesis evaluating RWE studies, HARPER can also be used as the basis of protocols for descriptive, utilization, predictive or other types of RWE studies. However, there may be some variation regarding which sections are considered core versus optional for different stakeholders (e.g., regulatory, HTA, academic, etc.).

### 2.1 Parallel workstreams, relationships to checklists/bias assessment tools for RWE

In addition to issues of transparency, many professional associations, regulatory bodies, and health technology assessment agencies have issued best practice guidelines and checklists for the analysis of RWD. ISPOR,^38^, ^39^, ^40^, ^41^, ^42^ ISPE,^24^, ^43^, ^44^ the FDA,^1^, ^45^, ^46^ the EMA,^17^, ^47^ the European Network of Centres for Pharmacoepidemiology and Pharmacovigilance (ENCePP®),^5^, ^48^, ^49^, ^50^ and the European Network for Health Technology Assessment (EUnetHTA)^51^ and the Japanese Pharmaceuticals and Medical Device Agency (PMDA)^52^ have all published guidance documents on good practice. Widely used checklists for the reporting of observational studies include RECORD‐PE,^53^ STROBE^54^ and CHEERS.^55^ Several bias assessment tools^56^, ^57^, ^58^ have been developed as well. However, these rely on provision of sufficient details on study methods to enable effective assessment of what was planned, what was done, and how these methods relate to what was found (e.g., validity). The harmonized protocol template aims to help investigators communicate clearly and effectively with reviewers and is consistent with current recommendations and strategies from key stakeholders.

In addition to improving transparency about hypothesis evaluating studies conducted with RWD, it is our hope that use of a harmonized protocol template will guide investigators in thinking about issues of study design, epidemiological and statistical methods, thereby reducing avoidable mistakes. Indeed, issues with study design may be even more important than confounding due to lack of randomization in explaining inconsistencies between RWE and RCT results.^59^, ^60^ Relatedly, assessing whether the data used for the study are fit‐for‐purpose is critical for considering issues of bias introduced by measurement error and inadequate control for confounders. Data that are fit‐for‐purpose for some study questions and designs may not be fit for others (e.g., an outpatient claims based data source is not fit for evaluating a study on the effectiveness of alternative inpatient therapies). The structured, harmonized protocol template outlines necessary details on the study question, design, and data to enable assessment of their collective fitness‐for‐purpose.

### 2.2 Limitations

There are several limitations to HARPER. First, there is a trade‐off between setting common standards for communicating about study design, analysis, and implementation versus full freedom to describe these in whatever fashion the investigator chooses. For example, the structured tables of the template may be challenging for studies that use complex, emerging designs. However, there is always the option of using the free‐text sections to provide context. Relatedly, the sequencing of the sections in HARPER cannot align with all existing templates and processes from potential users across different subdisciplines. If users find that an alternative sequence better fits their needs (e.g., data sections before design, tables at the end instead of integrated in each section), they can re‐order as needed. Second, our focus was on creating a harmonized protocol template to document and enable clear communication of scientific decisions for studies that make secondary use of real‐world data for causal inference. This does not cover every aspect of transparency over the lifecycle of a research study, which may involve sharing of protocol, code, data, as well as results. Third, the guidance documents and templates identified and used in this harmonization effort may not include all relevant guidance that have been developed by different organizations around the world, however, among the major guidance documents that were identified, we have observed a great deal of concordance in the main elements. Fourth, as described earlier, this effort to change the status quo will rely on successful integration of the template into existing processes and guidance by key stakeholders. Thus, we have outlined a plan to pilot use of the template with multiple international regulatory, HTA, and payer stakeholders. Finally, real‐world data analytics is a rapidly evolving field and while the template is flexible, it may need iterative revision. Therefore, we plan to have the harmonized template reviewed and updated as needed through a standing review process that is part of ISPE's policy for endorsed papers or products.

### 2.3 Strategies to disseminate and facilitate use

In addition to this publication introducing HARPER, presentation at and endorsement from prominent research societies, we are engaging with international, inter‐disciplinary stakeholders to lay the foundation toward routine use of the template for development of clearly specified protocols for RWD studies intended to inform decision‐making. Once published, HARPER will be freely available for anyone to download and use. However, recognizing that the impact of HARPER relies on uptake, we have outlined a strategy to introduce and pilot the template with numerous key stakeholders over the next 2 years. We will be presenting the template directly to international regulatory agencies and HTA groups and are laying the groundwork to pilot test the template with ongoing demonstration projects that are evaluating or guiding the use of RWE to support decision‐making.

Furthermore, we intend to engage with study registration sites (EU‐PAS, ClinicalTrials.gov, ISPOR‐ISPE‐Open Science Framework RWE Registry) to address registration of comprehensive protocols for RWE studies that estimate causal effects of clinical or policy interventions. On EU‐PAS, the longest established registration site for observational studies, 57% of studies were registered without a protocol.^5^ The work stream of this task force was specifically aimed at setting expectations regarding what needs to be in a study protocol to ensure more reproducible and reliable results. However, addressing key issues in protocol registration^61^ and having policies that support it as an expectation for the field has potential to increase the ability of end‐users to evaluate RWE study quality and therefore, their utility for decision‐making.^1^, ^38^, ^43^, ^62^, ^63^

## 3 CONCLUSION

Ambiguity in communication about the design and conduct of RWE studies that make secondary use of RWD limits their utility in healthcare decision‐making. Clear communication about data provenance, design, analysis and implementation is needed. This would facilitate reproducibility, replication in independent data, and assessment of potential sources of bias.

HARPER was designed to reduce ambiguity by helping research teams be clear about the scientific decisions made in the design and conduct of an RWE study and to allow other investigators or reviewers to have a shared understanding of those decisions. It achieves this by creating a common text, tabular and visual structure so that multidisciplinary research teams and reviewers of their work will know what to look for and where to find it. The template provides a set of core expectations for clear, reproducible RWE study protocols and is intended to be used as a backbone throughout the research process from developing a study protocol, to registration, through implementation and reporting on those implementation decisions.

## CONFLICT OF INTEREST

Drs. Wang, Pottegård, Crown, Arlett, Ashcroft, Berger, Goettsch, Hua, Kurz, Orsini, Pratt, have no conflicts of interest to declare. Dr. Schneeweiss is principal investigator of the FDA Sentinel Innovation Center funded by the FDA, co‐principal investigator of an investigator‐initiated grant to the Brigham and Women's Hospital from Boehringer Ingelheim unrelated to the topic of this study. He is a consultant to Aetion Inc., a software manufacturer of which he owns equity. His interests were declared, reviewed, and approved by the Brigham and Women's Hospital and Partners HealthCare System in accordance with their institutional compliance policies. Dr. Benchimol has acted as a legal consultant for Hoffman La‐Roche Limited and Peabody & Arnold LLP and acted as a consultant to McKesson Canada for matters unrelated to this manuscript. Dr. G Crane is an employee of F. Hoffmann‐La Roche Ltd and holds stocks in F. Hoffmann‐La Roche Ltd. Dr. Kabadi is an employee and shareholder of Sanofi. Dr. Kern is an employee of Janssen R&D, and stockholder of Johnson & Johnson. Dr. Perez‐Gutthann is an employee of RTI Health Solutions, a division of the independent nonprofit Research Triangle Institute, who conducts research under contract for pharmaceutical companies, private, public organizations. Dr. Pinheiro worked on this research and article while employed by the US FDA, but is now employed by Abbvie. Dr Toussi works for IQVIA, a Human data science company who receives funds from pharmaceutical industry, governments and non‐profit organizations to conduct research. Dr. Williams worked on this research and article while employed with the National Library of Medicine, NIH, but is no longer employed with NIH. The views expressed in this article are the personal views of the authors and may not be understood or quoted as being made on behalf of or reflecting the position of the European Medicines Agency, the Food and Drug Administration, the Pharmaceutical and Medical Devices Agency or the National Institutes of Health.

## Supporting information


**Appendix 1.** Search on EQUATOR network, PubMed, snowball identification

**Appendix 2.** Additional notes on mapping of existing protocol template guidance.

**Appendix 3.** Example use cases.

**Appendix 4.** Supporting Information.
