# Supplementary material for: HARmonized Protocol Template to Enhance Reproducibility of hypothesis evaluating real‐world evidence studies on treatment effects: A good practices report of a joint ISPE/ISPOR task force
Source: Pharmacoepidemiol Drug Saf. 2022 Oct 10;32(1):44–55. doi: 10.1002/pds.5507 (PMC9771861; doi:10.1002/pds.5507)
Supplement: Supplementary file 1 — Appendix 1. Search on EQUATOR network, PubMed, snowball identification [file PDS-32-44-s003.docx]

**Appendix 1. Search on EQUATOR network, PubMed, snowball identification**

| **Seach EQUATOR network: Section of report = "Protocol-whole report"** | | |  |
| --- | --- | --- | --- |
| <https://www.equator-network.org/?post_type=eq_guidelines&eq_guidelines_study_design=0&eq_guidelines_clinical_specialty=0&eq_guidelines_report_section=protocol-whole-report&s=&btn_submit=Search+Reporting+Guidelines> | | | |
| **6 entries returned - none relevant** | |  |  |
|  |  |  |  |
|  |  | **Reason to exclude** | |
| 1 | [Standard Protocol Items for Clinical Trials with Traditional Chinese Medicine 2018: Recommendations, Explanation and Elaboration (SPIRIT-TCM Extension 2018)](https://www.equator-network.org/reporting-guidelines/standard-protocol-items-for-clinical-trials-with-traditional-chinese-medicine-spirit-tcm-extension-2018/) | Clinical trial - checklist | |
| 2 | [SPIRIT extension and elaboration for n-of-1 trials: SPENT 2019 checklist](https://www.equator-network.org/reporting-guidelines/spirit-extension-n-of-1-trials-spent-2019/) | Clinical trial - checklist | |
| 3 | [SPIRIT 2013 Statement: Defining standard protocol items for clinical trials](https://www.equator-network.org/reporting-guidelines/spirit-2013-statement-defining-standard-protocol-items-for-clinical-trials/) | Clinical trial - checklist | |
| 4 | [Preferred Reporting Items for Systematic Review and Meta-Analysis Protocols (PRISMA-P) 2015 statement](https://www.equator-network.org/reporting-guidelines/prisma-protocols/) | Systematic review - checklist | |
| 5 | [Core Outcome Set-STAndardised Protocol Items: the COS-STAP Statement](https://www.equator-network.org/reporting-guidelines/core-outcome-set-standardised-protocol-items-the-cos-stap-statement/) | Core outcome set - checklist | |
| 6 | [A protocol format for the preparation, registration and publication of systematic reviews of animal intervention studies](https://www.equator-network.org/reporting-guidelines/a-protocol-format-for-the-preparation-registration-and-publication-of-systematic-reviews-of-animal-intervention-studies/) | Systematic review of animal intervention studies | |
|  |  |  |  |
| **Search PubMed: "protocol template"** | |  |  |
| <https://pubmed.ncbi.nlm.nih.gov/?term=%22protocol+template%22+> | | |  |
| **15 entries returned - none relevant** | | | |
|  |  |  |  |
|  | Title | PMID | Excluded because |
| 1 | ASRM standard embryo transfer protocol template: a committee opinion. | 28292611 | Not a study protocol template |
| 2 | Template-ready PCR method for detection of human telomerase reverse transcriptase mRNA in sputum. | 30991019 | Not a study protocol template |
| 3 | CT and MR Protocol Standardization Across a Large Health System: Providing a Consistent Radiologist, Patient, and Referring Provider Experience. | 27448401 | Not a study protocol template |
| 4 | Development and implementation of clinical trial protocol templates at the National Institute of Allergy and Infectious Diseases. | 19625326 | Clinical trial protocol template |
| 5 | The European Organization for Research and Treatment for Cancer (EORTC) strategy for quality assurance in surgical clinical research: Assessment of the past and moving towards the future. | 27241924 | Not a study protocol template |
| 6 | Conducting a successful residency research project. | 19002290 | Not a study protocol template |
| 7 | Optimisation of pharmacy content in clinical cancer research protocols: Experience of the United Kingdom Chemotherapy and Pharmacy Advisory Service. | 25652529 | Clinical trial protocol template |
| 8 | Neurooncology clinical trial design for targeted therapies: lessons learned from the North American Brain Tumor Consortium. | 18559968 | Not a study protocol template |
| 9 | Improving economic evaluations in stroke: A report from the ESO Health Economics Working Group. | 32637652 | Not a study protocol template |
| 10 | Development and evaluation of evidence-informed clinical nursing protocols for remote assessment, triage and support of cancer treatment-induced symptoms. | 23476759 | Not a study protocol template |
| 11 | Development of clinical trial protocols involving advanced radiation therapy techniques: the European Organisation for Research and Treatment of Cancer Radiation Oncology Group approach. | 22387181 | Clinical trial protocol template |
| 12 | Routine human papillomavirus genotyping by DNA sequencing in community hospital laboratories. | 17550589 | Not a study protocol template |
| 13 | Multicenter evaluation of arbitrarily primed PCR for typing of Staphylococcus aureus strains. | 7650182 | Not a study protocol template |
| 14 | A novel cross-disciplinary multi-institute approach to translational cancer research: lessons learned from Pennsylvania Cancer Alliance Bioinformatics Consortium (PCABC). | 19455246 | Not a study protocol template |
| 15 | Should follow-up biopsies for men on active surveillance for prostate cancer be restricted to limited templates? | 23735610 | Not a study protocol template |
|  |  |  |  |
| **Search ISPE-ISPOR resources, snowball/expert identification of templates** | | |  |
|  |  |  |  |
| **What:** | **Where:** | **Last update:** | **Search strategy:** |
| ISPE GPP Section II | <https://www.pharmacoepi.org/resources/policies/guidelines-08027/> | Jun-15 | ISPE |
| EMA PASS Protocol template | <https://www.ema.europa.eu/en/documents/other/guidance-format-content-protocol-non-interventional-post-authorisation-safety-studies_en.pdf> | Sep-12 | Snowball-Expert Identification |
| STaRT-RWE | <https://dataverse.harvard.edu/dataset.xhtml?persistentId=doi%3A10.7910%2FDVN%2F6R1KCA&version=DRAFT> | Dec-20 | Snowball-Expert Identification |
| NESTcc | <https://mdic.org/wp-content/uploads/2020/02/NESTcc-Methods-Framework.pdf> | Feb-20 | Snowball-Expert Identification |
| Children’s Hospital of Philadelphia IRB protocol template | <https://secure-web.cisco.com/1CBiaI8c8yBAcPz7rKyv24uowfNw66cLD4nO_OzLk-VnhhtngsGkFc1KVjgX5Tzw5BLTOKLiiy-6g_qE8aid2jWb_Vo1tVu1m_Eyk9gibVtYu87hiryTprl250vZi5hBul0N5z9fKIbHzAWvfBtYwZmBbOgbxYgKlNjuoUoc9kAaY2BltWENIpfeS-M91skmm6wnPaE_Htuh40zCdiVUpnCgtJL6IP-r55_HcqBQUtA6K2X3Anl8mjKdY49K8pk3y/https%3A%2F%2Firb.research.chop.edu%2Fprotocol-templates> | Jul-20 | Snowball-Expert Identification |
