# Supplementary material for: HARmonized Protocol Template to Enhance Reproducibility of hypothesis evaluating real‐world evidence studies on treatment effects: A good practices report of a joint ISPE/ISPOR task force
Source: Pharmacoepidemiol Drug Saf. 2022 Oct 10;32(1):44–55. doi: 10.1002/pds.5507 (PMC9771861; doi:10.1002/pds.5507)
Supplement: Supplementary file 2 — Appendix 2. Additional notes on mapping of existing protocol template guidance. [file PDS-32-44-s001.docx]

**Appendix 2 Additional notes on mapping of existing protocol template guidance**

|  | **EMA GVP**  **Mod VIII PASS**  **(Rev 3, Oct 2017)** | **Notes** | **ISPE GPP**  **(June 2015)** | **Notes** | **NESTcc**  **(Feb 2020)** | **Notes** | **STaRT-RWE**  **(2021)** | **Notes** |
| --- | --- | --- | --- | --- | --- | --- | --- | --- |
| **High level summary** | Largely free text, with guidance on what to include under section headers - details in the *Guidance for the format and content of the protocol of non-interventional post-authorisation safety studies (www.ema.europa.eu)* | | Largely free text, with guidance on what to include under section headers - details in report https://www.pharmacoepi.org/resources/policies/guidelines-08027/ | | Largely free text, with guidance on what to include under section headers - details in NESTcc report | | Structured tables that lay out operational parameters to be specified - details in Wang et al, BMJ | |
| **Section Header** |  |  |  |  |  |  |  |  |
| Title page with administrative information  (e.g. title, registry ID, drug/device ID, sponsor) | 1 | Structured as table, include version/date, recommend registration on EU-PAS | A, C, M | Free text, include contact information of investigators and sponsors, recommend registration on EU PAS or clinicaltrials.gov | 2, 10 | Free text, recommend registration on clinicaltrials.gov or cos.io | Table 1 | Table, also includes human subjects protection approval ID, conflict of interest |
| Table of contents | 2 | List |  |  |  |  | Table of Contents | List |
| Abbreviations | 3 | List |  |  |  |  | Table 9 | Table |
| Glossary of terminology |  |  |  |  |  |  | Table 8 | Table |
| Responsible parties | 4 | Include contact information in Annex, distribution of responsibilities mentioned here | B |  |  |  | Table 1 | Table includes name, role, affiliation |
| Abstract | 5 | Structured headings | D | Free text |  |  |  |  |
| Amendments and updates | 6 | Table with date, change, and reason | L | Free text, dated amendments with rationale |  |  | Table 2 | Table with date, change, and reason |
| Milestones/timeline | 7 | Table | E | Free text |  |  |  |  |
| Rationale and background | 8 | Free text | G | Free text | 1 | Free text |  |  |
| Research question and objectives | 9 | Free text, recommend organizing as primary/secondary objectives | F | Free text, discuss exposure, outcome and limited number of a priori hypotheses, frame research questions using PICOT | 3 | Free text, order objectives by importance, be clear about primary vs secondary, specify measure of association for each outcome and type of measurement (binary, time-to-event), provide mathematical expression of hypothesis and verbal statement | Table 1 | Table requesting that research questions are framed using PICOT and separate sections provided for primary vs secondary |
| Study design | 9.1 | Free text, discuss endpoints and measure of effect, strength of design | H1 | Free text, discuss rationale for design chosen | 7 | Free text, describe and justify design, describe matching or weighting procedures if used, describe training, validation and test sets if machine learning approaches used, specify and justify subgroups, confounders (or procedure used to select confounders), rationale for categorization, characterize performance of covariates | Figure 1, Table 3 | Figure 1 summarizes study design, Table 3 walks through operational aspects of design elements |
| Setting | 9.2 | Free text, discuss source population, selection criteria and representativeness | H2 | Free text, discuss rationale for inclusion-exclusion criteria and generalizability | 4 | Free text, discuss source population, selection criteria, describe clinical centers and steps to assess data quality | Table 3A, 3B, 3C, 3D, Table 6 | Table sections cover data, operational definitions of study entry, exposure, inclusion-exclusion criteria and attrition table showing counts as exclusions are applied |
| Variables | 9.3 | Free text, provide operational definitions and specify exposures, outcomes, covariates, effect modifiers | H4 | Free text, provide clear operational definitions | 5, 6 | Free text, provide clear definitions of outcomes, characterize misclassification rate, endpoint adjudication, measures to minimize data collection bias, scale of outcome (binary, time-to-event), completeness of capture, use control outcomes, timing of follow up. Free text, describe induction and latent periods, unit of exposure, precision of measurement | Table 3B (study entry/exposure), 3E & 3F (covariates), 3G (outcome), 3H (follow up) | Table sections cover operational definitions of study entry, exposure, covariates (including subgroups and effect modifiers), outcomes, and follow up time with specific fields to specify care setting, diagnosis position, timing of measurement relative to day 0, source of algorithm. For outcomes, there is a field to specify algorithm performance or endpoint validation results. For every parameter, there are fields to indicate pre-specification and whether the parameter is varied for sensitivity analyses |
| Device description |  |  |  |  | 2 | Free text, discuss device and components (status e.g. investigational, on market), mode of action, intended use, sizing requirements and technical training, expected performance over time |  |  |
| Data sources | 9.4 | Free text, discuss data sources, validity of coding, validation of algorithms, data linkage methods | H3 | Free text, provide name of data source, use validated measures when possible and provide performance characteristics |  |  | Table 3A (data), Appendices | Table to specify characteristics of data, data transformation, linkage (reference appendix or cited materials) and software |
| Study size | 9.5 | Free text, provide assumptions used to calculate study size or precision | H5 | Free text, describe the relationship between aims and projected study size | 9 | Free text, discuss design, approach to evaluation, features that impact sample size (e.g. adjustment for multiplicity, outcome rates, censoring) | Table 7 | Table with assumptions about population and statistical assumptions to calculate sample size, power, or precision |
| Data management | 9.6 | Free text, procedures for data collection, retrieval, and statistical software | H6, H7 | Free text, discuss linkage, data mining or validation |  |  | Table 3A (data), Appendices | Table to specify characteristics of data, data transformation, linkage (reference appendix or cited materials) and software |
| Data analysis | 9.7 | Free text, discuss rationale for statistical methods, major steps from raw data to result including missing data, control of bias, sensitivity analyses, etc. | H8 | Free text, discuss data cleaning, imputation of missing, or modification of raw data, and statistical software | 12 | Free text, define and justify target population, describe data location and planned analyses, software, computation of derived variables, adjustment fo multiplicity, sensitivity analyses, shell tables | Table 4, Table 5 | Tables with sections to specify populations, subgroups, models, confounding adjustment methods. Separate table to specify sensitivity analyses, rationale and what knowledge expected to be gained |
| Quality control | 9.8 | Free text, discuss procedures to ensure data quality, e.g. certification, endpoint validation | H9 | Free text, discuss procedures to ensure data quality, e.g. certification, endpoint validation | 11 | Free text, discuss data quality and potential pathways to address issues |  |  |
| Limitations of the methods | 9.9 | Free text, discuss limitations and likely success of efforts to reduce bias | H10 | Free text, discuss limitations and likely success of efforts to reduce bias |  |  |  |  |
| Other aspects | 9.1 | Free text, anything not previously covered |  |  |  |  |  |  |
| Protection of human subjects | 10 | Free text, discuss plan for protecting human subjects | I | Free text, discuss plan for protecting human subjects | 8 | Free text, discuss informed consent, human subjects | Table 1 | Field in table of administrative information |
| Management and reporting of adverse events | 11 | Free text, discuss collection/management/reporting of AE |  |  |  |  |  |  |
| Plans for disseminating and communicating study results | 12 | Free text, discuss submission of progress and final reports | J | Free text, describe plans for communicating results, include presence/absence of restrictions on publication |  |  |  |  |
| References | 13 | Numbered list | K | Numbered list |  |  |  |  |
| Appendices | Annex | Table listing stand alone documents, additional information |  |  |  |  | Appendices | Tables and free text with machine readable code lists for algorithms used, details on data linkage, data cleaning/conversion, etc. |
| ENCePP Checklist for study protocols | Annex | Checklist |  |  |  |  |  |  |
