# Supplementary material for: HARmonized Protocol Template to Enhance Reproducibility of hypothesis evaluating real‐world evidence studies on treatment effects: A good practices report of a joint ISPE/ISPOR task force
Source: Pharmacoepidemiol Drug Saf. 2022 Oct 10;32(1):44–55. doi: 10.1002/pds.5507 (PMC9771861; doi:10.1002/pds.5507)
Supplement: Supplementary file 3 — Appendix 3. Example use cases. [file PDS-32-44-s004.zip › Appendix 3/Appendix 3 Summary.docx]

**Appendix 3 Example use cases**

Five examples of using the HARPER template with different use cases are enclosed.

| **Files** | **Use case** | **Question** | **What is unique** |
| --- | --- | --- | --- |
| Example 1 Comparative Eff (word)  Example 1 appendices (excel) | Empagliflozin versus DPP4i on 3P-MACE | Effectiveness | Existing protocol was reconstructed with more details added to match expectations in harmonized template (cohort study) |
| Example 2 Cancer new vs standard of care (word) | New cancer therapy compared to standard of care | Effectiveness | Harmonized template used for new protocol in development (cohort study) |
| Example 3 Case-control (word) | Pioglitazone and risk of bladder cancer | Safety | Nested case-control design |
| Example 4 Pregnancy Safety (word) | Topiramate and oral clefts | Safety | Pregnancy cohort study with more complexity in design parameters |
| Example 5 SCCS effectiveness (word) | Palivizumab and RSV | Effectiveness | Self-controlled design |
