# Supplementary material for: HARmonized Protocol Template to Enhance Reproducibility of hypothesis evaluating real‐world evidence studies on treatment effects: A good practices report of a joint ISPE/ISPOR task force
Source: Pharmacoepidemiol Drug Saf. 2022 Oct 10;32(1):44–55. doi: 10.1002/pds.5507 (PMC9771861; doi:10.1002/pds.5507)
Supplement: Supplementary file 3 — Appendix 3. Example use cases. [file PDS-32-44-s004.zip › Appendix 3/Example 1 Comparative Eff.docx]

DISCLAIMER: This protocol example is an abbreviated version based on a published protocol developed for another study (https://clinicaltrials.gov/ct2/show/NCT04215536).

# Title Page

| Title | Empagliflozin versus DPP-4 Inhibitor on risk of 3P MACE |
| --- | --- |
| Research question & Objectives | The objective is to evaluate the comparative risk of 3P MACE for initiators of empagliflozin versus DPP4-inhibitors |
| Protocol version | V3 |
| Last update date | 2020-01-02 |
| Contributors | **Primary Investigator contact information:**  RCT_DUPLICATE@bwh.harvard.edu  **Contributor names:**  DUPLICATE Team |
| Study registration | Site: clinicaltrials.gov  **Identifier:** NCT04215536 |
| Sponsor | Organization: Food and Drug Administration  **Contact:** anonymous@fda.hhs.gov |
| Conflict of interest | n/a |

Table of contents

[1. Title Page 1](#_Toc102631342)

[2. Abstract 3](#_Toc102631343)

[3. Amendments and updates 4](#_Toc102631344)

[4. Milestones 5](#_Toc102631345)

[Table 1 Milestones 5](#_Toc102631346)

[5. Rationale and background 5](#_Toc102631347)

[6. Research question and objectives 5](#_Toc102631348)

[Table 2 Primary and secondary research questions and objective 5](#_Toc102631349)

[7. Research methods 7](#_Toc102631350)

[7.1. Study design 7](#_Toc102631351)

[7.2. Study design diagram 7](#_Toc102631352)

[7.3. Setting 8](#_Toc102631353)

[7.3.1 Context and rationale for definition of time 0 (and other primary time anchors) for entry to the study population 8](#_Toc102631354)

[Table 3 Operational Definition of Time 0 (index date) and other primary time anchors 8](#_Toc102631355)

[7.3.2 Context and rationale for study inclusion criteria: 9](#_Toc102631356)

[Table 4. Operational Definitions of Inclusion Criteria 9](#_Toc102631357)

[7.3.3 Context and rationale for study exclusion criteria 10](#_Toc102631358)

[Table 5. Operational Definitions of Exclusion Criteria 10](#_Toc102631359)

[7.4. Variables 11](#_Toc102631360)

[7.4.1 Context and rationale for exposure(s) of interest 11](#_Toc102631361)

[Table 6. Operational Definitions of Exposure 11](#_Toc102631362)

[7.4.2 Context and rationale for outcome(s) of interest 12](#_Toc102631363)

[Table 7. Operational Definitions of Outcome 12](#_Toc102631364)

[7.4.3 Context and rationale for follow up 13](#_Toc102631365)

[Table 8. Operational Definitions of Follow Up 13](#_Toc102631366)

[7.4.4 Context and rationale for covariates (confounding variables and effect modifiers, e.g. risk factors, comorbidities, comedications) 14](#_Toc102631367)

[Table 9. Operational Definitions of Covariates 14](#_Toc102631368)

[7.5. Data analysis 14](#_Toc102631369)

[7.5.1 Context and rationale for analysis plan 14](#_Toc102631370)

[Table 10. Primary, secondary, and subgroup analysis specification 15](#_Toc102631371)

[Table 11. Sensitivity analyses – rationale, strengths and limitations 16](#_Toc102631372)

[7.6. Data sources 16](#_Toc102631373)

[7.6.1 Context and rationale for data sources 16](#_Toc102631374)

[Table 12. Metadata about data sources and software 17](#_Toc102631375)

[7.7. Data management 17](#_Toc102631376)

[7.8. Quality control 18](#_Toc102631377)

[7.9. Study size and feasibility 18](#_Toc102631378)

[Table 13. Power and sample size 18](#_Toc102631379)

[8. Limitation of the methods 19](#_Toc102631380)

[9. Protection of human subjects 19](#_Toc102631381)

[10. Reporting of adverse events 20](#_Toc102631382)

[11. References 20](#_Toc102631383)

[12. Appendices 20](#_Toc102631384)

1. Abstract

The risk of cardiovascular disease is elevated in patients with Type 2 diabetes. Empagliflozin is a selective inhibitor of SGLT2. In trials where empagliflozin has been used as monotherapy or add on therapy, it has improved hemoglobin A1c, reduced body weight, lowered blood pressure, and affected cholesterol levels. There has been suggestion of improvement in visceral fat mass and proteinurea. The EMPA-REG trial found a 15% reduction in 3P-MACE (a composite of nonfatal stroke, nonfatal myocardial infarction, and cardiovascular death) for empagliflozin compared to placebo.^1^

The magnitude of the beneficial effect of empagliflozin compared to an alternative diabetes therapy used at the same stage in clinical practice for the outcome of 3P MACE is unknown. This study will evaluate the effectiveness of empagliflozin at reducing risk of 3P MACE in diabetic patients in clinical practice, when compared to an alternative treatment of DPP4 inhibitors, a class of medications that is used at a similar stage of diabetes as empagliflozin.

We will use 3 national United States claims databases that are used extensively for research (Optum Clinformatics, IBM MarketScan, Medicare). The selected data sources are widely used for research and the data holders provide thorough documentation of data contents, assumptions and limitations. MarketScan is a database of >100 commercial health insurers that provide comprehensive coverage for over 25 million members annually with active policies located throughout the US.^2^ The Optum database comprises a large, geographically diverse population of health insurance beneficiaries enrolled in commercial UnitedHealth Group-affiliated health plans. Medicare data includes administrative claims for older adults across the United States. Each data source contains longitudinal, date stamped information on patient enrolment, demographics, in and outpatient diagnoses, procedures, admission and discharge dates, and medication dispensing that can be used to capture exposure, key inclusion-exclusion criteria, outcome, and covariates. The data are derived from claims for services received by the patient. MarketScan and Optum are representative of national employer based insured populations. The vast majority of adults over 65 in the US are enrolled in Medicare.

1. Amendments and updates

| **Version date** | **Version number** | **Section of protocol** | **Amendment or update** | **Reason** |
| --- | --- | --- | --- | --- |
| 2019-11-04 | 1 | All | First feasibility counts generated | Stopped after 1^st^ feasibility counts generated for review by team members and revision as necessary. |
| 2019-11-30 | 2 | Second feasibility | Counts revised after propensity score matching. Outcome counts not stratified by exposure. Matched Table 1’s generated | Stopped after 2^nd^ feasibility counts generated for review by team members and revision as necessary. |
| 2020-01-02 | 3 | Final protocol | Covariates revised and counts regenerated. Appendices including operational definitions attached. | Next step in process of creating protocol. Moving toward analysis. |
| 2019-11-04 | 1 | All | First feasibility counts generated | Stopped after 1^st^ feasibility counts generated for review by team members and revision as necessary. |

1. Milestones

#### Table 1 Milestones

| **Milestone** | **Date** |
| --- | --- |
| First feasibility counts | 2019-11-04 |
| Second feasibility counts with balance diagnostics | 2019-11-30 |
| Final protocol | 2020-01-02 |
| Analysis completion | 2020-01-16 |

1. Rationale and background

**What is known about the condition:** The risk of cardiovascular disease is elevated in patients with Type 2 diabetes.^3^

**What is known about the exposure of interest:** Empagliflozin is a selective inhibitor of SGLT2. In trials where empagliflozin has been used as monotherapy or add on therapy, it has improved hemoglobin A1c, reduced body weight, lowered blood pressure, and affected cholesterol levels. There has been suggestion of improvement in visceral fat mass and proteinurea. The EMPA-REG trial found a 15% reduction in 3P-MACE (a composite of nonfatal stroke, nonfatal myocardial infarction, and cardiovascular death) for empagliflozin compared to placebo.^1^

**Gaps in knowledge:** The magnitude of the beneficial effect of empagliflozin compared to an alternative diabetes therapy used at the same stage in clinical practice for the outcome of 3P MACE is unknown.

**What is the expected contribution of this study?** This study will evaluate the effectiveness of empagliflozin at reducing risk of 3P MACE in diabetic patients when compared to an alternative treatment of DPP4 inhibitors, a class of medications that is used at a similar stage of diabetes as empagliflozin.

1. Research question and objectives

#### Table 2 Primary and secondary research questions and objective

1. **Primary research question and objective**

| **Objective:** | To evaluate the risk of 3P MACE with empagliflozin compared to DPP4 inhibitors. |
| --- | --- |
| **Hypothesis:** | Empagliflozin reduces the risk of 3P MACE compared to DPP4 inhibitors. |
| **Population *(mention key inclusion-exclusion criteria):*** | Type 2 diabetic patients with existing cardiovascular disease who are at high risk of cardiovascular events. |
| **Exposure:** | Empagliflozin |
| **Comparator:** | DPP4-inhibitors |
| **Outcome:** | 3P MACE |
| **Time *(when follow up begins and ends):*** | Follow up from the day after initiation of therapy until the first of outcome, discontinuation, add/switch therapy, disenrollment, end of study period, nursing home admission. |
| **Setting:** | Outpatient, ambulatory patients |
| **Main measure of effect:** | Hazard Ratio |

1. **Secondary research question 1 and objective**

| **Objective:** | To evaluate the risk of the component of 3P MACE with empagliflozin compared to DPP4 inhibitors. |
| --- | --- |
| **Hypothesis:** | Empagliflozin reduces the risk of the components of 3P MACE compared to DPP4 inhibitors. |
| **Population *(mention key inclusion-exclusion criteria):*** | Type 2 diabetic patients with existing cardiovascular disease who are at high risk of cardiovascular events. |
| **Exposure:** | Empagliflozin |
| **Comparator:** | DPP4-inhibitors |
| **Outcome:** | Individual components of 3P MACE, e.g. hospital admission for MI, hospital admission for stroke, mortality (we will assume that all deaths in this high risk population are related to cardiovascular causes) |
| **Time *(when follow up begins and ends):*** | Follow up from the day after initiation of therapy until the first of outcome, discontinuation, add/switch therapy, disenrollment, end of study period, nursing home admission. |
| **Setting:** | Outpatient, ambulatory patients |
| **Main measure of effect:** | Hazard Ratio |

1. Research methods
   1. Study design

**Research design (e.g. cohort, case-control, etc.):** New user active comparator cohort study

**Rationale for study design choice:** This study design reduces risk of bias from unmeasured time varying confounding by indication and fits neatly into the target trial framework.

- 1. Study design diagram


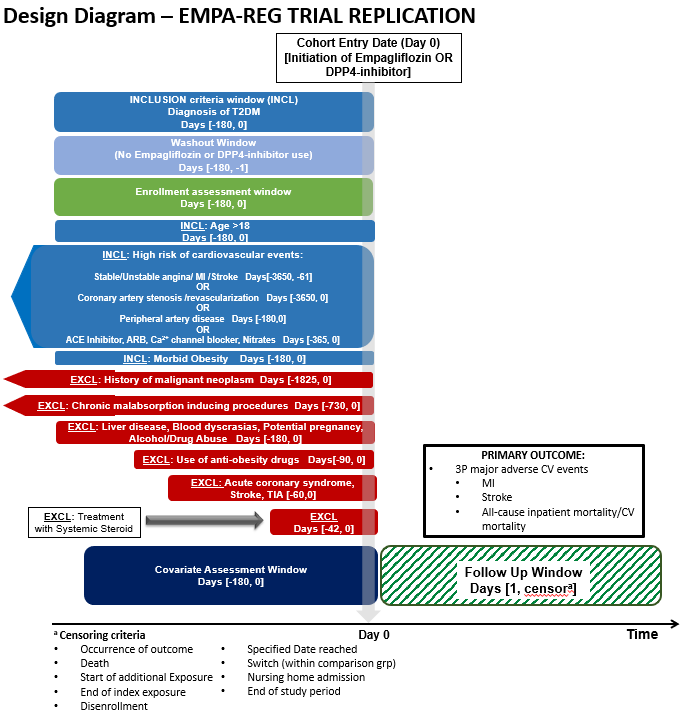


- 1. Setting

#### 7.3.1 Context and rationale for definition of time 0 (and other primary time anchors) for entry to the study population

Time 0 is the date of initiation of empagliflozin or DPP4 inhibitor. This is when patients enter the study population and mimics the timing of initiation of therapy at randomization in the target trial framework.

#### Table 3 Operational Definition of Time 0 (index date) and other primary time anchors

| **Study population name(s)** | **Time Anchor Description**  **(e.g. time 0)** | **Number of entries** | **Type of entry** | **Washout window** | **Care Setting^1^** | **Code Type^2^** | **Diagnosis position** | **Incident with respect to…** | **Measurement characteristics/**  **validation** | **Source of algorithm** |
| --- | --- | --- | --- | --- | --- | --- | --- | --- | --- | --- |
| Exposure:  Empagliflozin | Date of incident dispensation for Empagliflozin (time 0) | Single | Incident | [-180, -1] | n/a | NDC | n/a | Empagliflozin or DPP4i (any formulation of either) | No validation study | Investigator review of generic names |
| Comparator:  DPP4i | Date of incident dispensation for DPP4i  (time 0) | Single | Incident | [-180, -1] | n/a | NDC | n/a | Empagliflozin or DPP4i (any formulation of either) | No validation study | Investigator review of generic names |

^1^ IP = inpatient, OP = outpatient, ED = emergency department, OT = other, n/a = not applicable

^2^See appendix for listing of clinical codes for each study parameter

#### 7.3.2 Context and rationale for study inclusion criteria:

We require 6 months of medical and drug coverage prior to time 0 in order to ensure that patients have observable time in the data where contact with the healthcare system will allow capture of clinical codes to measure inclusion-exclusion criteria and covariates. We restrict the population to adult patients with type 2 diabetes at high risk of cardiovascular outcomes without evidence of morbid obesity.

#### Table 4. Operational Definitions of Inclusion Criteria

| **Criterion** | **Details** | **Order of application** | **Assessment window** | **Care Settings¹** | **Code Type^2^** | **Diagnosis position^3^** | **Applied to study populations:** | **Measurement characteristics/**  **validation** | **Source for algorithm** |
| --- | --- | --- | --- | --- | --- | --- | --- | --- | --- |
| Observable time | Medical and drug coverage (maximum allowable gap in coverage = 30 days) | Before selection of index date | [-180, 0] | n/a | n/a | n/a | Exposure: Empagliflozin, Comparator: DPP4-I |  | n/a |
| Age ≥18 yrs |  | Before selection of index date | [0, 0] | n/a | n/a | n/a | Exposure: Empagliflozin, Comparator: DPP4-I |  | n/a |
| Type 2 Diabetes diagnosis |  | Before selection of index date | [-180, 0] | Any | ICD-9-CM | Any | Exposure: Empagliflozin, Comparator: DPP4-I | No validation study | Patorno, E et al. BMJ 2018^4^ Patorno, E et al. Circulation, 2019^5^ |
| High risk of cardiovascular events | Defined by occurrence of ≥1 of the following 4 criteria: |  |  |  |  |  |  |  |  |
| High risk of CV event 1: | Stable/Unstable angina OR MI OR Stroke | Before selection of index date | [-365, -61] | Any | ICD-9-CM | Any | Exposure: Empagliflozin, Comparator: DPP4-I | No validation study | Investigator review of clinical codes |
| High risk of CV event 2: | Coronary artery stenosis/revascularization | Before selection of index date | [-365, 0] | Any | ICD-9-CM | Any | Exposure: Empagliflozin, Comparator: DPP4-I | No validation study | Patorno, E et al. BMJ 2018^4^ Patorno, E et al. Circulation, 2019^5^ |
| High risk of CV event 3: | Peripheral artery disease | Before selection of index date | [-180, 0] | Any | ICD-9-CM | Any | Exposure: Empagliflozin, Comparator: DPP4-I | No validation study | Patorno, E et al. BMJ 2018^4^ Patorno, E et al. Circulation, 2019^5^ |
| High risk of CV event 4: | Dispensing of one of the following: ACE inhibitor, ARB, Calcium Channel blocker, Nitrates | Before selection of index date | [-365, 0] | Any | ICD-9-CM | Any | Exposure: Empagliflozin, Comparator: DPP4-I | No validation study | Patorno, E et al. BMJ 2018^4^ Patorno, E et al. Circulation, 2019^5^ |
| No morbid obesity (simple) | Proxy for inclusion of patients with BMI <45 kg/m^2 | Before selection of index date | [-180, 0] | Any | ICD-9-CM | Any | Exposure: Empagliflozin, Comparator: DPP4-I | No validation study | n/a |

^1^ IP = inpatient, OP = outpatient, ED = emergency department, OT = other, n/a = not applicable

^2^ See appendix for listing of clinical codes for each study parameter

^3^ Specify whether a diagnosis code is required to be in the primary position (main reason for encounter)

#### 7.3.3 Context and rationale for study exclusion criteria

We exclude patients with missing age or sex as well as apply exclusion criteria mimicking those of the EMPA-REG trial in order to emulate the trial design.

#### Table 5. Operational Definitions of Exclusion Criteria

| **Criterion** | **Details** | **Order of application** | **Assessment window** | **Care Settings¹** | **Code Type^2^** | **Diagnosis position^3^** | **Applied to study populations:** | **Measurement characteristics/**  **validation** | **Source for algorithm** |
| --- | --- | --- | --- | --- | --- | --- | --- | --- | --- |
| Age missing |  | Before selection of index date | [0, 0] | n/a | n/a | n/a | Exposure: Empagliflozin, Comparator: DPP4-I |  | n/a |
| Sex missing/unkown |  | Before selection of index date | [0, 0] | n/a | n/a | n/a | Exposure: Empagliflozin, Comparator: DPP4-I |  | n/a |
| Liver Disease |  | Before selection of index date | [-180, 0] | Any | ICD-9-CM | Any | Exposure: Empagliflozin, Comparator: DPP4-I | No validation study | Patorno, E et al. BMJ 2018^4^ Patorno, E et al. Circulation, 2019^5^ |
| Chronic malabsorption inducing procedures | (i.e. bariatric/gastric bypass surgery) | Before selection of index date | [-730, -1] | Any | ICD-9-CM | Any | Exposure: Empagliflozin, Comparator: DPP4-I | No validation study | Hatoum, Ida J, et al. JAMA Surg, 2016^6^ |
| Blood dyscrasias |  | Before selection of index date | [-180, 0] | Any | ICD-9-CM | Any | Exposure: Empagliflozin, Comparator: DPP4-I | No validation study | Investigator review of clinical codes |
| History of malignant neoplasm |  | Before selection of index date | [-1825,-1] | Any | ICD-9-CM | Any | Exposure: Empagliflozin, Comparator: DPP4-I | No validation study | Patorno, E et al. BMJ 2018^4^ Patorno, E et al. Circulation, 2019^5^ |
| Dispensing of of anti-obesity drug OR morbid obesity claim |  | Before selection of index date | [-90, -1] | Any | Other | Any | Exposure: Empagliflozin, Comparator: DPP4-I | No validation study | Patorno, E et al. BMJ 2018^4^ Patorno, E et al. Circulation, 2019^5^ |
| Dispensing of corticosteroids |  | Before selection of index date | [-42, -1] | Any | NDC | n/a | Exposure: Empagliflozin, Comparator: DPP4-I | No validation study | Patorno, E et al. BMJ 2018^4^ Patorno, E et al. Circulation, 2019^5^ |
| Pregnancy or dispensing of contraceptive |  | Before selection of index date | [-180, 0] | Any | ICD-9-CM | Any | Exposure: Empagliflozin, Comparator: DPP4-I | No validation study | Krumme, AA, et. al. J. Comp. Eff. Res. (2018)^7^ |
| Alcohol or drug abuse |  | Before selection of index date | [-180, 0] | Any | ICD-9-CM | Any | Exposure: Empagliflozin, Comparator: DPP4-I | No validation study | Patorno, E et al. BMJ 2018^4^ Patorno, E et al. Circulation, 2019^5^ |

^1^ IP = inpatient, OP = outpatient, ED = emergency department, OT = other, n/a = not applicable

^2^ See appendix for listing of clinical codes for each study parameter

^3^ Specify whether a diagnosis code is required to be in the primary position (main reason for encounter)

- 1. Variables

#### 7.4.1 Context and rationale for exposure(s) of interest

We focus on new initiators to avoid bias related to depletion of the susceptible and confounding by time varying indication. The use of an active comparator of DPP4 inhibitors will allow us to compare initiators who are in need of a similar line of therapy and at a similar stage of diabetes disease severity.

**Algorithm to define duration of exposure effect:**

If a refill occurs before the end of days supply dispensed, add overlapping days to the end of the subsequent dispensing’s day supply. Assume that the effect of a pill lasts for 30 days. Therefore, we allow up to a 30 day gap between a dispensation + days supply and refill. We also add 30 days to the last dispensation + days supply in a treatment episode and consider this “exposed” time.

#### Table 6. Operational Definitions of Exposure

| **Exposure group name(s)** | **Details** | **Washout window** | **Assessment Window** | **Care Setting^1^** | **Code Type^2^** | **Diagnosis position^3^** | **Applied to study populations:** | **Incident with respect to…** | **Measurement characteristics/ validation** | **Source of algorithm** |
| --- | --- | --- | --- | --- | --- | --- | --- | --- | --- | --- |
| Exposure |  | [-183, 0] | [1, censor] | n/a | NDC | n/a | Exposure: Empagliflozin, Comparator: DPP4-I | Drug A or B  (any formulation) | No validation study | Investigator review of generic names |
| Comparator |  | [-183, 0] | [1, censor] | n/a | NDC | n/a | Exposure: Empagliflozin, Comparator: DPP4-I | Drug A or B  (any formulation) | No validation study | Investigator review of generic names |

^1^ IP = inpatient, OP = outpatient, ED = emergency department, OT = other, n/a = not applicable

^2^ See appendix for listing of clinical codes for each study parameter

^3^ Specify whether a diagnosis code is required to be in the primary position (main reason for encounter)

#### 7.4.2 Context and rationale for outcome(s) of interest

The outcome of 3-point major cardiovascular outcome (3PMACE) was selected to evaluate the potential cardioprotective effects of empagliflozin. This outcome parallels the outcome used in the EMPA-REG trial comparing empagliflozin to placebo.

#### Table 7. Operational Definitions of Outcome

| **Outcome name** | **Details** | **Primary outcome?** | **Type of outcome** | **Washout window** | **Care Settings¹** | **Code Type^2^** | **Diagnosis Position^3^** | **Applied to study populations:** | **Measurement characteristics/**  **validation** | **Source of algorithm** |
| --- | --- | --- | --- | --- | --- | --- | --- | --- | --- | --- |
| 3-P MACE (Composite outcome of Mortality/MI/Stroke) |  | Yes | Time-to-event | n/a | IP | ICD-9-CM | Primary | Exposure: Empagliflozin, Comparator: DPP4-I | (Components below) | See components below |
| Myocardial infarction |  | No | Time-to-event | n/a | IP | ICD-9-CM | Primary | Exposure: Empagliflozin, Comparator: DPP4-I | ➜PPV 94% in Medicare claims data ➜PPV 88.4% in commercially-insured population | Kiyota Y, American heart journal 2004^8^ Wahl PM, Pharmacoepidemiology and Drug Safety 2010^9^ |
| Stroke |  | No | Time-to-event | n/a | IP | ICD-9-CM | Primary | Exposure: Empagliflozin, Comparator: DPP4-I | PPV of 85% or higher for ischemic stroke PPV ranging from 80% to 98% for hemorrhagic stroke | Tirschwell DL, Stroke; a journal of cerebral circulation 2002^10^ |
| Mortality |  | Yes | Time-to-event | n/a | n/a | n/a | n/a | Exposure: Empagliflozin, Comparator: DPP4-I |  | Death in vital status file, discharged dead from hospital |

^1^ IP = inpatient, OP = outpatient, ED = emergency department, OT = other, n/a = not applicable

^2^ See appendix for listing of clinical codes for each study parameter

^3^ Specify whether a diagnosis code is required to be in the primary position (main reason for encounter)

#### 7.4.3 Context and rationale for follow up

We focus on an on-treatment analysis as the primary analysis to emulate the intention-to-treat estimate from EMPA-REG, which had very high adherence to randomized therapy.

#### Table 8. Operational Definitions of Follow Up

|  |  |  |  |
| --- | --- | --- | --- |
| **Follow up start** | Day 1 |  |  |
| **Follow up end^1^** | **Select all that apply** |  | **Specify** |
| **Date of outcome** | Yes |  | See Table 5 |
| **Date of death** | Yes |  | Discharged dead or Social Security Death Index, whichever came 1st |
| **End of observation in data** | Yes |  | Allow 30 day gaps in enrolment |
| **Day X following index date**  *(specify day)* | Yes |  | Day 183 |
| **End of study period**  (specify date) | Yes |  | 30-Sep-15 |
| **End of exposure**  *(specify operational details,*  *e.g. stockpiling algorithm, grace period)* | Yes |  | **Stockpiling algorithm:** If refill occurs before end of days supply, count overlapping days at the end of the subsequent dispensing’s day supply.  **Grace period:** Bridge gaps of ≤30 days between dispensation + days supply and refill. Add 30 days to last dispensation + days supply in a treatment episode. |
| **Date of add to/switch from exposure**  *(specify algorithm)* | Yes |  | Date that patient in exposed group is dispensed comparator drug or vice versa |
| **Other date** *(specify)* | Yes |  | Nursing home admission |

^1^ Follow up ends at the first occurrence of any of the selected criteria that end follow up.

#### 7.4.4 Context and rationale for covariates (confounding variables and effect modifiers, e.g. risk factors, comorbidities, comedications)

We identified demographic, comorbidity, healthcare utilization, frailty and socioeconomic status related risk factors for 3P MACE that were associated with exposure to empagliflozin versus DPP4 inhibitors.

#### Table 9. Operational Definitions of Covariates

| **Characteristic** | **Details** | **Type of variable** | **Assessment window** | **Care Settings¹** | **Code Type^2^** | **Diagnosis Position^3^** | **Applied to study populations:** | **Measurement characteristics/**  **validation** | **Source for algorithm** |
| --- | --- | --- | --- | --- | --- | --- | --- | --- | --- |
| Age | (cohort entry year - year of birth) | Continuous | [0,0] | n/a | n/a | n/a | Exposure: Empagliflozin, Comparator: DPP4-I | No validation study | n/a |
| Gender | Male, Female | Categorical | [0, 0] | n/a | n/a | n/a | Exposure: Empagliflozin, Comparator: DPP4-I | No validation study | n/a |
| Region | Northeast, South, Midwest, West | Categorical | [0, 0] | n/a | n/a | n/a | Exposure: Empagliflozin, Comparator: DPP4-I | No validation study | n/a |
| … |  |  |  |  |  |  |  |  |  |

^1^ IP = inpatient, OP = outpatient, ED = emergency department, OT = other, n/a = not applicable

^2^ See appendix for listing of clinical codes for each study parameter

^3^ Specify whether a diagnosis code is required to be in the primary position (main reason for encounter)

- 1. Data analysis

#### 7.5.1 Context and rationale for analysis plan

We use logistic regression to estimate a propensity score and then nearest neighbor match with a caliper of 0.025 on the probability scale. In the matched population, we estimate the hazard ratio for empagliflozin versus DPP4 inhibitor on 3PMACE using a Cox proportional hazards model. As a secondary analysis, we evaluate the hazard ratio for the outcome of myocardial infarction. We conduct sensitivity analyses including a control outcome and varying the lookback window to assess covariates.

#### Table 10. Primary, secondary, and subgroup analysis specification

1. **Primary analysis**

| **Hypothesis:** | Exposure to empagliflozin reduces the risk of 3P-MACE relative to DPP4-inhibitors with the upper bound of the 95% confidence interval for the hazard ratio below 1.0. |
| --- | --- |
| **Exposure contrast:** | Empagliflozin vs DPP4-inhibitor |
| **Outcome:** | 3P-MACE |
| **Analytic software:** | SAS 9.4: PHREG, PROC LOGISTIC, Pharmacoepi Toolbox nearest neighbor matching macro (http://www.drugepi.org/dope-downloads/) |
| **Model(s):**  ***(provide details or code)*** | *Outcome model:* Cox proportional hazards  followuptime*status(0) = exposure  *Propensity score model*: logistic regression  Exposure = Metastatic cancer + Tumor + Arrhythmia + Congestive heart failure + Dementia + Renal failure + Weight loss + Hemiplegia + Alcohol abuse + Pulmonary disease + Coagulopathy + Complicated diabetes + Anemia + Fluid and electrolyte disorder + Liver disease + Peripheral vascular disorder + Psychosis + Pulmonary circulation disorders + HIV/AIDS + Hypertension + Degenerative disease of central nervous system + Durable medical equipment + Number of inpatient hospitalizations + Number of outpatient visits + Number of emergency department visits + Number of unique generics |
| **Confounding adjustment method** | ***Name method and provide relevant details, e.g. bivariate, multivariable, propensity score matching (specify matching algorithm ratio and caliper), propensity score weighting (specify weight formula, trimming, truncation), propensity score stratification (specify strata definition), other.*** |
|  | We will use logistic regression to estimate a propensity score using the covariates listed in the propensity score model above and then nearest-neighbor match with a caliper of 0.025 on the probability scale using the nearest neighbor algorithm from the Pharmacoepi Toolbox. |
| **Missing data methods** | ***Name method and provide relevant details, e.g. missing indicators, complete case, last value carried forward, multiple imputation (specify model/variables), other.*** |
|  | Our primary analysis is a complete case analysis. Patients with missing or unknown sex are excluded. We assume that absence of clinical codes for a condition or procedure in the claims record means the patient did not have the condition or procedure. |
| **Subgroup Analyses** | ***List all subgroups*** |
|  | 1. Sex (male, female) 2. History of myocardial infarction (yes, no) |

#### Table 11. Sensitivity analyses – rationale, strengths and limitations

|  | **What is being varied? How?** | **Why?  (What do you expect to learn?)** | **Strengths of the sensitivity analysis compared to the primary** | **Limitations of the sensitivity analysis compared to the primary** |
| --- | --- | --- | --- | --- |
| Sensitivity Analysis 1 | We change the prior enrolment, covariate and inclusion/exclusion windows from 180 days to 365 days | We learn whether a longer assessment window to more fully capture baseline conditions results in similar estimated effect | Potentially more complete capture of baseline conditions used for inclusion-exclusion or covariate adjustment | Loss of sample size due to turnover in enrolment with health plan |
| Sensitivity Analysis 2 | We will analyse a positive control outcome (diabetic ketoacidosis) | We learn whether we can detect an expected effect for another outcome. | If results are positive, this could strengthen the argument for the causal effect of empagliflozin vs DPP4 on 3P-MACE.  If results are not positive, the magnitude of observed effect could be used to calibrate the effect of empagliflozin vs DPP4 on 3P-MACE. | The confounding structure may not be the same for diabetic ketoacidosis as 3P-MACE.  If that is the case, absence of detecting a positive effect from the analysing the positive control outcome provides less reassurance of the casual effect for empagliflozin vs DPP4 on 3P-MACE and could increase bias is used to calibrate the effect size. |

- 1. Data sources

#### 7.6.1 Context and rationale for data sources

**Reason for selection:** We selected 3 national United States claims databases that are used extensively for research (Optum Clinformatics, IBM MarketScan, Medicare). MarketScan is a database of >100 commercial health insurers that provide comprehensive coverage for over 25 million members annually with active policies located throughout the US.^2^ The Optum database comprises a large, geographically diverse population of health insurance beneficiaries enrolled in commercial UnitedHealth Group-affiliated health plans. Medicare data includes administrative claims for older adults across the United States.

**Strengths of data source(s):** Each data source contains longitudinal, date stamped information on patient enrolment, demographics, in and outpatient diagnoses, procedures, admission and discharge dates, and medication dispensing that can be used to capture exposure, key inclusion-exclusion criteria, outcome, and covariates. The data are derived from claims for services received by the patient. MarketScan and Optum are representative of national employer based insured populations. The vast majority of adults over 65 in the US are enrolled in Medicare.

**Limitations of data source(s):** There is limited clinical information. The data sources have limited or no laboratory values, vital signs, clinical notes or reports to capture study parameters. Of particular note, baseline hBA1c values are largely unavailable. Inpatient medication dispensing data are unavailable. Conditions must be diagnosed to appear on claims. Services that are not covered by insurance do not appear in the data.

**Data source provenance/curation:** The selected data sources are widely used for research and the data holders provide thorough documentation of data contents, assumptions and limitations.^11-13^

#### Table 12. Metadata about data sources and software

|  | **Data 1** | **Data 2** | **Data 3** |  |
| --- | --- | --- | --- | --- |
| **Data Source(s):** | IBM MarketScan Research Database | Optum Clinformatics | Medicare | |
| **Study Period:** | Aug 1, 2014-Dec 31, 2017 | Aug 1, 2014-Mar 31, 2019 | Aug 1, 2014-Dec 31, 2017 | |
| **Eligible Cohort Entry Period:** | Aug 1, 2014-Dec 31, 2017 | Aug 1, 2014-Mar 31, 2019 | Aug 1, 2014-Dec 31, 2017 | |
| **Data Version (or date of last update):** | January 1, 2018 | V7 | n/a | |
| **Data sampling/extraction criteria:** | All enrollees in data source | All enrollees in data source | Selected on the basis of presence of at least one diabetes diagnosis between 2012 and 2017. | |
| **Type(s) of data:** | Commercial claims | Commercial claims | Administrative claims | |
| **Data linkage:** | None | None | None | |
| **Conversion to CDM*:** | n/a | n/a | n/a | |
| **Software for data management:** | Aetion Platform Version r3.19.20191213_2104 | Aetion Platform Version r3.19.20191213_2104 | Aetion Platform Version r3.19.20191213_2104 | |

*CDM = Common Data Model

- 1. Data management

The research team operates a secure, state-of-the-art, computing facility. The computer cluster is Linux-based and offers SAS 9.4, Stata 15.1, and R. The data center is a secure facility that houses both our computing environment as well as clinical systems and electronic medical records for several large hospitals in Boston, MA. Entry into the computer room requires passing through staffed building security, a successful palm scan, and then passing through staffed computer room security. The research machines are connected to the networking backbone with 10 gigabit-per-second fiber links. Network security is overseen by Information Security, who apply the same standards used for the hospitals electronic medical records systems to the research teams data. All data are transmitted to programmers' workstations in an encrypted state. Backups are created using 256-bit AES encryption, the current Department of Defense standard for data security, and are stored in a locked facility.

The Data Manager will securely download data from the vendors to Division servers via secure SFTP. Data location, contents and data use agreements will be logged. Access to the servers are strictly controlled via physical and technical means to ensure that only individuals with proper clearance and authorization are able to access research data. When a project is closed, the research data are destroyed using a “shred” secure file deletion tool to ensure that sensitive data can never be retrieved.

ata cleaning and descriptive analyses were performed in IBM

SPSS (version 23). Regression models were developed in Stata Corp.

STATA (version 14.1).

ata cleaning and descriptive analyses were performed in IBM

SPSS (version 23). Regression models were developed in Stata Corp.

STATA (version 14.1).

Data cleaning will be performed with SAS 9.4. Feasibility, descriptive and regression analyses will be conducted with the Aetion Evidence Platform® (2021) v4.2.

- 1. Quality control

The data sources have been through extensive quality control procedures and documentation of the data is provided by the vendor. When new data is received from a vendor, the research group has an internal quality check process which includes assessment of reliability and conformance to expected plausible values. Issues are flagged for review by the data quality team and resolved with documentation of decisions made to clean the data before it is released to the research team to conduct studies.

- 1. Study size and feasibility

Power calculations are based on the formulas from Chow et al. (2008). The matched patient counts were obtained after applying inclusion-exclusion criteria and propensity score matching. The outcome risk was estimated in the matched cohort unstratified by exposure status, to avoid inferential analyses while assessing feasibility.

#### Table 13. Power and sample size

1. Limitation of the methods

There are several potential limitations with the methods specified in this protocol.

1. The data were not collected for research and some important variables may not be collected or will be measured imperfectly
   1. We have selected validated algorithms when possible
   2. We have created proxies for important variables that are not directly captured in the data to reduce confounding by unmeasured factors
2. There will not be randomization
   1. We have emulated the design of a target trial
   2. We have balanced compared groups on important risk factors for the outcome(s)
3. On treatment follow up may be short in real-world practice, there is potential for informative censoring
   1. The results may not capture efficacy of long-term treatment but can measure effectiveness in populations as they are actually treated.
   2. We will do sensitivity analyses regarding reasons for censoring and incorporating censoring weights
4. Protection of human subjects

The study proposal has been reviewed and approved by the Brigham and Women’s Hospital IRB to ensure ethical treatment of human subjects as well as privacy protections (HIPAA). The proposed study is observational research that makes secondary use of data collected as part of routine care and does not involve any intervention, alteration in standard clinical care or use of any procedure in patients. Therefore, there will be no adverse events related to the study itself. No patients will be contacted for any of the proposed studies. Prior to our acquisition of the data, all personal identifiers will be encrypted. This encryption minimizes the risk of patient reidentification in the unlikely event of a breach in data security. The institution’s uses standard-issue virus protection software and access to data is controlled through the use of individual passwords known only to study staff. Study staff are required to complete the Partners HealthCare System human-subject protection education requirements as well as HIPAA training prior to being allowed to work on any data and are regularly re-certificated. As a further layer of privacy protection, cell sizes less than 11 will be suppressed in tables produced from Medicare data, in accordance with the data use agreement.

1. Reporting of adverse events

The proposed study is observational research that makes secondary use of data collected as part of routine care and does not involve any intervention or alteration in clinical care. Therefore, reporting of adverse events related to this study is not applicable. Safety evaluations for this study are limited to the specified safety outcomes stated in section 4.4.2.

1. References

1. Zinman B, Wanner C, Lachin JM, et al. Empagliflozin, Cardiovascular Outcomes, and Mortality in Type 2 Diabetes. *New England Journal of Medicine*. 2015;373(22):2117-2128. doi:10.1056/NEJMoa1504720

2. Patorno E, Bateman BT, Huybrechts KF, et al. Pregabalin use early in pregnancy and the risk of major congenital malformations. *Neurology*. May 23 2017;88(21):2020-2025. doi:10.1212/WNL.0000000000003959

3. Leon BM, Maddox TM. Diabetes and cardiovascular disease: Epidemiology, biological mechanisms, treatment recommendations and future research. *World J Diabetes*. Oct 10 2015;6(13):1246-58. doi:10.4239/wjd.v6.i13.1246

4. Patorno E, Goldfine AB, Schneeweiss S, et al. Cardiovascular outcomes associated with canagliflozin versus other non-gliflozin antidiabetic drugs: population based cohort study. *Bmj*. Feb 6 2018;360:k119. doi:10.1136/bmj.k119

5. Patorno E, Pawar A, Franklin JM, et al. Empagliflozin and the Risk of Heart Failure Hospitalization in Routine Clinical Care. *Circulation*. Jun 18 2019;139(25):2822-2830. doi:10.1161/CIRCULATIONAHA.118.039177

6. Hatoum IJ, Blackstone R, Hunter TD, et al. Clinical Factors Associated With Remission of Obesity-Related Comorbidities After Bariatric Surgery. *JAMA Surg*. Feb 2016;151(2):130-7. doi:10.1001/jamasurg.2015.3231

7. Krumme AA, Pawar A, Schneeweiss S, et al. Study protocol for the dabigatran, apixaban, rivaroxaban, edoxaban, warfarin comparative effectiveness research study. *Journal of comparative effectiveness research*. Jan 2018;7(1):57-66. doi:10.2217/cer-2017-0053

8. Kiyota Y, Schneeweiss S, Glynn RJ, Cannuscio CC, Avorn J, Solomon DH. Accuracy of Medicare claims-based diagnosis of acute myocardial infarction: estimating positive predictive value on the basis of review of hospital records. *Am Heart J*. Jul 2004;148(1):99-104. doi:10.1016/j.ahj.2004.02.013

9. Wahl PM, Rodgers K, Schneeweiss S, et al. Validation of claims-based diagnostic and procedure codes for cardiovascular and gastrointestinal serious adverse events in a commercially-insured population. *Pharmacoepidemiology and drug safety*. Jun 2010;19(6):596-603. doi:10.1002/pds.1924

10. Tirschwell DL, Longstreth WT, Jr. Validating administrative data in stroke research. *Stroke; a journal of cerebral circulation*. Oct 2002;33(10):2465-70. doi:10.1161/01.str.0000032240.28636.bd

11. Analytics. TH. The Truven Health MarketScan Databases for Health Services Researchers. IBM Watson Health. Accessed Sep 6 2018, <https://truvenhealth.com/portals/0/assets/2017_MarketScan_Databases_Health_Services_Researchers.pdf>

12. ResDAC Knowledgebase. Accessed 12/10/2021, 2021. <https://resdac.org/articles>

13. Insight O. *Clinformatics® Data Mart User Manual Version 8.1*. Vol. 8.1. 2020.

1. Appendices

See excel files.

Appendix A - study population entry criteria (exposure)

Appendix B - drug, diagnosis and procedure based inclusion/exclusion criteria

Appendix C - drug, diagnosis and procedure based covariates

Appendix D - outcome

Appendix E - care setting
