# Supplementary material for: HARmonized Protocol Template to Enhance Reproducibility of hypothesis evaluating real‐world evidence studies on treatment effects: A good practices report of a joint ISPE/ISPOR task force
Source: Pharmacoepidemiol Drug Saf. 2022 Oct 10;32(1):44–55. doi: 10.1002/pds.5507 (PMC9771861; doi:10.1002/pds.5507)
Supplement: Supplementary file 3 — Appendix 3. Example use cases. [file PDS-32-44-s004.zip › Appendix 3/Example 2 Cancer new vs standard of care.docx]

# 1. Title Page

DISCLAIMER: This protocol is based on the research question and design from a real study protocol under development. It has been anonymized due to the ongoing nature of the work. To reflect the realistic situation of a developing protocol with multiple versions after input from various stakeholders, at several points in the document, the protocol states that a decision is “to be discussed” or “to be determined” indicating that it will be further developed in a later version of the protocol. Abbreviated responses are provided to illustrate how to use the protocol template, however appendices were not prepared for this example protocol.

| Title | Drug A/B combination as a second- or third-line treatment of patients with biomarker Y+ metastatic cancer compared to usual standard care |
| --- | --- |
| Research question & Objectives | The objective is to evaluate the comparative effectiveness and cost-effectiveness of Drug A/B as a second- or third-line treatment of patients with biomarker Y+ metastatic colorectal cancer compared to standard care |
| Protocol version | V1 |
| Last update date | 2021-10-26 |
| Contributors | **Primary investigator contact information:**  anonymous@anonymous.org  **Contributor names:**  John Smith  Jane Doe |
| Study registration | **Site:** Health Technology Assessment of Anonymous Country  **Identifier:** To Be Determined |
| Sponsor | **Organization:** Health Technology Assessment of Anonymous Country  **Contact:** anonymous@anonymous.org |
| Conflict of interest | n/a |

Table of contents

[1. Title Page 1](#_Toc102631400)

[2. Abstract 3](#_Toc102631401)

[3. Amendments and updates 4](#_Toc102631402)

[4. Milestones 5](#_Toc102631403)

[Table 1 Milestones 5](#_Toc102631404)

[5. Rationale and background 5](#_Toc102631405)

[6. Research question and objectives 6](#_Toc102631406)

[Table 2 Primary and secondary objectives and research questions 6](#_Toc102631407)

[7. Research methods 10](#_Toc102631408)

[7.1. Study design 10](#_Toc102631409)

[7.2. Study design diagram 11](#_Toc102631410)

[7.3. Setting 11](#_Toc102631411)

[7.3.1 Context and rationale for definition of time 0 (and other primary time anchors) for entry to the study population 11](#_Toc102631412)

[Table 3 Operational Definition of Time 0 (index date) and other primary time anchors 11](#_Toc102631413)

[7.3.2 Context and rationale for study inclusion criteria: 12](#_Toc102631414)

[Table 4. Operational Definitions of Inclusion Criteria 12](#_Toc102631415)

[7.3.3 Context and rationale for study exclusion criteria 13](#_Toc102631416)

[Table 5. Operational Definitions of Exclusion Criteria 13](#_Toc102631417)

[7.4. Variables 14](#_Toc102631418)

[7.4.1 Context and rationale for exposure(s) of interest 14](#_Toc102631419)

[Table 6. Operational Definitions of Exposure 14](#_Toc102631420)

[7.4.2 Context and rationale for outcome(s) of interest 15](#_Toc102631421)

[Table 7. Operational Definitions of Outcome 15](#_Toc102631422)

[7.4.3 Context and rationale for follow up 16](#_Toc102631423)

[Table 8. Operational Definitions of Follow Up 17](#_Toc102631424)

[7.4.4 Context and rationale for covariates (confounding variables and effect modifiers, e.g. risk factors, comorbidities, comedications) 17](#_Toc102631425)

[Table 9. Operational Definitions of Covariates 18](#_Toc102631426)

[7.5. Data analysis 18](#_Toc102631427)

[7.5.1 Context and rationale for analysis plan 18](#_Toc102631428)

[Table 10. Primary, secondary, and subgroup analysis specification 18](#_Toc102631429)

[Table 11. Sensitivity analyses – rationale, strengths and limitations 20](#_Toc102631430)

[7.6. Data sources 20](#_Toc102631431)

[7.6.1 Context and rationale for data sources 20](#_Toc102631432)

[Table 12. Metadata about data sources and software 21](#_Toc102631433)

[7.7. Data management 21](#_Toc102631434)

[7.8. Quality control 22](#_Toc102631435)

[7.9. Study size and feasibility 22](#_Toc102631436)

[Table 13. Power and sample size 23](#_Toc102631437)

[8. Limitation of the methods 23](#_Toc102631438)

[9. Protection of human subjects 24](#_Toc102631439)

[10. Reporting of adverse events 24](#_Toc102631440)

[11. References 24](#_Toc102631441)

[12. Appendices 24](#_Toc102631442)

2. Abstract

Metastatic [organ]cancer with a biomarker Y+ mutation is a rare type of cancer. It is associated with a poorer prognosis and has a greater risk of recurring than [organ]cancer without the mutation. There has been little improvement in survival for biomarker Y mutation-positive cancer despite improvements for this type of cancer in general. At the moment there are currently no effective treatments for this type of [organ]cancer. Drug A in combination with Drug B may represent a step change in treatment.

Drug A has a marketing authorisation in combination with Drug B 'for the treatment of adult patients with metastatic [organ]cancer with a biomarker Y+ mutation, who have received prior systemic therapy. There are a number of gaps identified in terms of the effectiveness and cost-effectiveness of Drug A/B combination in clinical practice compared to standard of care (SoC). Specifically:

- To what extent are the patients who are treated with Drug A/B in clinical practice similar to the patients included in the pivotal trial ACRONYM RCT?
- Do we know whether the relative effectiveness of Drug A/B compared to SoC in clinical practice is similar to relative effectiveness measured in the ACRONYM trial?
- It is unknown to which extent Drug A/B affects the quality of life of patients measured by generic (EQ-5D) and disease-specific (EORTC QLQ-CRXX and EORTC QLQ-CXY) compared to SoC
- There is no information on the cost-effectiveness of Drug A/B compared to SoC based on data from clinical practice.

This study aims to address the gaps in knowledge in [anonymous country]. The outcomes of the study may influence the guidance for use and reimbursement of Drug A/B in the [anonymous country] clinical practice.

The study will use the XYZ Data Registry in [anonymous country]. The registry includes longitudinal electronic health records, insurance claims, death records, and patient reported outcomes for a national registry of patients with metastatic [organ] carcinoma. The data in the registry was specifically collected to address the research objectives of this protocol. The quality of the registry has been assessed and found to be high using the REQUEST tool, and detailed documentation about the data sources, linkage, provenance, quality assurance, and financing (www.redactedlink.com).

3. Amendments and updates

| **Version date** | **Version number** | **Section of protocol** | **Amendment or update** | **Reason** |
| --- | --- | --- | --- | --- |
| 2021-10-26 | 1 | All | Preparation Research Protocol | Draft in preparation |
|  |  |  |  |  |
|  |  |  |  |  |
|  |  |  |  |  |

1. Milestones

#### Table 1 Milestones

| **Milestone** | **Date** |
| --- | --- |
| Feasibility counts | 2022-01-01 |
| Draft 1 of protocol complete | 2022-03-01 |
| Registration of protocol and lock/freeze data | 2022-03-01 |
| Study progress report 1 | 2022-09-01 |
| Study progress report 2 (interim analysis) | 2023-03-01 |
| Final report of study results | 2023-09-01 |

1. Rationale and background

**What is known about the condition:** Metastatic [organ]cancer with a biomarker Y+ mutation is a rare type of cancer. It is associated with a poorer prognosis and has a greater risk of recurring than [organ]cancer without the mutation. There has been little improvement in survival for biomarker Y mutation-positive cancer despite overall improvements in survival for this type of cancer suggesting an unmet medical need. At the moment there are currently no effective treatments for this type of [organ]cancer. Drug A in combination with Drug B may represent a step change in treatment.

**What is known about the exposure of interest:** Drug A has a marketing authorisation in combination with Drug B ‘for the treatment of adult patients with metastatic [organ]cancer with a biomarker Y+ mutation, who have received prior systemic therapy' based on a pivotal clinical trial ACRONYM RCT.

**Gaps in knowledge:** There are number of gaps identified in terms of the effectiveness and cost-effectiveness of Drug A/B combination in clinical practice compared to standard of care (SoC):

- To which extent are the patients who are treated with Drug A/B in clinical practice similar to the patients included in the pivotal trial ACRONYM RCT?
- Do we know whether the relative effectiveness of Drug A/B compared to SoC in clinical practice is similar to relative effectiveness measured in the ACRONYM RCT trial?
- It is unknown to which extent Drug A/B affects the quality of life of patients measured by generic (EQ-5D) and disease-specific (EORTC QLQ-CRXXand EORTC QLQ-CXY) compared to SoC
- There is no information on the cost-effectiveness of Drug A/B compared to SoC based on data from clinical practice.

**What is the expected contribution of this study?** This study is aiming to address the gaps in knowledge in [anonymous country]. The outcomes of the study may influence the guidance for coverage and reimbursement of Drug A/B in the [anonymous country] clinical practice.

1. Research question and objectives

#### Table 2 Primary and secondary objectives and research questions

1. **Primary objective and research question**

| **Objective:** | To compare overall survival (OS) in patients > 18 years with metastatic [organ]carcinoma with a biomarker Y+ mutation who have shown progression after first line treatment and have indicated informed consent for longitudinal observational data collection as part of the XYZ Registry and are treated with a combination of Drug A and Drug B compared to controls who are treated with standard care for a period of a maximum of 2 years (or 4 years). Comparator patients will be included retrospectively as well prospectively. |
| --- | --- |
| **Hypothesis:** | Overall survival will be improved with Drug A and Drug B compared to standard of care. |
| **Population *(mention key inclusion-exclusion criteria):*** | Patients with biomarker Y+ positive metastatic [organ] cancer who progressed after at least of one line of treatment |
| **Exposure:** | Initiation of Drug A in combination with Drug B |
| **Comparator:** | Standard of care. In [anonymous country], SoC second line therapy is Drug C or in third line the SoC is Drug D. There will be 2 comparator arms. One will be initiation of Drug C or Drug D using concurrent years of data with the exposure arm. The other comparator group will be initiatiors of Drug C or Drug D using historical controls from years of data prior to availability of Drug A/B. |
| **Outcome:** | Overall Survival (OS) |
| **Time *(when follow up begins and ends):*** | Follow up from day after initiation of therapy until the first of outcome, discontinuation, add/switch therapy, disenrollment, end of study period, nursing home admission, death, progression of therapy, others to be discussed. |
| **Setting:** | Outpatient care |
| **Main measure of effect:** | Hazard Ratio (to be discussed). |

1. **Secondary objective 1 and research question**

| **Objective:** | To compare progression-free survival (PFS) and/or time to treatment failure (TTTF) in patients > 18 years with metastatic [organ] carcinoma with a biomarker Y+ mutation who have shown progression after first line treatment and have indicated informed consent for longitudinal observational data collection as part of the XYZ Registry and are treated with a combination of Drug A/B compared to patients who are treated with standard care for a period of a maximum of 2 years (or 4 years). Comparator patients will be included retrospectively as well prospectively |
| --- | --- |
| **Hypothesis:** | Progression-free survival (PFS) and/or time to treatment failure (TTTF) will be improved with Drug A and Drug B compared to standard of care. |
| **Population *(mention key inclusion-exclusion criteria):*** | Patients with biomarker Y+ positive metastatic [organ] cancer who progressed after at least of one line of treatment |
| **Exposure:** | Initiation of Drug A in combination with Drug B |
| **Comparator:** | Standard of care. In [anonymous country], SoC second line therapy is Drug C or in third line the SoC is Drug D. There will be 2 comparator arms. One will be initiation of Drug C or Drug D using concurrent years of data with the exposure arm. The other comparator group will be initiatiors of Drug C or Drug D using historical controls from years of data prior to availability of Drug A/B. |
| **Outcome:** | Progression-free survival (PFS) and/or time to treatment failure (TTTF) |
| **Time *(when follow up begins and ends):*** | Follow up from day after initiation of therapy until the first of outcome, discontinuation, add/switch therapy, disenrollment, end of study period, nursing home admission, death, progression of therapy, others to be discussed. |
| **Setting:** | Outpatient care |
| **Main measure of effect:** | Hazard Ratio (to be discussed). |

1. **Secondary objective 3 and research question**

| **Objective:** | To compare generic health-related quality of life (HRQoL) (using EQ-5D) and disease-specific HRQoL (using EORTC-QLQ-C30) in patients > 18 years with metastatic [organ]carcinoma with a biomarker Y+ mutation who have shown progression after first line treatment and have indicated informed consent for longitudinal observational data collection as part of the XYZ Registry and are treated with a combination of Drug A/B compared to patients who are treated with standard care for a period of a maximum of 2 years (or 4 years). Comparator patients will be included retrospectively as well prospectively. |
| --- | --- |
| **Hypothesis:** | Generic health-related quality of life (HRQoL) (using EQ-5D) and disease-specific HRQoL (using EORTC-QLQ-C30) will be improved with Drug A and Drug B compared to standard of care. |
| **Population *(mention key inclusion-exclusion criteria):*** | Patients with biomarker Y+ positive metastatic [organ] cancer who progressed after at least of one line of treatment |
| **Exposure:** | Initiation of Drug A in combination with Drug B |
| **Comparator:** | Standard of care. In [anonymous country], SoC second line therapy is Drug C or in third line the SoC is Drug D. There will be 2 comparator arms. One will be initiation of Drug C or Drug D using concurrent years of data with the exposure arm. The other comparator group will be initiatiors of Drug C or Drug D using historical controls from years of data prior to availability of Drug A/B. |
| **Outcome:** | Generic health-related quality of life (HRQoL) (using EQ-5D) and disease-specific HRQoL (using EORTC-QLQ-C30) |
| **Time:** | Follow up from day after initiation of therapy until the first of outcome, discontinuation, add/switch therapy, disenrollment, end of study period, nursing home admission, death, progression of therapy, others to be discussed. |
| **Setting:** | Outpatient care |
| **Main measure of effect:** | (to be discussed) |

1. **Secondary objective 3 and research question**

| **Objective:** | To compare toxicity and/or adverse effects in patients > 18 years with metastatic [organ]carcinoma with a biomarker Y+ mutation who have shown progression after first line treatment and have indicated informed consent for longitudinal observational data collection as part of the XYZ Data Registry and are treated with a combination of Drug A/B compared to patients who are treated with standard care for a period of a maximum of 2 years (or 4 years). Comparator patients will be included retrospectively as well prospectively. |
| --- | --- |
| **Hypothesis:** | Toxicity and/or adverse effects will be no different with Drug A and Drug B compared to standard of care. |
| **Population *(mention key inclusion-exclusion criteria):*** | Patients with biomarker Y+ positive metastatic [organ] cancer who progressed after at least of one line of treatment |
| **Exposure:** | Initiation of Drug A in combination with Drug B |
| **Comparator:** | Standard of care. In [anonymous country], SoC second line therapy is Drug C or in third line the SoC is Drug D. There will be 2 comparator arms. One will be initiation of Drug C or Drug D using concurrent years of data with the exposure arm. The other comparator group will be initiatiors of Drug C or Drug D using historical controls from years of data prior to availability of Drug A/B. |
| **Outcome:** | Toxicity and/or adverse effects |
| **Time:** | Follow up from day after initiation of therapy until the first of outcome, discontinuation, add/switch therapy, disenrollment, end of study period, nursing home admission, death, progression of therapy, others to be discussed. |
| **Setting:** | Outpatient care |
| **Main measure of effect:** | Hazard ratio (to be discussed) |

1. **Secondary objective 4 and research question**

| **Objective:** | To compare resource use in patients > 18 years with metastatic [organ]carcinoma with a biomarker Y+ mutation who have shown progression after first line treatment and have indicated informed consent for longitudinal observational data collection as part of the XYZ Data Registry and are treated with a combination of Drug A/B compared to patients who are treated with standard care for a period of a maximum of 2 years (or 4 years). Comparator patients will be included retrospectively as well prospectively |
| --- | --- |
| **Hypothesis:** | Resource utilization will be reduced with Drug A and Drug B compared to standard of care. |
| **Population *(mention key inclusion-exclusion criteria):*** | Patients with biomarker Y+ positive metastatic [organ] cancer who progressed after at least of one line of treatment |
| **Exposure:** | Initiation of Drug A in combination with Drug B |
| **Comparator:** | Standard of care. In [anonymous country], SoC second line therapy is Drug C or in third line the SoC is Drug D. There will be 2 comparator arms. One will be initiation of Drug C or Drug D using concurrent years of data with the exposure arm. The other comparator group will be initiatiors of Drug C or Drug D using historical controls from years of data prior to availability of Drug A/B. |
| **Outcome:** | Resource utilization |
| **Time:** | Follow up from day after initiation of therapy until the first of outcome, discontinuation, add/switch therapy, disenrollment, end of study period, nursing home admission, death, progression of therapy, others to be discussed. |
| **Setting:** | Outpatient care |
| **Main measure of effect:** | Descriptive statistics (to be discussed) |

1. Research methods
   1. Study design

**Research design (e.g. cohort, case-control, etc.):** New user active comparator cohort study

**Rationale for study design choice:** In order compare the new treatment (Drug A/B) with standard of care (SoC), patients need to be followed from start treatment until censoring (death, progression, treatment failure, end of follow-up). Use of this design will help prevent time related biases such as depletion of susceptible, immortal time bias and adjustment for causal intermediates.

- 1. Study design diagram


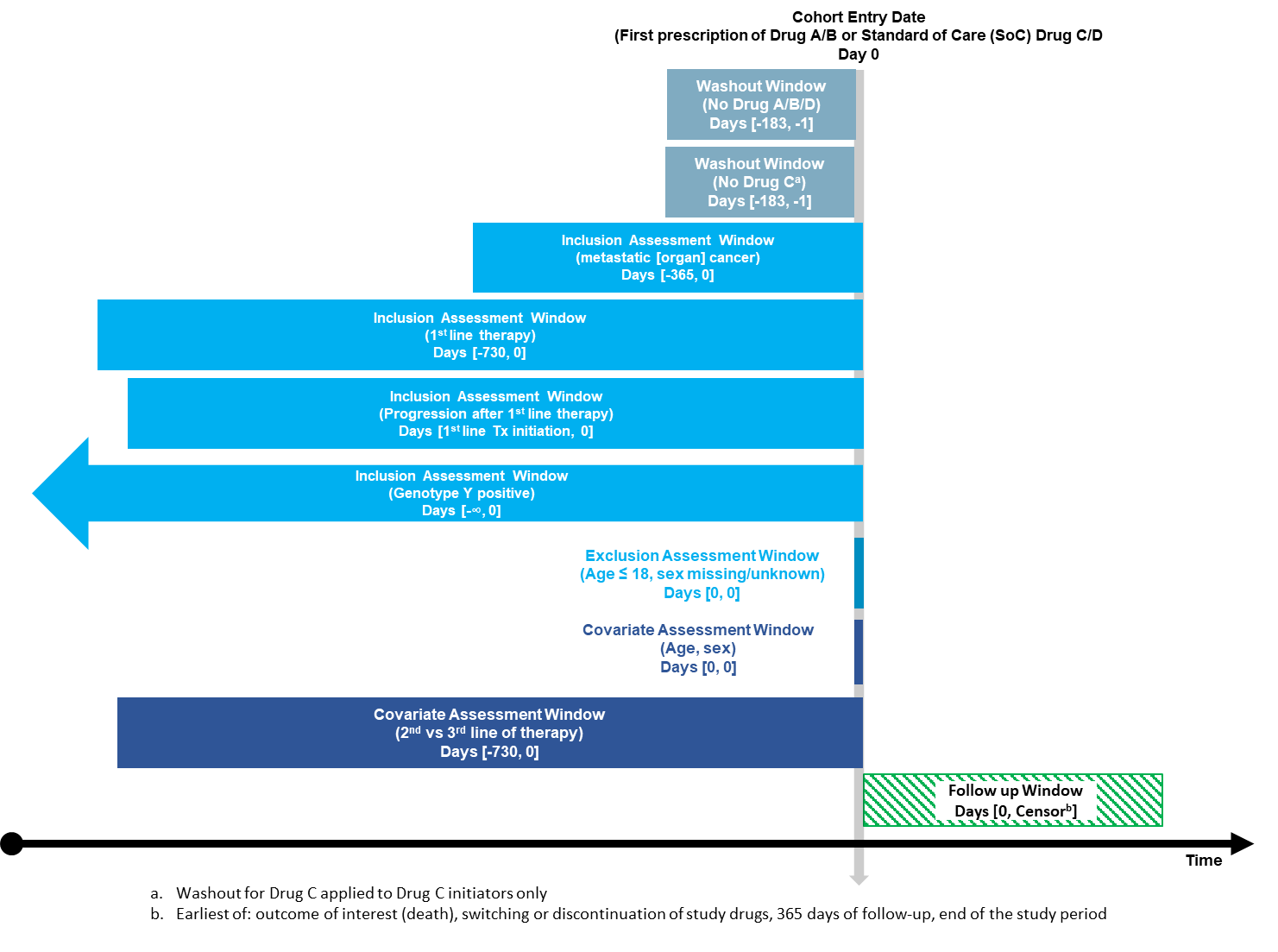


- 1. Setting

#### 7.3.1 Context and rationale for definition of time 0 (and other primary time anchors) for entry to the study population

Time 0 will be the day of initiation of Drug A/B combination therapy. Only patients who initiate Drug A and B on the same day will be included. A washout covering all available data will be applied to ensure that there is no prior use of either Drug A or Drug B by themselves or in combination prior to time 0.

#### Table 3 Operational Definition of Time 0 (index date) and other primary time anchors

| **Study population name(s)** | **Time Anchor Description**  **(e.g. time 0)** | **Number of entries** | **Type of entry** | **Washout window** | **Care Setting^1^** | **Code Type^2^** | **Diagnosis position** | **Incident with respect to…** | **Measurement characteristics/**  **validation** | **Source of algorithm** |
| --- | --- | --- | --- | --- | --- | --- | --- | --- | --- | --- |
| **Exposure**  Drug A/B | Date of incident dispensation of Drug A/B (time 0) | Single | Incident | [-∞, 0] | n/a | ATCC | n/a | Drug A or Drug B alone, Drug A/B in combination, Drug D | No validation study | Investigator review of drug codes  XYZ registry dispensing data years 2021-2022 |
| **Comparator 1**  (concurrent)  Standard of Care (SoC).  Drug C or Drug D | Date of incident dispensation of standard care  (time 0) | Single | Incident | [-∞, 0] | n/a | ATCC | n/a | Drug C initiators must be incident with respect to Drug A, B, C and D  Drug D initiators must be incident with respect to Drug A, B, D but not C. | No validation study | Investigator review of drug codes  XYZ registry dispensing data years 2021-2022 |
| **Comparator 2**  (historical)  Standard of Care (SoC).  Drug C or Drug D | Date of incident dispensation of standard care (time 0) | Single | Incident | [-∞, 0] | n/a | ATCC | n/a | Drug C initiators must be incident with respect to Drug A, B, C and D  Drug D initiators must be incident with respect to Drug A, B, D but not C. | No validation study | Investigator review of drug codes  XYZ registry dispensing data years 2019-2020 |

^1^ IP = inpatient, OP = outpatient, ED = emergency department, OT = other, n/a = not applicable

^2^See appendix for listing of clinical codes for each study parameter

#### 7.3.2 Context and rationale for study inclusion criteria:

We included patients 18 or older with metastatic [organ] cancer who were biomarker Y+ and progressed within 2 years after 1^st^ line therapy.

#### Table 4. Operational Definitions of Inclusion Criteria

| **Criterion** | **Details** | **Order of application** | **Assessment window** | **Care Settings¹** | **Code Type^2^** | **Diagnosis position^3^** | **Applied to study populations:** | **Measurement characteristics/**  **validation** | **Source for algorithm** |
| --- | --- | --- | --- | --- | --- | --- | --- | --- | --- |
| Age ≥18 yrs |  | Before selection of index date | [0, 0] | n/a | n/a | n/a | Exposure  Comparator 1 Comparator 2 |  | n/a |
| Metastatic [organ] cancer |  | Before selection of index date | [365, 0] | Any | ICD10 | To be discussed | Exposure  Comparator 1 Comparator 2 | No validation study | Clinician defined algorithm for XYZ registry |
| Biomarker Y positive cancer |  | Before selection of index date | [-∞, 0] | Any | n/a | To be discussed | Exposure  Comparator 1 Comparator 2 | Unknown | Genomic testing |
| 1^st^ line therapy (Drug E) | Patients must have previously received 1^st^ line therapy | Before selection of index date | [-730, 0] | Any | n/a | n/a | Exposure  Comparator 1 Comparator 2 | No validation study | Investigator review of drug codes  XYZ registry dispensing data years 2021-2022 or 2019-2020 |
| Progression after first line treatment | Progression of tumor will be defined by a composite measure – imaging results or absence of toxic adverse events during 1^st^ line therapy up until initiation of 2^nd^ line therapy | Before selection of index date | [Start of 1^st^ line tx, 0] | Any | ICD10 + other | To be discussed | Exposure  Comparator 1 Comparator 2 | No validation study | Investigator defined composite measure |

^1^ IP = inpatient, OP = outpatient, ED = emergency department, OT = other, n/a = not applicable

^2^ See appendix for listing of clinical codes for each study parameter

^3^ Specify whether a diagnosis code is required to be in the primary position (main reason for encounter)

#### 7.3.3 Context and rationale for study exclusion criteria

We exclude patients with missing/unknown age or sex. Other exclusion criteria are to be discussed.

#### Table 5. Operational Definitions of Exclusion Criteria

| **Criterion** | **Details** | **Order of application** | **Assessment window** | **Care Settings¹** | **Code Type^2^** | **Diagnosis position^3^** | **Applied to study populations:** | **Measurement characteristics/**  **validation** | **Source for algorithm** |
| --- | --- | --- | --- | --- | --- | --- | --- | --- | --- |
| Age missing |  | Before selection of index date | [0, 0] | n/a | n/a | n/a | Exposure  Comparator 1 Comparator 2 |  | n/a |
| Sex missing/unknown |  | Before selection of index date | [0, 0] | n/a | n/a | n/a | Exposure  Comparator 1 Comparator 2 |  | n/a |
| To be discussed |  | Before selection of index date | *To be discussed* | *n/a* | *n/a* | *n/a* | Exposure  Comparator 1 Comparator 2 |  | *n/a* |

^1^ IP = inpatient, OP = outpatient, ED = emergency department, OT = other, n/a = not applicable

^2^ See appendix for listing of clinical codes for each study parameter

^3^ Specify whether a diagnosis code is required to be in the primary position (main reason for encounter)

- 1. Variables

#### 7.4.1 Context and rationale for exposure(s) of interest

Exposure to Drug A/B and standard of care comparators will be defined using ATCC codes. Drug A/B combination is approved for 2^nd^ or 3^rd^ line therapy for metastatic [organ] cancer for biomarker Y+ cancers. Drugs C and D are approved as standard of care for 2^nd^ and 3^rd^ line therapies, respectively.

**Algorithm to define duration of exposure effect:**

If a refill occurs before the end of days supply dispensed, add overlapping days to the end of the subsequent dispensing’s day supply. Assume that the effect of a pill lasts for 30 days. Therefore, we allow up to a 30 day gap between a dispensation + days supply and refill. We also add 30 days to the last dispensation + days supply in a treatment episode and consider this “exposed” time.

#### Table 6. Operational Definitions of Exposure

| **Exposure group name(s)** | **Detail** | **Washout window** | **Assessment Window** | **Care Setting^1^** | **Code Type^2^** | **Diagnosis position^3^** | **Applied to study populations:** | **Incident with respect to…** | **Measurement characteristics/**  **validation** | **Source of algorithm** |
| --- | --- | --- | --- | --- | --- | --- | --- | --- | --- | --- |
| Exposure: Drug A/B |  | [-∞, 0] | [1, censor] | Hospital | ATCC | n/a | Exposure  Comparator 1 Comparator 2 | Drug A or Drug B alone, Drug A/B in combination, Drug D | No validation study | Investigator review of drug codes  XYZ registry dispensing data years 2021-2022 |
| Comparator 1: SoC  (concurrent) |  | [-∞, 0] | [1, censor] | Hospital, palliative care | ATCC | n/a | Exposure  Comparator 1 Comparator 2 | Drug C initiators must be incident with respect to Drug A, B, C and D  Drug D initiators must be incident with respect to Drug A, B, D but not C. | No validation study | Investigator review of drug codes  XYZ registry dispensing data years 2021-2022 |
| Comparator 2: SoC  (historical) |  | [-∞, 0] | [1, censor] | Hospital, palliative care | ATCC | n/a | Exposure  Comparator 1 Comparator 2 | Drug C initiators must be incident with respect to Drug A, B, C and D  Drug D initiators must be incident with respect to Drug A, B, D but not C. | No validation study | Investigator review of drug codes  XYZ registry dispensing data years 2019-2020 |

^1^ IP = inpatient, OP = outpatient, ED = emergency department, OT = other, n/a = not applicable

^2^ See appendix for listing of clinical codes for each study parameter

^3^ Specify whether a diagnosis code is required to be in the primary position (main reason for encounter)

#### 7.4.2 Context and rationale for outcome(s) of interest

Overall survival is the primary outcome of interest. This is a hard outcome that is of great interest to patients, clinicians and policy decision makers. We will explore secondary outcomes such as progression free survival, health related quality of life, toxic adverse effects, and healthcare resource utilization.

#### Table 7. Operational Definitions of Outcome

| **Outcome name** | **Details** | **Primary outcome?** | **Type of outcome** | **Washout window** | **Care Settings¹** | **Code Type^2^** | **Diagnosis Position^3^** | **Applied to study populations:** | **Outcome measurement characteristics/**  **validation** | **Source of algorithm** |
| --- | --- | --- | --- | --- | --- | --- | --- | --- | --- | --- |
| Overall Survival |  | Yes | Time-to-event | n/a | n/a | n/a | n/a | Exposure, comparator 1, comparator 2 | The positive predictive value of death in XYZ Registry is 96%. Sensitivity is 93%. | Hospital records + registry validation |
| Progression-free survival |  | No | Time-to-event | n/a | n/a | n/a | n/a | Exposure, comparator 1, comparator 2 | Unknown | (still in discussion) Derive variable from hospital pharmacy data + lab/report/imaging + toxic side effects + time to treatment failure |
| HRQoL (generic) |  | No | Continuous, categorical, binary | n/a | n/a | n/a | n/a | Exposure, comparator 1, comparator 2 | EQ-5D-5L is a validated instrument widely used in oncology (Schwenkglenks 2016 Expert Review of Pharmacoeconomics and Outcomes Research) | EQ-5D-5L |
| HRQoL (disease-specific) |  | No | Continuous, categorical, binary | n/a | n/a | n/a | n/a | Exposure, comparator 1, comparator 2 | EORTC-CXX and EORTC-CXY are validated widely used instruments (John Smith 2021, European Journal of Cancer) | EORTC-CXX and EORTC-CXY |
| Toxic side effects |  | No | Time-to-event | n/a | n/a | n/a | To be discussed | Exposure, comparator 1, comparator 2 | To be discussed | To be discussed |
| Resource use |  | No | To be discussed | n/a | n/a | n/a | n/a | Exposure, comparator 1, comparator 2 | To be discussed | To be discussed |

^1^ IP = inpatient, OP = outpatient, ED = emergency department, OT = other, n/a = not applicable

^2^ See appendix for listing of clinical codes for each study parameter

^3^ Specify whether a diagnosis code is required to be in the primary position (main reason for encounter)

#### 7.4.3 Context and rationale for follow up

For the primary outcome of death and secondary outcomes of progression free survival and toxic adverse side effects, we will implement an on-treatment follow up analysis with censoring as defined in table 6. For health-related quality of life outcomes, we will use panel measurements in accordance with the XYZ registry’s data collection schedule. Details of the analyses for health-related quality of life outcomes will be determined after further discussion.

#### Table 8. Operational Definitions of Follow Up

|  |  |  |  |
| --- | --- | --- | --- |
| **Follow up start** | Day 1 |  |  |
| **Follow up end^1^** | **Select all that apply** |  | **Specify** |
| **Date of outcome** | Yes |  | See Table 5 |
| **Date of death** | Yes |  | Discharged dead or registry recorded death, whichever came 1st |
| **End of observation in data** | Yes |  | Allow 30 day gaps in enrolment |
| **Day X following index date**  *(specify day)* | Yes |  | Day 365 |
| **End of study period**  (specify date) | Yes |  | 30-Sep-15 |
| **End of exposure**  *(specify operational details,*  *e.g. stockpiling algorithm, grace period)* | Yes |  | **Stockpiling algorithm:** If refill occurs before end of days supply, count overlapping days at the end of the subsequent dispensing’s day supply.  **Grace period:** Bridge gaps of ≤30 days between dispensation + days supply and refill. Add 30 days to last dispensation + days supply in a treatment episode. |
| **Date of add to/switch from exposure**  *(specify algorithm)* | Yes |  | Date that patient in exposed group is dispensed comparator drug or vice versa |
| **Other date** *(specify)* | Yes |  | Nursing home admission |

^1^ Follow up ends at the first occurrence of any of the selected criteria that end follow up.

#### 7.4.4 Context and rationale for covariates (confounding variables and effect modifiers, e.g. risk factors, comorbidities, comedications)

We will adjust for major confounders such as age and sex. In the primary analysis, we will balance the Drug A/B exposed group (2^nd^ or 3^rd^ line) and Drug C (2^nd^ line) or D (3^rd^ line) SoC comparator group on prior exposure to Drug C, which will be an indicator of line of therapy. Patients who initiate Drug A/B and have prior exposure to Drug C are assumed to be 3rd line therapy. Those without prior exposure to Drug C are assumed to be on 2^nd^ line therapy. SoC initiators of Drug C (2nd line) will not have prior exposure because of the washout imposed to define new initiation and will be considered on 2^nd^ line therapy, in accordance with guidelines for use. SoC initiators of Drug D will be considered on 3rd line therapy regardless of whether they had prior exposure to Drug C (2^nd^ line therapy). Other covariates are to be determined after additional discussion.

#### Table 9. Operational Definitions of Covariates

| **Characteristic** | **Details** | **Type of variable** | **Assessment window** | **Care Settings¹** | **Code Type^2^** | **Diagnosis Position^3^** | **Applied to study populations:** | **Measurement characteristics/validation** | **Source for algorithm** |
| --- | --- | --- | --- | --- | --- | --- | --- | --- | --- |
| Age | (cohort entry year - year of birth) | Continuous | [0,0] | n/a | n/a | n/a | Exposure, comparator 1, comparator 2 | n/a | n/a |
| Sex | Male, Female | Categorical | [0, 0] | n/a | n/a | n/a | Exposure, comparator 1, comparator 2 | n/a | n/a |
| 2^nd^ vs 3^rd^ line therapy | If Drug A/B initiator and prior exposure to Drug C 🡪 3^rd^ line, otherwise 2^nd^ line  If Drug C initiator 🡪 2^nd^ line  If Drug D initiator 🡪 3^rd^ line | Binary | [-∞, 0] | *n/a* | *n/a* | *n/a* | Exposure, comparator 1, comparator 2 | No validation study | n/a |
| To be added |  |  |  |  |  |  |  |  |  |

^1^ IP = inpatient, OP = outpatient, ED = emergency department, OT = other, n/a = not applicable

^2^ See appendix for listing of clinical codes for each study parameter

^3^ Specify whether a diagnosis code is required to be in the primary position (main reason for encounter)

- 1. Data analysis

#### 7.5.1 Context and rationale for analysis plan

We conduct propensity score matched analysis with Cox proportional hazards models for time-to-event outcomes (mortality, progression free survival, toxic adverse effects). Patients with missing age/sex are excluded. Missing values on health-related quality of life at baseline will be imputed using multiple imputation.

The models for analysing health related quality of life and resource use are to be discussed and developed in a later version of this protocol.

#### Table 10. Primary, secondary, and subgroup analysis specification

1. **Primary analysis**

| **Hypothesis:** | Exposure to Drug A/B increases risk of death relative to SoC comparators with the lower bound of the 95% confidence interval for the hazard ratio above 1.0. |
| --- | --- |
| **Exposure contrast:** | Exposure, comparator 1 (concurrent) |
| **Outcome:** | Death |
| **Analytic software:** | SAS 9.4: PHREG, PROC LOGISTIC, Pharmacoepi Toolbox nearest neighbor matching macro (http://www.drugepi.org/dope-downloads/) |
| **Model(s):**  ***(provide details or code)*** | *Outcome model:* Cox proportional hazards  followuptime*status(0) = exposure  *Propensity score model*: logistic regression  Exposure = Risk factors to be determined |
| **Confounding adjustment method** | ***Name method and provide relevant details, e.g. bivariate, multivariable, propensity score matching (specify matching algorithm ratio and caliper), propensity score weighting (specify weight formula, trimming, truncation), propensity score stratification (specify strata definition), other.*** |
|  | We will use logistic regression to estimate a propensity score using covariates that will be determined and recorded in an updated protocol before conducting inferential analysis. We will use this propensity score to nearest-neighbor match with a caliper of 0.2 standard deviations on the logit scale of the propensity score using the nearest neighbor algorithm from the Pharmacoepi Toolbox. |
| **Missing data methods** | ***Name method and provide relevant details, e.g. missing indicators, complete case, last value carried forward, multiple imputation (specify model/variables), other.*** |
|  | Patients with missing or unknown sex will be excluded. Multiple imputation will be used to impute missing data on health related quality of life at baseline. We assume that if no relevant claims diagnoses/procedures are present in the registry data, that the patient does not have the condition/procedure. |
| **Subgroup Analyses** | ***List all subgroups*** |
|  | 1. 2^nd^ vs 3^rd^ line of therapy |

1. **Secondary Analysis 1**

| **Hypothesis:** | Exposure to Drug A/B increases risk of progression free survival relative to SoC comparators with the lower bound of the 95% confidence interval for the hazard ratio above 1.0. |
| --- | --- |
| **Exposure contrast:** | Exposure, comparator 1 (concurrent) |
| **Outcome:** | Progression free survival |
| **Analytic software:** | SAS 9.4: PHREG, PROC LOGISTIC, Pharmacoepi Toolbox nearest neighbor matching macro (http://www.drugepi.org/dope-downloads/) |
| **Model(s):**  ***(provide details or code)*** | *Outcome model:* Cox proportional hazards  followuptime*status(0) = exposure  *Propensity score model*: logistic regression  Exposure = Risk factors to be determined |
| **Confounding adjustment method** | ***Name method and provide relevant details, e.g. bivariate, multivariable, propensity score matching (specify matching algorithm ratio and caliper), propensity score weighting (specify weight formula, trimming, truncation), propensity score stratification (specify strata definition), other.*** |
|  | We will use logistic regression to estimate a propensity score using covariates that will be determined and recorded in an updated protocol before conducting inferential analysis. We will use this propensity score to nearest-neighbor match with a caliper of 0.2 standard deviations on the logit scale of the propensity score using the nearest neighbor algorithm from the Pharmacoepi Toolbox. |
| **Missing data methods** | ***Name method and provide relevant details, e.g. missing indicators, complete case, last value carried forward, multiple imputation (specify model/variables), other.*** |
|  | Patients with missing or unknown sex will be excluded. Multiple imputation will be used to impute missing data on health related quality of life at baseline. We assume that if no relevant claims diagnoses/procedures are present in the registry data, that the patient does not have the condition/procedure. |
| **Subgroup Analyses** | ***List all subgroups*** |
|  | 1. 2^nd^ vs 3^rd^ line of therapy |

#### Table 11. Sensitivity analyses – rationale, strengths and limitations

|  | **What is being varied? How?** | **Why?  (What do you expect to learn?)** | **Strengths of the sensitivity analysis compared to the primary** | **Limitations of the sensitivity analysis compared to the primary** |
| --- | --- | --- | --- | --- |
| Sensitivity Analysis 1 | To be discussed | To be discussed | To be discussed | To be discussed |

- 1. Data sources

#### 7.6.1 Context and rationale for data sources

**Reason for selection:** We are using the XYZ Data Registry in [anonymous country] because this data was specifically collected to address the research objectives of this protocol.

**Strengths of data source(s):** This data will include longitudinal electronic health records, insurance claims, death records, and patient reported outcomes for a national registry of patients with metastatic [organ] carcinoma.

**Limitations of data source(s):** Free-text notes are available but require further NLP processing to extract some relevant characteristics. Missing data methods will be necessary to deal with laboratory results, some of which have a high proportion of missingness.

**Data source provenance/curation:** The registry has detailed documentation about the data sources, linkage, provenance, quality assurance, and financing (www.redactedlink.com).

#### Table 12. Metadata about data sources and software

|  | **Data 1** | **Data 2** |
| --- | --- | --- |
| **Data Source(s):** | XYZ Data Registry (concurrent/prospective) | XYZ Data Registry (historical) |
| **Study Period:** | Jan 1, 2021- Dec 31, 2022 | Jan 1, 2019- Dec 31, 2020 |
| **Eligible Cohort Entry Period:** | Jan 1, 2021- Dec 31, 2022 | Jan 1, 2019- Dec 31, 2020 |
| **Data Version (or date of last update):** | V2.0 – V5.0 | V1.0 |
| **Data sampling/extraction criteria:** | All patients > 18 years with metastatic [organ] carcinoma with a biomarker Y+ mutation who have shown progression after first line treatment and are treated with a combination of Drug A/B or with standard care | All patients > 18 years with metastatic [organ] carcinoma with a biomarker Y+ mutation who have shown progression after first line treatment and are treated with standard care |
| **Type(s) of data:** | Patient registry data including linked electronic health records, insurance claims, death, and patient reported outcomes | Patient registry data including linked electronic health records, insurance claims, death, and patient reported outcomes |
| **Data linkage:** | Linked based on universal patient identifier in [anonymous country] | Linked based on universal patient identifier in [anonymous country] |
| **Conversion to CDM*:** | n/a | n/a |
| **Software for data management:** | SAS 9.4, My SQL 15.0.2000.5 | SAS 9.4, My SQL 15.0.2000.5 |

*CDM = Common Data Model

- 1. Data management

The research team operates a secure, state-of-the-art, computing facility. The computer cluster is Linux-based and offers SAS 9.4, Stata 15.1, and R. The data center is a secure facility that houses both our computing environment as well as clinical systems and electronic medical records for several large hospitals in WA. Entry into the computer room requires passing through staffed building security, a successful palm scan, and then passing through staffed computer room security. The research machines are connected to the networking backbone with 10 gigabit-per-second fiber links. Network security is overseen by Information Security, who apply the same standards used for the hospitals electronic medical records systems to the research teams data. All data are transmitted to programmers' workstations in an encrypted state. Backups are created using 256-bit AES encryption, the current Department of Defense standard for data security, and are stored in a locked facility.

The Data Manager will securely download data from the various sources to the servers in the computing cluster via secure SFTP. Data location, contents and data use agreements will be logged. Access to the servers are strictly controlled via physical and technical means to ensure that only individuals with proper clearance and authorization are able to access research data. When a project is closed, the research data are destroyed using a “shred” secure file deletion tool to ensure that sensitive data can never be retrieved.

ata cleaning and descriptive analyses were performed in IBM

SPSS (version 23). Regression models were developed in Stata Corp.

STATA (version 14.1).

ata cleaning and descriptive analyses were performed in IBM

SPSS (version 23). Regression models were developed in Stata Corp.

STATA (version 14.1).

Data cleaning, descriptive and regression analyses will be performed with SAS (version 9.4).

- 1. Quality control

The data sources have been through extensive quality control procedures and documentation of the data and collection procedures is provided at www.redactedlink.com. The research group has an internal quality check process which includes assessment of reliability and conformance to expected plausible values. Issues are flagged for review by the data quality team and resolved with documentation of decisions made to clean the data (see appendix).

- 1. Study size and feasibility

We anticipate a sample size of approximately 5,000 patients exposed to Drug A/B in the XYZ Data Registry and an event rate of 35% for the primary outcome of death over a 1 year follow up based on feasibility counts using historical data. The power calculation for a 1:1 matched propensity score cohort analysis indicates more than 80% power to detect a HR of 0.90.

Power calculations are based on the formulas from Chow et al. (2008).

#### Table 13. Power and sample size

1. Limitation of the methods

There are several potential limitations with the methods specified in this protocol.

1. Some important variables may not be collected or will be measured imperfectly
   1. We have selected validated algorithms when possible
   2. We have created proxies for important variables that are not directly captured in the data to reduce confounding by unmeasured factors
2. There will not be randomization
   1. We have emulated the design of a target trial
   2. We have balanced compared groups on important risk factors for the outcome(s)
3. On treatment follow up may be short in real-world practice, there is potential for informative censoring
   1. The results may not capture efficacy of long-term treatment but can measure effectiveness in populations as they are actually treated.
   2. We will do sensitivity analyses regarding reasons for censoring and incorporating censoring weights
4. Protection of human subjects

The study proposal has been reviewed and approved by the [redacted organization] ethics review board to ensure ethical treatment of human subjects as well as privacy protections. The proposed study is observational research that makes secondary use of data collected as part of routine care as well as patient reported outcomes. The project does not involve any intervention, alteration in standard clinical care or use of any procedure in patients. Therefore, there will be no adverse events related to the study itself. All personal identifiers will be encrypted. This encryption minimizes the risk of patient reidentification in the unlikely event of a breach in data security. The institution’s uses standard-issue virus protection software and access to data is controlled through the use of individual passwords known only to study staff. Study staff are required to complete the ABC training prior to being allowed to work on any data and are regularly re-certificated. As a further layer of privacy protection, cell sizes less than 11 will be suppressed in results tables.

1. Reporting of adverse events

The proposed study is observational research that makes secondary use of data collected as part of routine care and does not involve any intervention or alteration in clinical care. Therefore, reporting of adverse events related to this study is not applicable. Safety evaluations for this study are limited to the specified safety outcomes stated in section 4.4.2.

1. References

[Redacted list of references.]

1. Appendices

See excel files.

Appendix A - study population entry criteria (exposure)

Appendix B - drug, diagnosis and procedure based inclusion/exclusion criteria

Appendix C - drug, diagnosis and procedure based covariates

Appendix D - outcomes

Appendix E - care setting
