# Supplementary material for: HARmonized Protocol Template to Enhance Reproducibility of hypothesis evaluating real‐world evidence studies on treatment effects: A good practices report of a joint ISPE/ISPOR task force
Source: Pharmacoepidemiol Drug Saf. 2022 Oct 10;32(1):44–55. doi: 10.1002/pds.5507 (PMC9771861; doi:10.1002/pds.5507)
Supplement: Supplementary file 3 — Appendix 3. Example use cases. [file PDS-32-44-s004.zip › Appendix 3/Example 3 Case-control.docx]

# 1. Title Page

DISCLAIMER: This protocol is based on the research question and design from a published study^1^, but there may be differences in exact scientific decisions. Some of the information needed to complete the protocol template was not available from the publication. For the purposes of populating the protocol template, the subgroup working on this example use case made fictional or reasonable choices that may not be reflective of the scientific decisions made by the original investigators. Abbreviated entries are provided to illustrate how to use the protocol template, however appendices were not prepared for this example protocol.

| Title | Reproduction of a study on the use of pioglitazone and the risk of bladder cancer in people with type 2 diabetes |
| --- | --- |
| Research question & Objectives | To determine if the use of pioglitazone is associated with an increased risk of incident bladder cancer in people with type 2 diabetes. |
| Protocol version | V1 |
| Last update date | 1/1/2022 |
| Contributors | **Primary investigator contact information:**  John Doe  **Contributor names:**  Jane Smith |
| Study registration | **Site:** Real World Evidence Registry: https://osf.io/registries/rwe/discover  **Identifier:** doi:abc123 |
| Sponsor | **Organization:** ABC Institutional Review Board  **Contact:** IRB123 |
| Conflict of interest | None |

Table of contents

[1. Title Page 1](#_Toc102631443)

[2. Abstract 4](#_Toc102631444)

[3. Amendments and updates 4](#_Toc102631445)

[4. Milestones 5](#_Toc102631446)

[Table 1 Milestones 5](#_Toc102631447)

[5. Rationale and background 5](#_Toc102631448)

[6. Research question and objectives 5](#_Toc102631449)

[Table 2 Primary and secondary research questions and objective 5](#_Toc102631450)

[7. Research methods 6](#_Toc102631451)

[7.1. Study design 6](#_Toc102631452)

[7.2. Study design diagram 7](#_Toc102631453)

[7.3. Setting 7](#_Toc102631454)

[7.3.1 Context and rationale for definition of time 0 (and other primary anchors) for entry to the study population 7](#_Toc102631455)

[Table 3 Operational Definition of Time 0 (index date) and other primary anchors 8](#_Toc102631456)

[7.3.2 Context and rationale for study inclusion criteria: 8](#_Toc102631457)

[Table 4. Operational Definitions of Inclusion Criteria 8](#_Toc102631458)

[7.3.3 Context and rationale for study exclusion criteria 9](#_Toc102631459)

[Table 5. Operational Definitions of Exclusion Criteria 9](#_Toc102631460)

[7.4. Variables 9](#_Toc102631461)

[7.4.1 Context and rationale for exposure(s) of interest 9](#_Toc102631462)

[Table 6. Operational Definitions of Exposure 10](#_Toc102631463)

[7.4.2 Context and rationale for outcome(s) of interest 10](#_Toc102631464)

[Table 7. Operational Definitions of Outcome 10](#_Toc102631465)

[7.4.3 Context and rationale for follow up 10](#_Toc102631466)

[Table 8. Operational Definitions of Follow Up 11](#_Toc102631467)

[7.4.4 Context and rationale for covariates (confounding variables and effect modifiers, e.g. risk factors, comorbidities, comedications) 11](#_Toc102631468)

[Table 9. Operational Definitions of Covariates 11](#_Toc102631469)

[7.5. Data analysis 12](#_Toc102631470)

[7.5.1 Context and rationale for analysis plan 12](#_Toc102631471)

[Table 10. Primary, secondary, and subgroup analysis specification 12](#_Toc102631472)

[Table 11. Sensitivity analyses – rationale, strengths and limitations 13](#_Toc102631473)

[7.6. Data sources 14](#_Toc102631474)

[7.6.1 Context and rationale for data sources 14](#_Toc102631475)

[Table 12. Metadata about data sources and software 14](#_Toc102631476)

[7.7. Data management 15](#_Toc102631477)

[7.8. Quality Control 15](#_Toc102631478)

[7.9. Study size and feasibility 15](#_Toc102631479)

[8. Limitation of the methods 15](#_Toc102631480)

[9. Protection of human subjects 16](#_Toc102631481)

[10. Reporting of adverse events 16](#_Toc102631482)

[11. References 16](#_Toc102631483)

[12. Appendices 16](#_Toc102631484)

2. Abstract

Pioglitazone is a thiazolidinedione antidiabetic agent that is effective at reducing glycated haemoglobin (HbA1c) levels and may decrease the risk of cardiovascular events. It has been associated with weight gain and an increased risk of congestive heart failure. There is conflicting evidence that pioglitazone may be associated with an increased risk of bladder cancer. The association between use of pioglitazone and bladder cancer is controversial.

This study will evaluate the reproducibility of a prior study’s findings on pioglitazone and increased risk of bladder cancer.

We will use the same database as used in the previously published study that we are trying to reproduce. The original study used the CPRD research database, a UK general practice research database is the largest database on electronic health records from primary care in the world. It is representative of the UK population and has been widely used for research purposes. There is extensive documentation and validation of the database (https://www.cprd.com/primary-care).

3. Amendments and updates

| **Version date** | **Version number** | **Section of protocol** | **Amendment or update** | **Reason** |
| --- | --- | --- | --- | --- |
| 1/1/2022 | 1 | First draft | n/a | n/a |

1. Milestones

#### Table 1 Milestones

| **Milestone** | **Date** |
| --- | --- |
| Feasibility counts | 9/15/2021 |
| Draft 1 of protocol complete | 1/1/2022 |
| Registration of protocol | 1/1/2022 |
| Study progress report 1 | 6/30/2022 |
| Study progress report 2 | 12/31/2022 |
| Final report of study results | 6/30/2023 |

1. Rationale and background

**What is known about the condition:** There is conflicting evidence that pioglitazone may be associated with an increased risk of bladder cancer.^1-3^

**What is known about the exposure of interest:** Pioglitazone is effective at reducing glycated haemoglobin (HbA1c) levels and may decrease the risk of cardiovascular events. It has also been associated with weight gain and an increased risk of congestive heart failure.^2^

**Gaps in knowledge:** The association between use of the thiazolidinedione antidiabetic agent pioglitazone and bladder cancer is controversial.

**What is the expected contribution of this study?** To evaluate the reproducibility of findings on pioglitazone and increased risk of bladder cancer.

1. Research question and objectives

#### Table 2 Primary and secondary research questions and objective

1. **Primary research question and objective**

| **Objective:** | To determine if the use of pioglitazone is associated with an increased risk of incident bladder cancer in people with type 2 diabetes. |
| --- | --- |
| **Hypothesis:** | Pioglitazone increase the risk of incident bladder cancer. |
| **Population *(mention key inclusion-exclusion criteria):*** | First ever oral antidiabetic agent users. The antidiabetic agents considered at cohort entry consisted of sulfonylureas (for example, glyburide, gliclazide, glipizide), metformin, thiazolidinediones (pioglitazone, rosiglitazone), and other oral hypoglycaemic agents (meglitinides, dipeptidyl peptidase-4 inhibitors, alpha-glucosidase inhibitors, glucagon-like peptide-1 analogues, and guar gum). Patients who started treatment with insulin were excluded as they were more likely to have type 1 diabetes or advanced type 2 diabetes. Patients who started insulin during follow-up, however, were retained in the cohort. |
| **Exposure:** | Pioglitazone |
| **Comparator:** | Non-use of thiazolidinediones |
| **Outcome:** | Bladder cancer |
| **Time *(when follow up begins and ends):*** | Follow up for the cohort began the day after initiation of therapy with any antidiabetic until the first of bladder cancer, death from any cause, end of registration with the general practice, or end of the study period. All incident cases of bladder cancers occurring during follow-up were identified. Exposure to pioglitazone was evaluated at any time from entry to the cohort until 1 year before incident bladder cancer. |
| **Setting:** | Outpatient care |
| **Main measure of effect:** | Odds ratio |

1. **Secondary research question 1 and objective**

n/a

1. Research methods
   1. Study design

**Research design (e.g. cohort, case-control, etc.):** Nested case-control study

**Rationale for study design choice:** time varying nature of drug use, the size of the cohort, and the long duration of follow-up

- 1. Study design diagram


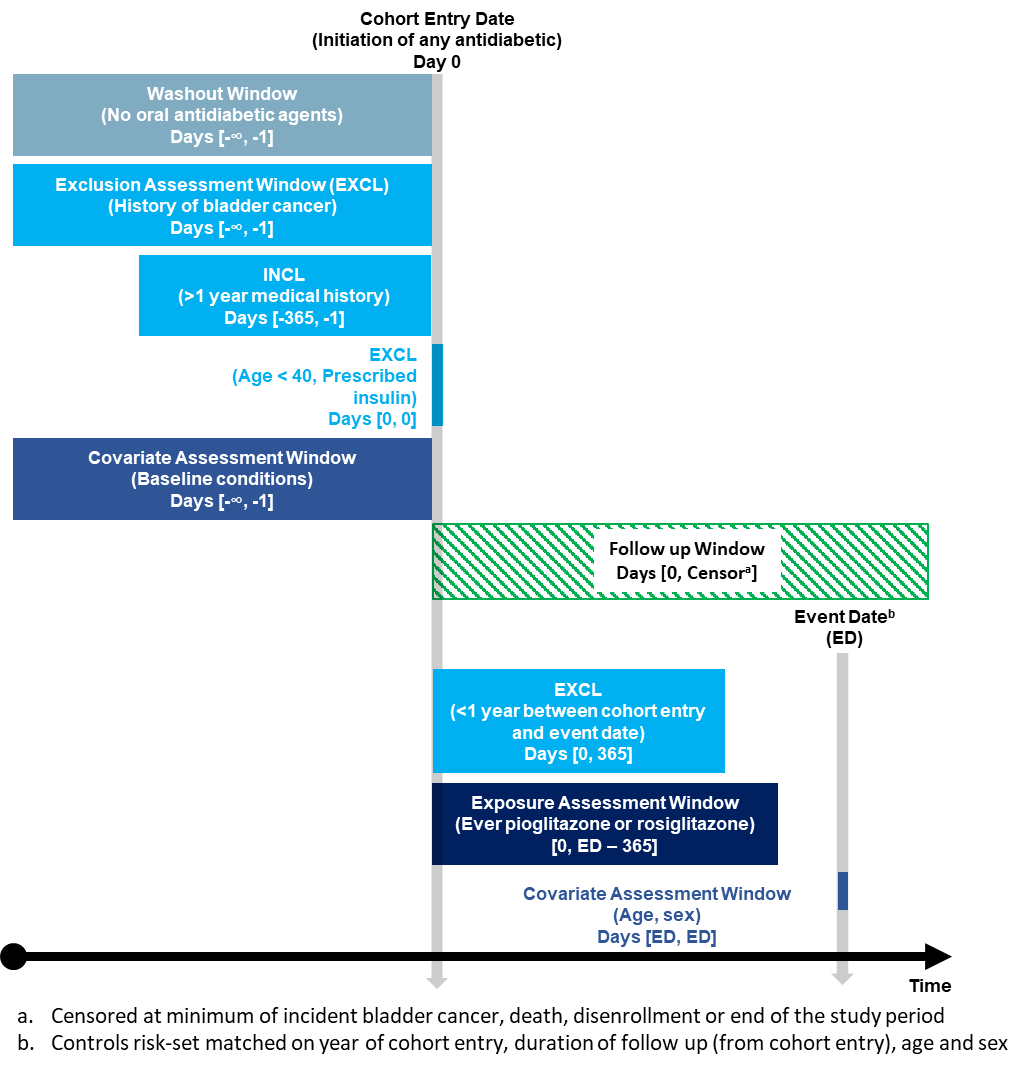


- 1. Setting

#### 7.3.1 Context and rationale for definition of time 0 (and other primary anchors) for entry to the study population

Patients entered the base cohort based on initiation of any antidiabetic agent. This allowed identification of newly treated patients with diabetes.

#### Table 3 Operational Definition of Time 0 (index date) and other primary anchors

| **Study population name(s)** | **Time Anchor Description**  **(e.g. time 0)** | **Number of entries** | **Type of entry** | **Washout window** | **Care Setting^1^** | **Code Type^2^** | **Diagnosis position** | **Incident with respect to…** | **Measurement characteristics/validation** | **Source of algorithm** |
| --- | --- | --- | --- | --- | --- | --- | --- | --- | --- | --- |
| Treated diabetes | Date of incident dispensation for any antidiabetic  (Time 0) | Single | Incident | [-∞, -1] | n/a | DM+D | n/a | All diabetic agents | No validation study | Investigator review of generic names |
| Incident Bladder Cancer | 1^st^ bladder cancer code  (Event date – ED) | Single | Incident | [-∞, ED] | n/a | Read code | any | All bladder cancer codes | No validation study | Investigator review of Read codes |
| Matched controls | Date of bladder cancer for case that the control is matched to (Event date – ED) | Single | Incident | [-∞, ED] | n/a | n/a | any | All bladder cancer codes | No validation study | Investigator review of Read codes |

^1^ GP = general practice, n/a = not applicable

^2^ See appendix for listing of clinical codes for each study parameter

#### 7.3.2 Context and rationale for study inclusion criteria:

We require at least one year of up-to-standard research data prior to entry to the cohort to ensure that we are able to capture baseline history of disease and medication use.

#### Table 4. Operational Definitions of Inclusion Criteria

| **Criterion** | **Details** | **Order of application** | **Assessment window** | **Care Settings¹** | **Code Type^2^** | **Diagnosis position^3^** | **Applied to study populations:** | **Measurement characteristics/validation** | **Source for algorithm** |
| --- | --- | --- | --- | --- | --- | --- | --- | --- | --- |
| Observable time | Defined as time between the latest of the up-to-standard date or the current registration date and the earliest of the last collection date, transferred out date, or date of death. | Before selection of index date | [-365, 1] | Any | n/a | n/a | Treated diabetes | No validation study | Investigator defined |

^1^ GP = general practice, n/a = not applicable

^2^ See appendix for listing of clinical codes for each study parameter

^3^ Specify whether a diagnosis code is required to be in the primary position (main reason for encounter)

#### 7.3.3 Context and rationale for study exclusion criteria

We exclude younger adults who are at lower risk of bladder cancer, patients who receive insulin as their first antidiabetic agent because these patients may have type 1 diabetes, and patients who have a history of bladder cancer because we are evaluating the risk of incident bladder cancer.

#### Table 5. Operational Definitions of Exclusion Criteria

| **Criterion** | **Details** | **Order of application** | **Assessment window** | **Care Settings¹** | **Code Type^2^** | **Diagnosis position^3^** | **Applied to study populations:** | **Measurement characteristics/validation** | **Source for algorithm** |
| --- | --- | --- | --- | --- | --- | --- | --- | --- | --- |
| Age <40 yrs |  | Before selection of index date | [0, 0] | n/a | n/a | n/a | Treated diabetes | No validation study | n/a |
| Insulin |  | Before selection of the index date | [0, 0] | n/a | DM+D | n/a | Treated diabetes | No validation study | Investigator review of generic names |
| History of bladder cancer |  | Before selection of index date | [-∞,-1] | Any | Read codes | Any | Treated diabetes | No validation study | Investigator review of Read codes |

^1^ GP = general practice, n/a = not applicable

^2^ See appendix for listing of clinical codes for each study parameter

^3^ Specify whether a diagnosis code is required to be in the primary position (main reason for encounter)

- 1. Variables

#### 7.4.1 Context and rationale for exposure(s) of interest

The exposure of interest is pioglitazone use, defined as a prescription occurring between cohort entry and up to one year before the date of incident bladder cancer. A one-year lag was built in to accommodate latency of exposure effect.

**Algorithm to define duration of exposure effect:**

Exposure will be classified as ever/never as described above, on the basis of at least one prescription for pioglitazone in the relevant exposure assessment window.

#### Table 6. Operational Definitions of Exposure

| **Exposure group name(s)** | **Detail** | **Washout window** | **Assessment Window** | **Care Setting^1^** | **Code Type^2^** | **Diagnosis position^3^** | **Applied to study populations:** | **Incident with respect to…** | **Measurement characteristics/validation** | **Source of algorithm** |
| --- | --- | --- | --- | --- | --- | --- | --- | --- | --- | --- |
| Pioglitazone |  | [-∞, -1] | [0, ED-365] | n/a | DM+D | n/a | Treated diabetes | Thiazolidines | No validation study | Investigator review of generic names |
| Non-use of thiazolidines |  | [-∞, -1] | [0, ED-365] | n/a | DM+D | n/a | Treated diabetes | Thiazolidines | No validation study | Investigator review of generic names |

^1^ GP = general practice, n/a = not applicable

^2^ See appendix for listing of clinical codes for each study parameter

^3^ Specify whether a diagnosis code is required to be in the primary position (main reason for encounter)

#### 7.4.2 Context and rationale for outcome(s) of interest

The outcome of interest is incident bladder cancer. The relationship to pioglitazone is of interest because of controversial findings from prior studies.

#### Table 7. Operational Definitions of Outcome

| **Outcome name** | **Details** | **Primary outcome?** | **Type of outcome** | **Washout window** | **Care Settings¹** | **Code Type^2^** | **Diagnosis Position^3^** | **Applied to study populations:** | **Outcome measurement characteristics/**  **validation** | **Source of algorithm** |
| --- | --- | --- | --- | --- | --- | --- | --- | --- | --- | --- |
| Incident Bladder Cancer |  | Yes | Incident | [-∞, ED] | Any | Read code | Any | Treated diabetes | No validation study | Investigator review of Read codes |

^1^ GP = general practice, n/a = not applicable

^2^ See appendix for listing of clinical codes for each study parameter

^3^ Specify whether a diagnosis code is required to be in the primary position (main reason for encounter)

#### 7.4.3 Context and rationale for follow up

The follow up for the cohort in which the case-control study is nested is described in the table below. Up to 20 controls will be risk-set sampled from the cohort for each identified incident bladder cancer case. Controls will be matched on year of birth, year of cohort entry, sex, and duration of follow-up. The event date (ED) for controls will be the ED for the cases to whom they are matched. The controls are sampled from the same cohort that gave rise to the cases, therefore the same inclusion-exclusion criteria and censoring criteria are applied. This means that all controls will be alive, have had no prior diagnoses for bladder cancer, and have up-to-standard follow up over the same time-period.

#### Table 8. Operational Definitions of Follow Up

|  |  |  |  |
| --- | --- | --- | --- |
| **Follow up start** | Day 1 |  |  |
| **Follow up end^1^** | **Select all that apply** |  | **Specify** |
| **Date of outcome** | Yes |  |  |
| **Date of death** | Yes |  |  |
| **End of observation in data** | Yes |  | End of up-to-standard data, defined as the earliest of the last collection date, transferred out date, or date of death |
| **Day X following index date**  *(specify day)* | No |  |  |
| **End of study period**  (specify date) | Yes |  | 31 Dec 2009 |
| **End of exposure**  *(specify operational details,*  *e.g. stockpiling algorithm, grace period)* | No |  |  |
| **Date of add to/switch from exposure**  *(specify algorithm)* | No |  |  |
| **Other date** *(specify)* | No |  |  |

^1^ Follow up ends at the first occurrence of any of the selected criteria that end follow up.

#### 7.4.4 Context and rationale for covariates (confounding variables and effect modifiers, e.g. risk factors, comorbidities, comedications)

We will measure demographic, comorbidity, healthcare utilization, frailty and socioeconomic status related risk factors for bladder cancer that are associated with exposure to pioglitazone.

#### Table 9. Operational Definitions of Covariates

| **Characteristic** | **Details** | **Type of variable** | **Assessment window** | **Care Settings¹** | **Code Type^2^** | **Diagnosis Position^3^** | **Applied to study populations:** | **Measurement characteristics/validation** | **Source for algorithm** |
| --- | --- | --- | --- | --- | --- | --- | --- | --- | --- |
| hbA1c | Most recent measure before day 0 | Continuous | [-∞, -1] | n/a | n/a | n/a | Treated diabetes | Unknown | Lab results |
| Excessive alcohol use | Alcohol related disorders, alcoholic cirrhosis of liver, alcoholic hepatitis, etc | Binary | [-∞, -1] | Any | Read | Any | Treated diabetes | No validation study | Investigator review of Read codes |
| Obesity | body mass  index ≥30 kg/m^2^)  Most recent measure before day 0 | Binary | [-∞, -1] | n/a | n/a | n/a | Treated diabetes | No validation study | Clinical measurement |
| Prior cancer | Other than non-melanoma skin cancer | Binary | [-∞, -1] | Any | Read | Any | Treated diabetes | No validation study | Investigator review of Read codes |
| Prior bladder conditions | Cystitis, bladder stones, etc | Binary | [-∞, -1] | Any | Read | Any | Treated diabetes | No validation study | Investigator review of Read codes |
| Modified Charlson comorbidity score | Modified for CPRD and removed diabetes and cancer components | Binary | [-∞, -1] | Any | Read | Any | Treated diabetes | No validation study | Investigator review of Read codes |
| Other antidiabetic agents | Defined as at least 1 prescription for metformin, sulfonylureas, insulins, and other oral hypoglycaemic agents), | Binary | [-∞, -1] | Any | DM+D | Any | Treated diabetes | No validation study | Investigator review of generic names |

^1^ IP = inpatient, OP = outpatient, ED = emergency department, OT = other, n/a = not applicable

^2^ See appendix for listing of clinical codes for each study parameter

^3^ Specify whether a diagnosis code is required to be in the primary position (main reason for encounter)

- 1. Data analysis

#### 7.5.1 Context and rationale for analysis plan

We will use conditional logistic regression to analyse this nested case-control study. This will allow us to estimate rate ratios and confidence intervals for the relationship between pioglitazone use and incident bladder cancer.

#### Table 10. Primary, secondary, and subgroup analysis specification

1. **Primary analysis**

| **Hypothesis:** | Prior pioglitazone use increases the risk of incident bladder cancer |
| --- | --- |
| **Exposure contrast:** | Ever pioglitazone exposed versus never exposed to thiazolidinediones |
| **Outcome:** | Incident bladder cancer |
| **Analytic software:** | SAS v9.2 |
| **Model(s):**  ***(provide details or code)*** | Conditional logistic regression  proc logistic data=analysis_data;  strata year_of_birth year_of_cohortentry sex duration_followup;  model outcome(event='1')=exposure hbA1c Excessive_alcohol_use Obesity Prior_cancer Prior_bladder_conditions Modified_Charlson  Other_antidiabetic;  run; |
| **Confounding adjustment method** | ***Name method and provide relevant details, e.g. bivariate, multivariable, propensity score matching (specify matching algorithm ratio and caliper), propensity score weighting (specify weight formula, trimming, truncation), propensity score stratification (specify strata definition), other.*** |
|  | We will use an logistic regression model for the outcome that is conditioned on (stratified) by year of birth, year of cohort entry, and duration of follow up (categorical variable, monthly intervals) with multivariable adjustment for baseline hbA1c, excessive alcohol use, obesity, prior cancer, prior bladder conditions, modified Charlson, and other antidiabetic use. |
| **Missing data methods** | ***Name method and provide relevant details, e.g. missing indicators, complete case, last value carried forward, multiple imputation (specify model/variables), other.*** |
|  | We will use multiple imputation for missing values of baseline hbA1c. |
| **Subgroup Analyses** | ***List all subgroups*** |
|  | Not applicable. |

#### Table 11. Sensitivity analyses – rationale, strengths and limitations

|  | **What is being varied? How?** | **Why?  (What do you expect to learn?)** | **Strengths of the sensitivity analysis compared to the primary** | **Limitations of the sensitivity analysis compared to the primary** |
| --- | --- | --- | --- | --- |
| Sensitivity analysis 1 | We will compare ever users of pioglitazone to ever users of rosiglitazone | The impact of potential confounding by indication | Active comparator versus non-user comparator | Assumes that the confounding structure is similar for pioglitazone and rosiglitazone, re: bladder cancer |
| Sensitivity analysis 2 | Evaluate a dose response relationship instead of ever/never | We will learn whether there is a dose dependent relationship between pioglitazone and incident bladder cancer | This provides more insight on the biological mechanism and plausibility of the relationship between pioglitazone and incident bladder cancer | There may be more potential for exposure misclassification using a cumulative dose/duration measure for exposure when using a prescription based database |

- 1. Data sources

#### 7.6.1 Context and rationale for data sources

**Reason for selection:** The UK general practice research database is the largest database on electronic health records from primary care in the world.^4^ It is representative of the UK population, and has been widely used for research purposes.

**Strengths of data source(s):** The database contains longitudinal records from primary care, where the general practitioners are specifically trained to collect information using a standardised form. All prescriptions from general practitioners are recorded based on the UK Prescription Pricing Authority Dictionary, medical diagnoses and procedures are captured via Read codes, and lifestyle/clinical variables such as body mass index and smoking are also captured.

**Limitations of data source(s):** The data source reflects primary care electronic health records and may miss elements of care received by specialty care providers or hospitalizations.

**Data source provenance/curation:** There is extensive documentation and validation of the database (https://www.cprd.com/primary-care).

#### Table 12. Metadata about data sources and software

|  | **Data 1** |
| --- | --- |
| **Data Source(s):** | UK General Practice Research Database |
| **Study Period:** | Jan 1 1988 – Dec 31 2009 |
| **Eligible Cohort Entry Period:** | Jan 1 1989 – Dec 31 2009 |
| **Data Version (or date of last update):** | V7.2.1 |
| **Data sampling/extraction criteria:** | n/a |
| **Type(s) of data:** | Primary care practice electronic health record |
| **Data linkage:** | n/a |
| **Conversion to CDM*:** | n/a |
| **Software for data management:** | SAS v9.2 |

*CDM = Common Data Model

- 1. Data management

The research team operates a secure, state-of-the-art, computing facility. The computer cluster is Linux-based and offers SAS 9.2, Stata 15.1, and R. Entry into the computer room requires passing through staffed building security, a successful palm scan, and then passing through staffed computer room security. The research machines are connected to the networking backbone with 10 gigabit-per-second fiber links. Network security is overseen by Information Security, who apply the same standards used for the hospitals electronic medical records systems to the research teams data. All data are transmitted to programmers' workstations in an encrypted state. Backups are created using 256-bit AES encryption, the current Department of Defense standard for data security, and are stored in a locked facility.

The Data Manager will securely download data to the servers in the computing cluster via secure SFTP. Data location, contents and data use agreements will be logged. Access to the servers are strictly controlled via physical and technical means to ensure that only individuals with proper clearance and authorization are able to access research data. When a project is closed, the research data are destroyed using a “shred” secure file deletion tool to ensure that sensitive data can never be retrieved.

ata cleaning and descriptive analyses were performed in IBM

SPSS (version 23). Regression models were developed in Stata Corp.

STATA (version 14.1).

ata cleaning and descriptive analyses were performed in IBM

SPSS (version 23). Regression models were developed in Stata Corp.

STATA (version 14.1).

Data cleaning, descriptive and regression analyses will be performed with SAS 9.2.

- 1. Quality Control

The data source has been through extensive quality control procedures and documentation of the data and collection procedures is provided at https://www.cprd.com/primary-care. The research group has an internal quality check process which includes assessment of reliability and conformance to expected plausible values. Issues are flagged for review by the data quality team and resolved with documentation of decisions made to clean the data (see appendix).

- 1. Study size and feasibility

This protocol describes an attempt to reproduce the findings of another study using the same data source.^1^ The original study had a sample size of 241,111 treated diabetic patients. There were 470 in this cohort that developed incident bladder cancer. This provided sufficient power to detect a nearly 2-fold elevation in risk for pioglitazone compared to non-exposure with 95% confidence intervals excluding the null.

1. Limitation of the methods

Discuss potential limitations of the study design, data sources, and analytic methods, including issues relating to confounding, bias, generalisability, and random error. Discuss the steps will be taken to reduce the potential impact of these limitations.

1. The EHR database includes prescriptions, not dispensing data. Some patients may not have filled their prescriptions or adhered to the prescribed therapy. Prescriptions written by non-primary care doctors were not recorded. However, the expected direction of non-differential misclassification of exposure would be toward the null.
2. Pioglitazone is prescribed to patients at more advanced stages of diabetes, and more advanced disease may be associated with higher risk of bladder cancer. The covariates specified in the protocol were selected to try and proxy adjust for this.
3. Cancer staging information is not well captured in primary care and the performance characteristics of the outcome algorithm are unknown.
4. Protection of human subjects

The study proposal has been reviewed and approved by the ABC ethics review board to ensure ethical treatment of human subjects as well as privacy protections. The proposed study is observational research that makes secondary use of data collected as part of routine care as well as patient reported outcomes. The project does not involve any intervention, alteration in standard clinical care or use of any procedure in patients. Therefore, there will be no adverse events related to the study itself. All personal identifiers will be encrypted. This encryption minimizes the risk of patient reidentification in the unlikely event of a breach in data security. The institution’s uses standard-issue virus protection software and access to data is controlled through the use of individual passwords known only to study staff. Study staff are required to complete the ABC training prior to being allowed to work on any data and are regularly re-certificated. As a further layer of privacy protection, cell sizes less than 11 will be suppressed in results tables.

1. Reporting of adverse events

The proposed study is observational research that makes secondary use of data collected as part of routine care and does not involve any intervention or alteration in clinical care. Therefore, reporting of adverse events related to this study is not applicable. Safety evaluations for this study are limited to the specified safety outcomes stated in section 4.4.2.

1. References

1. Azoulay L, Yin H, Filion KB, et al. The use of pioglitazone and the risk of bladder cancer in people with type 2 diabetes: nested case-control study. *BMJ : British Medical Journal*. 2012;344doi:10.1136/bmj.e3645

2. Dormandy JA, Charbonnel B, Eckland DJ, et al. Secondary prevention of macrovascular events in patients with type 2 diabetes in the PROactive Study (PROspective pioglitAzone Clinical Trial In macroVascular Events): a randomised controlled trial. *Lancet*. Oct 8 2005;366(9493):1279-89. doi:10.1016/S0140-6736(05)67528-9

3. Piccinni C, Motola D, Marchesini G, Poluzzi E. Assessing the association of pioglitazone use and bladder cancer through drug adverse event reporting. *Diabetes care*. Jun 2011;34(6):1369-71. doi:10.2337/dc10-2412

4. Walley T, Mantgani A. The UK General Practice Research Database. *Lancet*. Oct 11 1997;350(9084):1097-9. doi:10.1016/S0140-6736(97)04248-7

1. Appendices

See excel files.

Appendix A - study population entry criteria (exposure)

Appendix B - drug, diagnosis and procedure based inclusion/exclusion criteria

Appendix C - drug, diagnosis and procedure based covariates

Appendix D - outcomes

Appendix E - care setting

Appendix F – Data dictionaries and documentation
