# Supplementary material for: HARmonized Protocol Template to Enhance Reproducibility of hypothesis evaluating real‐world evidence studies on treatment effects: A good practices report of a joint ISPE/ISPOR task force
Source: Pharmacoepidemiol Drug Saf. 2022 Oct 10;32(1):44–55. doi: 10.1002/pds.5507 (PMC9771861; doi:10.1002/pds.5507)
Supplement: Supplementary file 3 — Appendix 3. Example use cases. [file PDS-32-44-s004.zip › Appendix 3/Example 4 Pregnancy safety.docx]

# 1. Title Page

DISCLAIMER: This protocol is based on the research question and design from a published study^1^, but there may be differences in exact scientific decisions. Some of the information needed to complete the protocol template was not available from the publication. For the purposes of populating the protocol template, the subgroup working on this example use case made fictional or reasonable choices that may not be reflective of the actual scientific decisions made by the original investigators. Abbreviated entries are provided to illustrate how to use the protocol template, however appendices were not prepared for this example protocol.

| Title | Replication of study evaluating topiramate use early in pregnancy and the risk of oral clefts |
| --- | --- |
| Research question & Objectives | To assess the relative risk of oral clefts associated with maternal use of high and low doses of topiramate during the first trimester for epilepsy and nonepilepsy indications |
| Protocol version | V1 |
| Last update date | 12/14/2021 |
| Contributors | **Primary investigator contact information:**  Jane Smith  **Contributor names:**  John Doe |
| Study registration | **Site:** Real World Evidence Registry: https://osf.io/registries/rwe/discover  **Identifier:** doi:abc123 |
| Sponsor | **Organization:** ABC Institutional Review Board  **Contact:** IRB123 |
| Conflict of interest | None |

Table of contents

[1. Title Page 1](#_Toc102631485)

[2. Abstract 4](#_Toc102631486)

[3. Amendments and updates 4](#_Toc102631487)

[4. Milestones 5](#_Toc102631488)

[Table 1 Milestones 5](#_Toc102631489)

[5. Rationale and background 5](#_Toc102631490)

[6. Research question and objectives 6](#_Toc102631491)

[Table 2 Primary and secondary research questions and objective 6](#_Toc102631492)

[7. Research methods 7](#_Toc102631493)

[7.1. Study design 7](#_Toc102631494)

[7.2. Study design diagram 8](#_Toc102631495)

[7.3. Setting 9](#_Toc102631496)

[7.3.1 Context and rationale for definition of time 0 (and other primary time anchors) for entry to the study population 9](#_Toc102631497)

[Table 3 Operational Definition of Time 0 (index date) and other primary time anchors 9](#_Toc102631498)

[7.3.2 Context and rationale for study inclusion criteria: 9](#_Toc102631499)

[Table 4. Operational Definitions of Inclusion Criteria 9](#_Toc102631500)

[7.3.3 Context and rationale for study exclusion criteria 10](#_Toc102631501)

[Table 5. Operational Definitions of Exclusion Criteria 10](#_Toc102631502)

[7.4. Variables 10](#_Toc102631503)

[7.4.1 Context and rationale for exposure(s) of interest 10](#_Toc102631504)

[Table 6. Operational Definitions of Exposure 11](#_Toc102631505)

[7.4.2 Context and rationale for outcome(s) of interest 11](#_Toc102631506)

[Table 7. Operational Definitions of Outcome 11](#_Toc102631507)

[7.4.3 Context and rationale for follow up 12](#_Toc102631508)

[Table 8. Operational Definitions of Follow Up 12](#_Toc102631509)

[7.4.4 Context and rationale for covariates (confounding variables and effect modifiers, e.g. risk factors, comorbidities, comedications). 12](#_Toc102631510)

[Table 9. Operational Definitions of Covariates 13](#_Toc102631511)

[7.5. Data analysis 13](#_Toc102631512)

[7.5.1 Context and rationale for analysis plan 13](#_Toc102631513)

[Table 10. Primary, secondary, and subgroup analysis specification 14](#_Toc102631514)

[Table 11. Sensitivity analyses – rationale, strengths and limitations 15](#_Toc102631515)

[7.6. Data sources 15](#_Toc102631516)

[7.6.1 Context and rationale for data sources 15](#_Toc102631517)

[Table 12. Metadata about data sources and software 15](#_Toc102631518)

[7.7. Data management 16](#_Toc102631519)

[7.8. Quality control 17](#_Toc102631520)

[7.9. Study size and feasibility 17](#_Toc102631521)

[8. Limitation of the methods 17](#_Toc102631522)

[9. Protection of human subjects 17](#_Toc102631523)

[10. Reporting of adverse events 18](#_Toc102631524)

[11. References 18](#_Toc102631525)

[12. Appendices 18](#_Toc102631526)

2. Abstract

Topiramate is used for different indications. Multiple studies have shown a 2- to 5-fold increased risk of oral clefts. Most participants in prior studies used topiramate to prevent seizures. Questions remain as to whether the lower doses used for non-epilepsy indications also confer risk. This study will evaluate the reproducibility of a large cohort study that investigated the risk of oral clefts with topiramate use during the first trimester of pregnancy.

We will use Medicaid Analytic eXtract (MAX) and Transformed Medicaid Statistical Information System Analytic Files (TAF) include data for enrollees in Medicaid and in the Children's Health Insurance Program (CHIP) in all 50 states and the District of Columbia. The data source contains longitudinal, date stamped information on patient enrolment, demographics, in and outpatient diagnoses, procedures, admission and discharge dates, and medication dispensing that can be used to capture exposure, key inclusion-exclusion criteria, outcome, and covariates. The data are derived from claims for services received by the patient. The selected data sources are widely used for research and the data holders provide thorough documentation of data contents, assumptions and limitations.

3. Amendments and updates

| **Version date** | **Version number** | **Section of protocol** | **Amendment or update** | **Reason** |
| --- | --- | --- | --- | --- |
| 12/14/2021 | 1 | First draft | n/a | n/a |

1. Milestones

#### Table 1 Milestones

| **Milestone** | **Date** |
| --- | --- |
| Feasibility counts | 9/1/2021 |
| Draft 1 of protocol complete | 12/14/2021 |
| Registration of protocol | 12/14/2021 |
| Study progress report 1 | 6/30/2022 |
| Study progress report 2 | 12/31/2022 |
| Final report of study results | 3/1/2023 |

1. Rationale and background

**What is known about the condition:** Maternal use of topiramate has been associated with oral cleft in infants.

**What is known about the exposure of interest:** Topiramate is used for different indications. Multiple studies have shown a 2- to 5-fold increased risk of oral clefts. Most participants in prior studies used Topiramate to prevent seizures.

**Gaps in knowledge:** Questions remain as to whether the lower doses used for non-epilepsy indications also confer risk.

**What is the expected contribution of this study?** This study will evaluate the reproducibility of a large cohort study that investigated the risk of oral clefts with topiramate use during the first trimester of pregnancy.^1^

1. Research question and objectives

#### Table 2 Primary and secondary research questions and objective

1. **Primary research question and objective**

| **Objective:** | To evaluate whether the finding that 1^st^ trimester exposure to topiramate increases the risk of oral clefts in a population of pregnant women exposed to high and low doses for epilepsy and nonepilepsy indications relative to both untreated women can be reproduced. |
| --- | --- |
| **Hypothesis:** | The risk of oral clefts will be elevated with high dose but not low dose topiramate compared to non-use or use of lamotrigine. |
| **Population *(mention key inclusion-exclusion criteria):*** | Pregnancies in women 12-55 years old that resulted in a live birth |
| **Exposure:** | Topiramate during 1^st^ trimester |
| **Comparator:** | No topiramate or other anticonvulsant use |
| **Outcome:** | Oral cleft |
| **Time *(when follow up begins and ends):*** | For outcome ascertainment, oral cleft at birth was ascertained in the infant records within the first 3 months after the date of birth and maternal records during the first month after delivery. |
| **Setting:** | Outpatient and inpatient care |
| **Main measure of effect:** | Relative risk |

1. **Secondary research question 1 and objective**

| **Objective:** | To evaluate whether the finding that 1^st^ trimester exposure to topiramate increases the risk of oral clefts in a population of pregnant women exposed to high and low doses for epilepsy and nonepilepsy indications relative to women treated with lamotrigine can be reproduced. |
| --- | --- |
| **Hypothesis:** | The risk of oral clefts will be elevated with high dose but not low dose topiramate compared to non-use or use of lamotrigine. |
| **Population *(mention key inclusion-exclusion criteria):*** | Pregnancies in women 12-55 years old that resulted in a live birth |
| **Exposure:** | Topiramate in 1^st^ trimester |
| **Comparator:** | Lamotrigine but no topiramate or other anticonvulsant in 1^st^ trimester |
| **Outcome:** | Oral cleft |
| **Time *(when follow up begins and ends):*** | For outcome ascertainment, oral cleft at birth was ascertained in the infant records within the first 3 months after the date of birth and maternal records during the first month after delivery. |
| **Setting:** | Outpatient and inpatient care |
| **Main measure of effect:** | Relative risk |

1. Research methods
   1. Study design

**Research design (e.g. cohort, case-control, etc.):** Cohort study

**Rationale for study design choice:** We are reproducing a previously conducted study, which used a cohort design.

- 1. Study design diagram


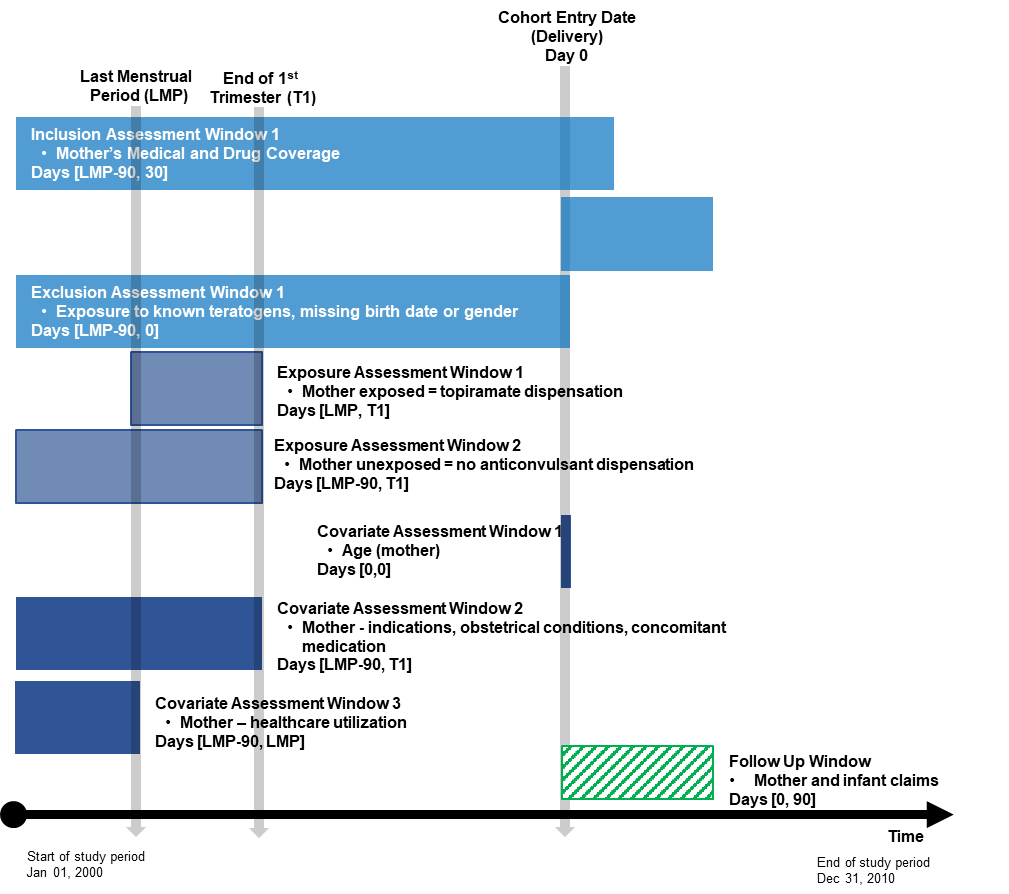


.

- 1. Setting

#### 7.3.1 Context and rationale for definition of time 0 (and other primary time anchors) for entry to the study population

Depending on the study question and design, time 0 can be defined by the date of delivery or the start of pregnancy. In this example, we used the date of delivery as time 0.

#### Table 3 Operational Definition of Time 0 (index date) and other primary time anchors

| **Study population name(s)** | **Time Anchor Description (e.g. time 0)** | **Number of entries** | **Type of entry** | **Washout window** | **Care Setting^1^** | **Code Type^2^** | **Diagnosis position** | **Incident with respect to…** | **Measurement characteristics/validation** | **Source of algorithm** |
| --- | --- | --- | --- | --- | --- | --- | --- | --- | --- | --- |
| Pregnant women | Delivery | Multiple (women with multiple pregnancies are allowed to contribute multiple times) | Incident | See pregnancy algorithm | See pregnancy algorithm | ICD-9 | See pregnancy algorithm | pregnancy | No validation study | Palmsten et al. 2013^2^ |

^1^ IP = inpatient, OP = outpatient, ED = emergency department, OT = other, n/a = not applicable

^2^See appendix for listing of clinical codes for each study parameter

#### 7.3.2 Context and rationale for study inclusion criteria:

Only pregnancies in women of child-bearing age with a live delivery will be included. To ensure that there is sufficient observable time, women must be enrolled in insurance coverage from 3 months before their last menstrual period to 1 month after. To ensure adequate time to capture birth defects, infants were required to have 3 months of insurance coverage post delivery.

#### Table 4. Operational Definitions of Inclusion Criteria

| **Criterion** | **Details** | **Order of application** | **Assessment window** | **Care Settings¹** | **Code Type^2^** | **Diagnosis position^3^** | **Applied to study populations:** | **Measurement characteristics/validation** | **Source for algorithm** |
| --- | --- | --- | --- | --- | --- | --- | --- | --- | --- |
| Pregnancies ended with a live delivery |  | Before selection of index date | See pregnancy algorithm | See pregnancy algorithm | See pregnancy algorithm | See pregnancy algorithm | Pregnant women | No validation study | Palmsten et al. 2013^2^ |
| Observable time | Mother coverage from 3 months before LMP to 1 month after delivery  Infant coverage 3 months after delivery | Before selection of index date | Mother:  [LMP-90, 30]  Infant: [0, 30] | n/a | n/a | n/a | Pregnant women | n/a | n/a |
| Age 12-55 yrs |  | Before selection of index date | [0, 0] | n/a | n/a | n/a | Pregnant women | n/a | n/a |

^1^ IP = inpatient, OP = outpatient, ED = emergency department, OT = other, n/a = not applicable

^2^ See appendix for listing of clinical codes for each study parameter

^3^ Specify whether a diagnosis code is required to be in the primary position (main reason for encounter)

#### 7.3.3 Context and rationale for study exclusion criteria

Pregnancies with documented potential causes of oral clefts other than the exposure of interest (topiramate) will be excluded.

#### Table 5. Operational Definitions of Exclusion Criteria

| **Criterion** | **Details** | **Order of application** | **Assessment window** | **Care Settings¹** | **Code Type^2^** | **Diagnosis position^3^** | **Applied to study populations:** | **Measurement characteristics/**  **validation** | **Source for algorithm** |
| --- | --- | --- | --- | --- | --- | --- | --- | --- | --- |
| Pregnancies exposed to a known teratogenic medication | e.g., warfarin, antineoplastic agents, lithium, isotretinoin, misoprostol, thalidomide | Before selection of index date | [LMP, T1] | n/a | NDC | n/a | Pregnant women | No validation study | Investigator defined |
| Pregnancies with a documented chromosomal abnormality | ICD-9 code 758.xx or 759.81-759.83 | Before selection of index date | [LMP, 0] | n/a | ICD-9 diagnosis | n/a | Pregnant women | No validation study | Investigator defined |

^1^ IP = inpatient, OP = outpatient, ED = emergency department, OT = other, n/a = not applicable

^2^ See appendix for listing of clinical codes for each study parameter

^3^ Specify whether a diagnosis code is required to be in the primary position (main reason for encounter)

- 1. Variables

#### 7.4.1 Context and rationale for exposure(s) of interest

The lack of randomization has to be compensated for by methods that maximize the comparability between the exposed and reference groups. Lamotrigine is a valid active comparator for topiramate given the overlap of some indication (epilepsy and bipolar disorder) and the amount of information supporting its safety for fetal development.

**Algorithm to define duration of exposure effect:**

If a refill occurs before the end of days supply dispensed, add overlapping days to the end of the subsequent dispensing’s day supply. Assume that the effect of a pill lasts for 30 days. Therefore, we allow up to a 30 day gap between a dispensation + days supply and refill. We also add 30 days to the last dispensation + days supply in a treatment episode and consider this “exposed” time.

#### Table 6. Operational Definitions of Exposure

| **Exposure group name(s)** | **Detail** | **Washout window** | **Assessment Window** | **Care Setting^1^** | **Code Type^2^** | **Diagnosis position^3^** | **Applied to study populations** | **Incident with respect to…** | **Measurement characteristics/validation** | **Source of algorithm** |
| --- | --- | --- | --- | --- | --- | --- | --- | --- | --- | --- |
| Exposure: topiramate |  | [LMP-90, LMP] | [LMP, T1] | n/a | NDC | n/a | Pregnant women | Any anticonvulsant other than topiramate | No validation study | Investigator defined |
| Comparator 1: | Non-use of topiramate or other anticonvulsant | [LMP-90, LMP] | [LMP-90, T1] | n/a | NDC | n/a | Pregnant women | Any anticonvulsant | No validation study | Investigator defined |
| Comparator 2 | Lamotrigine use but no other anticonvulsant | [LMP-90, LMP] | [LMP, T1] |  |  |  | Pregnant women | Any convulsant other than lamotrigine | No validation study | Investigator defined |

^1^ IP = inpatient, OP = outpatient, ED = emergency department, OT = other, n/a = not applicable

^2^ See appendix for listing of clinical codes for each study parameter

^3^ Specify whether a diagnosis code is required to be in the primary position (main reason for encounter)

#### 7.4.2 Context and rationale for outcome(s) of interest

Increased risk of oral clefts were observed in prior studies following maternal use of topiramate early in pregnancy.

#### Table 7. Operational Definitions of Outcome

| **Outcome name** | **Details** | **Primary outcome?** | **Type of outcome** | **Washout window** | **Care Settings¹** | **Code Type^2^** | **Diagnosis Position^3^** | **Applied to study populations:** | **Outcome measurement characteristics/**  **validation** | **Source of algorithm** |
| --- | --- | --- | --- | --- | --- | --- | --- | --- | --- | --- |
| Oral cleft |  | Yes | binary | n/a | Inpatient, outpatient | ICD-9-CM diagnosis and procedure codes | Primary | Pregnant women | PPV 91% in Medicaid | Tikkanen J and Heinonen OP. Public Health 1991.^3^ |

^1^ IP = inpatient, OP = outpatient, ED = emergency department, OT = other, n/a = not applicable

^2^ See appendix for listing of clinical codes for each study parameter

^3^ Specify whether a diagnosis code is required to be in the primary position (main reason for encounter)

#### 7.4.3 Context and rationale for follow up

The assessment window for the outcome of oral clefts is the 3 months following delivery. Three months are allowed to detect the outcome, in case it is not immediately diagnosed or captured in the claims record.

#### Table 8. Operational Definitions of Follow Up

|  |  |  |  |
| --- | --- | --- | --- |
| **Follow up start** | Delivery date |  |  |
| **Follow up end^1^** | **Select all that apply** |  | **Specify** |
| **Date of outcome** | No |  | Comment – Oral cleft (congenital malformation) occurred prior to birth. The presence of oral cleft was ascertained in the infant records [0, 90] and/or maternal records [0, 30] |
| **Date of death** | No |  |  |
| **End of observation in data** | No |  |  |
| **Day X following index date**  *(specify day)* | Yes |  | 90 days post delivery from infant records  30 days post delivery from mother records |
| **End of study period**  (specify date) | No |  |  |
| **End of exposure**  *(specify operational details,*  *e.g. stockpiling algorithm, grace period)* | No |  |  |
| **Date of add to/switch from exposure**  *(specify algorithm)* | No |  |  |
| **Other date** *(specify)* | No |  |  |

^1^ Follow up ends at the first occurrence of any of the selected criteria that end follow up.

#### 7.4.4 Context and rationale for covariates (confounding variables and effect modifiers, e.g. risk factors, comorbidities, comedications).

We measured indications for topiramate use, maternal comorbidities, concomitant medications and overall health status as covariates. These were selected due to their potential to act as confounders or effect modifiers.

#### Table 9. Operational Definitions of Covariates

| **Characteristic** | **Details** | **Type of variable** | **Assessment window** | **Care Settings¹** | **Code Type^2^** | **Diagnosis Position^3^** | **Applied to study populations:** | **Measurement characteristics/validation** | **Source for algorithm** |
| --- | --- | --- | --- | --- | --- | --- | --- | --- | --- |
| Age | (cohort entry year - year of birth) | Continuous | [0, 0] | n/a | n/a | n/a | Pregnant women | n/a | n/a |
| Race | White, black, Hispanic, other or unknown | Categorical | Not reported | n/a | n/a | n/a | Pregnant women | No validation study | n/a |
| Obesity | Not defined | Binary | Not reported | n/a | n/a | n/a | Pregnant women | No validation study | n/a |
| Smoking | Not defined | Binary | Not reported | n/a | n/a | n/a | Pregnant women | No validation study | n/a |
| Topiramate indications | i.e., epilepsy or seizures, migraine, bipolar disorder, pain conditions | Categorical | [LMP-90, T1] | Any | ICD-9-CM | Not reported | Pregnant women | No validation study | n/a |
| Comorbidities | e.g., diabetes mellitus, hypertension, depression | Categorical | [LMP-90, T1] | Any | ICD-9-CM | Not reported | Pregnant women | No validation study | n/a |
| Concomitant medication | e.g., antipsychotics, antidepressants, antidiabetic and  antihypertensive medications | Categorical | [LMP, T1] | OP | NDC | OP | Pregnant women | No validation study | n/a |
| Overall health status | e.g., Obstetric Comorbidity Index, number of hospitalizations | Categorical | [LMP-90, LMP] | Any | ICD-9-CM | Not reported | Pregnant women | No validation study | Bateman et al. 2013^4^,  Schneeweiss et al. 2001^5^ |

^1^ IP = inpatient, OP = outpatient, ED = emergency department, OT = other, n/a = not applicable

^2^ See appendix for listing of clinical codes for each study parameter

^3^ Specify whether a diagnosis code is required to be in the primary position (main reason for encounter)

- 1. Data analysis

#### 7.5.1 Context and rationale for analysis plan

We use logistic regression to estimate a propensity score and then use fine-stratification on the propensity score to adjust for confounding. We chose fine stratification to boost power because both the exposure and outcomes are rare.

#### Table 10. Primary, secondary, and subgroup analysis specification

1. **Primary analysis**

| **Hypothesis:** | Maternal use of topiramate early in pregnancy increases the risk of oral cleft in infants |
| --- | --- |
| **Exposure contrast:** | Pregnant women |
| **Outcome:** | Oral cleft |
| **Analytic software:** | SAS 9.3 |
| **Model(s):**  ***(provide details or code)*** | Outcome model: Adjusted RRs were estimated with generalized linear models in which the unexposed pregnancies were weighted by the PS distribution of the topiramate-exposed pregnancies (SAS PROC GENMOD with weight statement and loglink function)  Propensity score model: logistic regression model, Exposure = covariates |
| **Confounding adjustment method** | ***Name method and provide relevant details, e.g. bivariate, multivariable, propensity score matching (specify matching algorithm ratio and caliper), propensity score weighting (specify weight formula, trimming, truncation), propensity score stratification (specify strata definition), other.*** |
|  | We will use fine stratification on the propensity score to adjust for confounding. The steps to do this include:   - Exclude pregnancies from the nonoverlapping regions of the PS distributions - Form 50 equally sized strata based on PS distribution in exposed women - Give exposed patients weight = 1 - Give comparator patients weight = (N_exposed_ in PS stratum i/N_totalexposed_) / (N_reference_ in PS stratum i/N_total reference_) |
| **Missing data methods** | ***Name method and provide relevant details, e.g. missing indicators, complete case, last value carried forward, multiple imputation (specify model/variables), other.*** |
|  | We will assume that if no clinical codes indicative of a condition or procedure are in the claims data, that the patient did not have the condition or procedure. |
| **Subgroup Analyses** | ***List all subgroups*** |
|  | Not applicable. |

#### Table 11. Sensitivity analyses – rationale, strengths and limitations

|  | **What is being varied? How?** | **Why?  (What do you expect to learn?)** | **Strengths of the sensitivity analysis compared to the primary** | **Limitations of the sensitivity analysis compared to the primary** |
| --- | --- | --- | --- | --- |
| Sensitivity analysis 1 | Redefined exposure as having at least 2 pharmacy dispensing records for topiramate during the 1^st^ trimester | To evaluate the effect of exposure misclassification. Non-differential misclassification tends to bias results towards the null. | The assumption is that women with at least 2 prescriptions filled in 3 months are more likely to have adhered to at least the first one | Reduces sample size of exposed patients |
| Sensitivity analysis 2 | Redefined the outcome on the basis of infant claims only and extended infant follow-up to 1 year | To evaluate the effect of outcome misclassification. Non-differential misclassification tends to bias results towards the null. | Potentially captures more late coded outcomes | Reduces sample size because fewer infants have lengthy follow up |

- 1. Data sources

#### 7.6.1 Context and rationale for data sources

**Reason for selection:** Given the relatively low incidence of oral clefts, evidence must necessarily come from large observational studies. The Medicaid Analytic eXtract (MAX) and Transformed Medicaid Statistical Information System Analytic Files (TAF) include data for enrollees in Medicaid and in the Children's Health Insurance Program (CHIP) in all 50 states and the District of Columbia. In 2018, 36.3 million children were enrolled in Medicaid and 9.6 million in CHIP. Children 0-19 years of age raised in families earning 138% or less of the federal poverty level (FPL) per year are eligible for Medicaid. The CHIP program expands coverage to children who have lower family incomes but who fall outside the Medicaid eligibility window.

**Strengths of data source(s):** The data source contains longitudinal, date stamped information on patient enrolment, demographics, in and outpatient diagnoses, procedures, admission and discharge dates, and medication dispensing that can be used to capture exposure, key inclusion-exclusion criteria, outcome, and covariates. The data are derived from claims for services received by the patient.

**Limitations of data source(s):** There is limited clinical information. The data sources have limited or no laboratory values, vital signs, clinical notes or reports to capture study parameters. Inpatient medication dispensing data are unavailable. Conditions must be diagnosed to appear on claims. Services that are not covered by insurance do not appear in the data.

**Data source provenance/curation:** The selected data sources are widely used for research and the data holders provide thorough documentation of data contents, assumptions and limitations.

#### Table 12. Metadata about data sources and software

|  | **Data 1** |
| --- | --- |
| **Data Source(s):** | Nationwide Medicaid Analytic eXtract (MAX) |
| **Study Period:** | 2000-2010 |
| **Eligible Cohort Entry Period:** | 2000-2010 |
| **Data Version (or date of last update):** | Not reported |
| **Data sampling/extraction criteria:** | Nationwide MAX |
| **Type(s) of data:** | Administrative claims |
| **Data linkage:** | No external data linkage. Mothers and infants were linked internal MAX |
| **Conversion to CDM*:** | Not reported |
| **Software for data management:** | Not reported (SAS version 9.3 used for data analysis) |

*CDM = Common Data Model

- 1. Data management

The research team operates a secure, state-of-the-art, computing facility. The computer cluster is Linux-based and offers SAS 9.4, Stata 15.1, and R. The data center is a secure facility that houses both our computing environment as well as clinical systems and electronic medical records for several large hospitals in Boston, MA. Entry into the computer room requires passing through staffed building security, a successful palm scan, and then passing through staffed computer room security. The research machines are connected to the networking backbone with 10 gigabit-per-second fiber links. Network security is overseen by Information Security, who apply the same standards used for the hospitals electronic medical records systems to the research teams data. All data are transmitted to programmers' workstations in an encrypted state. Backups are created using 256-bit AES encryption, the current Department of Defense standard for data security, and are stored in a locked facility.

The Data Manager will securely download data from the vendors to Division servers via secure SFTP. Data location, contents and data use agreements will be logged. Access to the servers are strictly controlled via physical and technical means to ensure that only individuals with proper clearance and authorization are able to access research data. When a project is closed, the research data are destroyed using a “shred” secure file deletion tool to ensure that sensitive data can never be retrieved.

ata cleaning and descriptive analyses were performed in IBM

SPSS (version 23). Regression models were developed in Stata Corp.

STATA (version 14.1).

ata cleaning and descriptive analyses were performed in IBM

SPSS (version 23). Regression models were developed in Stata Corp.

STATA (version 14.1).

Data cleaning will be performed with SAS 9.4. Feasibility, descriptive and regression analyses will be conducted with the Aetion Evidence Platform® (2021) v4.2.

- 1. Quality control

The data sources have been through extensive quality control procedures and documentation of the data is provided by the vendor. When new data is received from a vendor, the research group has an internal quality check process which includes assessment of reliability and conformance to expected plausible values. Issues are flagged for review by the data quality team and resolved with documentation of decisions made to clean the data before it is released to the research team to conduct studies.

- 1. Study size and feasibility

This protocol describes an attempt to reproduce the findings of another study using the same data source. The original study had a sample size of 2,425 topiramate exposed, 1,322,955 unexposed, and 2,796 lamotrigine exposed.^1^ This provided sufficient power to detect a nearly 3-fold elevation in risk for topiramate compared to non-exposure with 95% confidence intervals excluding the null.

1. Limitation of the methods

There are several potential limitations with the methods specified in this protocol.

1. The data were not collected for research and some important variables may not be collected or will be measured imperfectly
   1. We have selected validated algorithms when possible
   2. We have created proxies for important variables that are not directly captured in the data to reduce confounding by unmeasured factors
2. There will not be randomization
   1. We have emulated the design of a target trial
   2. We have balanced compared groups on important risk factors for the outcome(s)
3. On treatment follow up may be short in real-world practice, there is potential for informative censoring
   1. The results may not capture efficacy of long-term treatment but can measure effectiveness in populations as they are actually treated.
   2. We will do sensitivity analyses regarding reasons for censoring and incorporating censoring weights
4. Protection of human subjects

The study proposal has been reviewed and approved by the ABC organization IRB to ensure ethical treatment of human subjects as well as privacy protections (HIPAA). The proposed study is observational research that makes secondary use of data collected as part of routine care and does not involve any intervention, alteration in standard clinical care or use of any procedure in patients. Therefore, there will be no adverse events related to the study itself. No patients will be contacted for any of the proposed studies. Prior to our acquisition of the data, all personal identifiers will be encrypted. This encryption minimizes the risk of patient reidentification in the unlikely event of a breach in data security. The institution’s uses standard-issue virus protection software and access to data is controlled through the use of individual passwords known only to study staff. Study staff are required to complete human-subject protection education requirements as well as HIPAA training prior to being allowed to work on any data and are regularly re-certificated. As a further layer of privacy protection, cell sizes less than 11 will be suppressed in tables produced from Medicaid data, in accordance with the data use agreement.

1. Reporting of adverse events

The proposed study is observational research that makes secondary use of data collected as part of routine care and does not involve any intervention or alteration in clinical care. Therefore, reporting of adverse events related to this study is not applicable. Safety evaluations for this study are limited to the specified safety outcomes stated in section 4.4.2.

1. References

1. Hernandez-Diaz S, Huybrechts KF, Desai RJ, et al. Topiramate use early in pregnancy and the risk of oral clefts: A pregnancy cohort study. *Neurology*. Jan 23 2018;90(4):e342-e351. doi:10.1212/WNL.0000000000004857

2. Palmsten K, Huybrechts KF, Mogun H, et al. Harnessing the Medicaid Analytic eXtract (MAX) to Evaluate Medications in Pregnancy: Design Considerations. *PloS one*. 2013;8(6):e67405. doi:10.1371/journal.pone.0067405

3. Tikkanen J, Heinonen OP. Risk factors for ventricular septal defect in Finland. *Public Health*. Mar 1991;105(2):99-112. doi:10.1016/s0033-3506(05)80283-5

4. Bateman BT, Mhyre JM, Hernandez-Diaz S, et al. Development of a comorbidity index for use in obstetric patients. *Obstetrics and gynecology*. Nov 2013;122(5):957-965. doi:10.1097/AOG.0b013e3182a603bb

5. Schneeweiss S, Seeger JD, Maclure M, Wang PS, Avorn J, Glynn RJ. Performance of comorbidity scores to control for confounding in epidemiologic studies using claims data. *American journal of epidemiology*. Nov 1 2001;154(9):854-64.

1. Appendices

See excel files.

Appendix A - study population entry criteria (exposure)

Appendix B - drug, diagnosis and procedure based inclusion/exclusion criteria

Appendix C - drug, diagnosis and procedure based covariates

Appendix D - outcome

Appendix E - care setting
