# Supplementary material for: HARmonized Protocol Template to Enhance Reproducibility of hypothesis evaluating real‐world evidence studies on treatment effects: A good practices report of a joint ISPE/ISPOR task force
Source: Pharmacoepidemiol Drug Saf. 2022 Oct 10;32(1):44–55. doi: 10.1002/pds.5507 (PMC9771861; doi:10.1002/pds.5507)
Supplement: Supplementary file 3 — Appendix 3. Example use cases. [file PDS-32-44-s004.zip › Appendix 3/Example 5 SCCS effectiveness.docx]

# 1. Title Page

DISCLAIMER: This protocol is based on the research question and design from a published study^1^, but there may be differences in exact scientific decisions. Some of the information needed to complete the protocol template was not available from the publication. For the purposes of populating the protocol template, the subgroup working on this example use case made fictional or reasonable choices that may not be reflective of the actual scientific decisions made by the original investigators. Abbreviated entries are provided to illustrate how to use the protocol template, however appendices were not prepared for this example protocol.

| Title | Reproduction of a Study on the Effectiveness of Palivizumab against Respiratory Syncytial Virus Case Series Analysis |
| --- | --- |
| Research question & Objectives | To reproduce the findings of real-world effectiveness of palivizumab immunoprophylaxis against respiratory syncytial virus (RSV)-confirmed infection before age 2 years in a population-cohort of high-risk infants |
| Protocol version | V1.0 |
| Last update date | 7/7/2021 |
| Contributors | **Primary investigator contact information:**  Jane Doe  **Contributor names:**  John Smith |
| Study registration | **Site:** Real World Evidence Registry: https://osf.io/registries/rwe/discover  **Identifier:** doi:abc123 |
| Sponsor | **Organization:** ABC Institutional Review Board  **Contact:** IRB123 |
| Conflict of interest | None |

Table of contents

[1. Title Page 1](#_Toc102631527)

[2. Abstract 4](#_Toc102631528)

[3. Amendments and updates 4](#_Toc102631529)

[4. Milestones 5](#_Toc102631530)

[Table 1 Milestones 5](#_Toc102631531)

[5. Rationale and background 5](#_Toc102631532)

[6. Research question and objectives 5](#_Toc102631533)

[Table 2 Primary and secondary research questions and objective 5](#_Toc102631534)

[7. Research methods 6](#_Toc102631535)

[7.1. Study design 6](#_Toc102631536)

[7.2. Study design diagram 7](#_Toc102631537)

[7.3. Setting 7](#_Toc102631538)

[7.3.1 Context and rationale for definition of time 0 (or other primary time anchors) for entry to the study population 7](#_Toc102631539)

[Table 3 Operational Definition of Time 0 (index date) or other primary time anchors 8](#_Toc102631540)

[7.3.2 Context and rationale for study inclusion criteria: 8](#_Toc102631541)

[Table 4. Operational Definitions of Inclusion Criteria 8](#_Toc102631542)

[7.3.3 Context and rationale for study exclusion criteria 8](#_Toc102631543)

[Table 5. Operational Definitions of Exclusion Criteria 9](#_Toc102631544)

[7.4. Variables 9](#_Toc102631545)

[7.4.1 Context and rationale for exposure(s) of interest 9](#_Toc102631546)

[Table 6. Operational Definitions of Exposure 9](#_Toc102631547)

[7.4.2 Context and rationale for outcome(s) of interest 10](#_Toc102631548)

[Table 7. Operational Definitions of Outcome 10](#_Toc102631549)

[7.4.3 Context and rationale for follow up 10](#_Toc102631550)

[Table 8. Operational Definitions of Follow Up 11](#_Toc102631551)

[7.4.4 Context and rationale for covariates (confounding variables and effect modifiers, e.g. risk factors, comorbidities, comedications) 11](#_Toc102631552)

[Table 9. Operational Definitions of Covariates 11](#_Toc102631553)

[7.5. Data analysis 12](#_Toc102631554)

[7.5.1 Context and rationale for analysis plan 12](#_Toc102631555)

[Table 10. Primary, secondary, and subgroup analysis specification 12](#_Toc102631556)

[Table 11. Sensitivity analyses – rationale, strengths and limitations 13](#_Toc102631557)

[7.6. Data sources 13](#_Toc102631558)

[7.6.1 Context and rationale for data sources 13](#_Toc102631559)

[Table 12. Metadata about data sources and software 14](#_Toc102631560)

[7.7. Data management 15](#_Toc102631561)

[7.8. Quality control 15](#_Toc102631562)

[7.9. Study size and feasibility 15](#_Toc102631563)

[Table 13. Power and sample size 16](#_Toc102631564)

[8. Limitation of the methods 16](#_Toc102631565)

[9. Protection of human subjects 16](#_Toc102631566)

[10. Reporting of adverse events 17](#_Toc102631567)

[11. References 17](#_Toc102631568)

[12. Appendices 17](#_Toc102631569)

2. Abstract

Palivizumab is an injectable monoclonal antibody. It is given via monthly intramuscular injections to high-risk populations to prevent RSV-confirmed hospitalization, with risk reductions of 45%-55%. Palivizumab is licensed in Australia for use in high-risk infants, however there is no uniform national guideline or policy governing palivizumab use.

This study will evaluate the reproducibility of previously published findings on the effectiveness of palivizumab in high-risk infants.

The study cohort will be identified using the WA NICU database. Maternal and infant demographic information will be obtained by linking the study cohort to the Midwives’ Notifications System. Deaths will be identified by linking the study cohort to registered deaths. Exposure to palivizumab will be identified by linking to hospital-based and state-based pharmacy dispensing datasets. Hospitalizations will be identified from the Hospital Morbidity Data Collection. The outcome of RSV will be identified from the PathWest Laboratory Database of routine pathology testing. will be extracted and probabilistically linked by the Western Australian Data Linkage Branch using demographic identifiers. All babies in the NICU will be identified because there are only 2 tertiary hospitals with NICU in WA. PathWest Laboratory Medicine WA is the only public pathology provider in WA. Rich data on maternal and infant characteristics will be available from the electronic health records. Each data provider has generated documentation about the data content and characteristics (see appendices).

3. Amendments and updates

| **Version date** | **Version number** | **Section of protocol** | **Amendment or update** | **Reason** |
| --- | --- | --- | --- | --- |
| 7/7/2021 | 1 | First draft | n/a | n/a |

1. Milestones

#### Table 1 Milestones

| **Milestone** | **Date** |
| --- | --- |
| Feasibility counts | 3/1/2021 |
| Draft 1 of protocol complete | 7/7/2021 |
| Registration of protocol | 7/7/2021 |
| Study progress report 1 | 1/1/2022 |
| Study progress report 2 | 6/30/2022 |
| Final report of study results | 12/31/2022 |

1. Rationale and background

**What is known about the condition:** In 2015 there were over 30 million episodes of acute lower respiratory episodes, over 3 million hospitalisations, and 60,000 in hospital deaths in children age under 5 that were attributed to respiratory syncytial virus (RSV).^2^

**What is known about the exposure of interest:** Palivizumab is an injectable monoclonal antibody. It is given via monthly intramuscular injections to high-risk populations to prevent RSV-confirmed hospitalization, with risk reductions of 45%-55%.^3^

**Gaps in knowledge:** Palivizumab is licensed in Australia for use in high-risk infants, however there is no uniform national guideline or policy governing palivizumab use.

**What is the expected contribution of this study?** Demonstrated reproducibility of the findings on effectiveness of palivizumab in high risk infants.

1. Research question and objectives

#### Table 2 Primary and secondary research questions and objective

1. **Primary research question and objective**

| **Objective:** | To replicate the findings of a self-controlled case-series (SCCS) study that estimated the effectiveness of a multiple dose course of palivizumab for preventing RSV in infants discharged from the neonatal intensive care unit (NICU).^1^ |
| --- | --- |
| **Hypothesis:** | Palivizumab reduces risk of RSV. |
| **Population *(mention key inclusion-exclusion criteria):*** | All infants admitted to either of the 2 tertiary NICUs at King Edward Memorial Hospital and Princess Margaret Hospital immediately after birth from January 1, 2002-December 31, 2013. The Western Australia (WA) NICU database will be used to identify the study cohort. |
| **Exposure:** | Palivizumab doses administered 2002-2015 will be identified from a collection of historical and current hospital-based and state-based pharmacy dispensing datasets, stratified by the number of doses and included both inpatients and outpatients. Exposed time includes the 28 days following administration of palivizumab. |
| **Comparator:** | Unexposed time |
| **Outcome:** | Positive laboratory test result for RSV before age 2 years. This includes positive RSV tests from respiratory specimens with standard detection methods as per the established protocol. |
| **Time *(when follow up begins and ends):*** | All time from age 0 to 2 years |
| **Setting:** | NICU, outpatient, inpatient, pharmacy |
| **Main measure of effect:** | Incidence rate ratio (IRR) |

1. **Secondary research question 1 and objective**

n/a

1. Research methods
   1. Study design

**Research design (e.g. cohort, case-control, etc.):** Self-controlled case series (SCCS)

**Rationale for study design choice:** This method has previously been used to investigate adverse events following vaccination. The design will mitigate residual confounding by indication due to targeting palivizumab to those at greatest risk of RSV.

- 1. Study design diagram


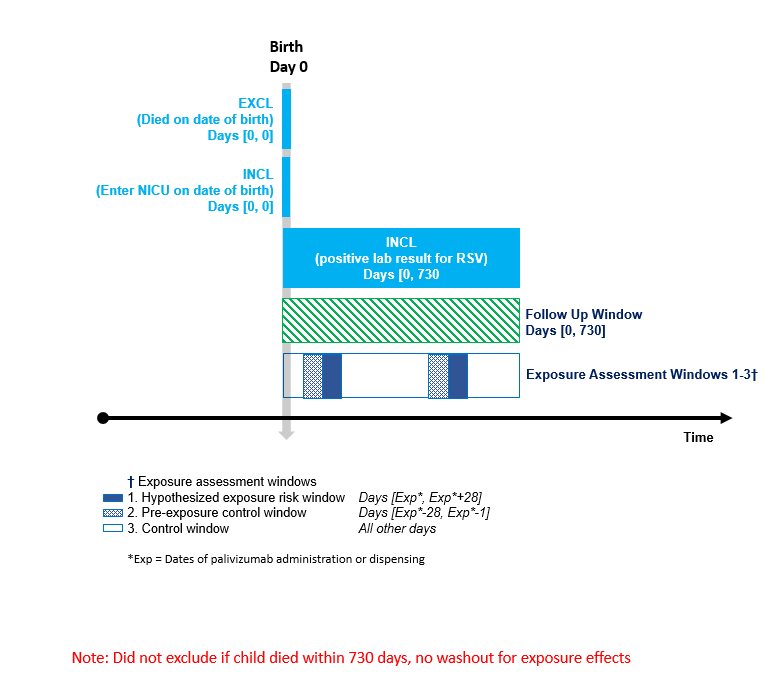


This design diagram illustrates a patient with 2 separate palivizumab exposures. There could be fewer or more exposures for any given child included in the analysis, so long as it occurs within the follow up window from birth to 730 days.

- 1. Setting

#### 7.3.1 Context and rationale for definition of time 0 (or other primary time anchors) for entry to the study population

Children enter the cohort on their date of birth for this self-controlled case-series analysis.

#### Table 3 Operational Definition of Time 0 (index date) or other primary time anchors

| **Study population name(s)** | **Time Anchor Description**  **(e.g. time 0)** | **Number of entries** | **Type of entry** | **Washout window** | **Care Setting^1^** | **Code Type^2^** | **Diagnosis position** | **Incident with respect to…** | **Measurement characteristics/validation** | **Source of algorithm** |
| --- | --- | --- | --- | --- | --- | --- | --- | --- | --- | --- |
| newborn NICU | birth | 1 | incident | n/a | IP | n/a | n/a | n/a | No validation study | Presence in WA NICU database |

^1^ IP = inpatient, OP = outpatient, ED = emergency department, OT = other, n/a = not applicable

^2^See appendix for listing of clinical codes for each study parameter

#### 7.3.2 Context and rationale for study inclusion criteria:

Children are required to enter the NICU on their date of birth because the study will focus on children at high risk of RSV. They are included in the cohort if they have a positive RSV test at some point within the first 2 years of life. This requirement is because the self-controlled case series design will compare exposed and unexposed person-time among children with the outcome of interest.

#### Table 4. Operational Definitions of Inclusion Criteria

| **Criterion** | **Details** | **Order of application** | **Assessment window** | **Care Settings¹** | **Code Type^2^** | **Diagnosis position^3^** | **Applied to study populations:** | **Measurement characteristics/validation** | **Source for algorithm** |
| --- | --- | --- | --- | --- | --- | --- | --- | --- | --- |
| *NICU* | *Presence in WA NICU database on date of birth* | *n/a* | *[0, 0]* | *IP* | *n/a* | *n/a* | newborn NICU | No validation study | *WA NICU database* |
| *Positive RSV* | *Lab test* | *n/a* | *[0, 730]* | *IP, OP, ED, OT* | *n/a* | *n/a* | newborn NICU | No validation study | *See Lim, et al.^4^* |

^1^ IP = inpatient, OP = outpatient, ED = emergency department, OT = other, n/a = not applicable

^2^ See appendix for listing of clinical codes for each study parameter

^3^ Specify whether a diagnosis code is required to be in the primary position (main reason for encounter)

#### 7.3.3 Context and rationale for study exclusion criteria

Children will be excluded if they died on their date of birth because they would have no opportunity for either exposure to palivizumab or development of RSV.

#### Table 5. Operational Definitions of Exclusion Criteria

| **Criterion** | **Details** | **Order of application** | **Assessment window** | **Care Settings¹** | **Code Type^2^** | **Diagnosis position^3^** | **Applied to study populations:** | **Measurement characteristics/validation** | **Source for algorithm** |
| --- | --- | --- | --- | --- | --- | --- | --- | --- | --- |
| *Death on birth date* | *Died on birth date* | *n/a* | *[0, 0]* | *IP* | *n/a* | *n/a* | Newborn NICU | No validation study | WA NICU database, death register |

^1^ IP = inpatient, OP = outpatient, ED = emergency department, OT = other, n/a = not applicable

^2^ See appendix for listing of clinical codes for each study parameter

^3^ Specify whether a diagnosis code is required to be in the primary position (main reason for encounter)

- 1. Variables

#### 7.4.1 Context and rationale for exposure(s) of interest

The exposure risk windows for palivizumab will be defined as the dispensing date + 28 days. The 28-day window was defined based on the expected duration of effect of palivizumab after administration. There will be a pre-exposure window of 28 days prior to each dispensing to address healthy user effects. All other person-time up until 730 days after birth will be considered unexposed.

**Algorithm to define duration of exposure effect:**

If a refill occurs before the end of days supply dispensed, the overlapping days are ignored. A 28-day window of “exposed time” is added to each dispensation/administration date for palivizumab.

#### Table 6. Operational Definitions of Exposure

| **Exposure group name(s)** | **Detail** | **Washout window** | **Assessment Window** | **Care Setting^1^** | **Code Type^2^** | **Diagnosis position^3^** | **Applied to study populations:** | **Incident with respect to…** | **Measurement characteristics/**  **validation** | **Source of algorithm** |
| --- | --- | --- | --- | --- | --- | --- | --- | --- | --- | --- |
| *Palivizumab risk window* |  | *n/a* | *[exp*, exp*+28]* | *IP or OP* | *ATC* | *n/a* | Newborn NICU | *palivizumab* | No validation study | *Investigator defined* |
| *Pre-exposed window* |  | *n/a* | *[exp*-28, exp*-1]* | *IP or OP* | *ATC* | *n/a* | Newborn NICU | *Drug A or B*  *(any formulation)* | No validation study | *n/a* |
| *Unexposed window* |  | *n/a* | *All other days* | *IP or OP* | *ATC* | *n/a* | Newborn NICU | *Drug A or B*  *(any formulation)* | No validation study | *n/a* |

^1^ IP = inpatient, OP = outpatient, ED = emergency department, OT = other, n/a = not applicable

^2^ See appendix for listing of clinical codes for each study parameter

^3^ Specify whether a diagnosis code is required to be in the primary position (main reason for encounter)

#### 7.4.2 Context and rationale for outcome(s) of interest

The first positive RSV lab test will be included as an outcome. Subsequent positive tests will not be counted because they may not be independent recurrent events.

#### Table 7. Operational Definitions of Outcome

| **Outcome name** | **Details** | **Primary outcome?** | **Type of outcome** | **Washout window** | **Care Settings¹** | **Code Type^2^** | **Diagnosis Position^3^** | **Applied to study populations:** | **Outcome measurement characteristics/**  **validation** | **Source of algorithm** |
| --- | --- | --- | --- | --- | --- | --- | --- | --- | --- | --- |
| RSV positive lab |  | Yes | Incident rate | Only first included,  From day 0 | IP, OP, ED, OT | n/a | n/a | newborn NICU | Positive laboratory  test result for RSV before age 2 years  While 62% (n = 26 893) of laboratory-confirmed admissions received respiratory infection diagnosis codes, 38% (n = 16 734) had other diagnoses, notably viral infection of unspecified sites^4^ | *See Lim, et al.^4^* |

^1^ IP = inpatient, OP = outpatient, ED = emergency department, OT = other, n/a = not applicable

^2^ See appendix for listing of clinical codes for each study parameter

^3^ Specify whether a diagnosis code is required to be in the primary position (main reason for encounter)

#### 7.4.3 Context and rationale for follow up

The follow up window is defined as the first 2 years of life for the cohort of children that meet inclusion and exclusion criteria. Exposure status is time varying within this follow up window. Analyses will condition on the individual because this is a self-controlled case series study.

#### Table 8. Operational Definitions of Follow Up

|  |  |  |  |
| --- | --- | --- | --- |
| **Follow up start** | Birth |  |  |
| **Follow up end^1^** | **Select all that apply** |  | **Specify** |
| **Date of outcome** | No |  | See Table 5 |
| **Date of death** | No |  | Death registrations identified any deaths in the cohort 2002-2015. |
| **End of observation in data** | No |  |  |
| **Day X following index date**  *(specify day)* | Yes |  | Day 730 |
| **End of study period**  (specify date) | Yes |  | 31-Dec-15 |
| **End of exposure**  *(specify operational details,*  *e.g. stockpiling algorithm, grace period)* | No |  |  |
| **Date of add to/switch from exposure**  *(specify algorithm)* | No |  | n/a |
| **Other date** *(specify)* | No |  | n/a |

^1^ Follow up ends at the first occurrence of any of the selected criteria that end follow up.

#### 7.4.4 Context and rationale for covariates (confounding variables and effect modifiers, e.g. risk factors, comorbidities, comedications)

Covariates will include variables that were expected a priori to be related to both palivizumab use and RSV detection to mitigate against confounding.

#### Table 9. Operational Definitions of Covariates

| **Characteristic** | **Details** | **Type of variable** | **Assessment window** | **Care Settings¹** | **Code Type^2^** | **Diagnosis Position^3^** | **Applied to study populations:** | **Measurement characteristics/validation** | **Source for algorithm** |
| --- | --- | --- | --- | --- | --- | --- | --- | --- | --- |
| Age | Age of child in days on each day of followup. 0-2 months,  3-5 months, 6-11 months, and 12-23 months) | Continuous, time varying | [0, 730] | n/a | n/a | n/a | newborn NICU | n/a | *n/a* |
| Season | RSV seasonal  period, defined as April-October in any given year | Categorical, time varying | [0, 730] | n/a | n/a | n/a | newborn NICU | No validation study | *n/a* |

^1^ IP = inpatient, OP = outpatient, ED = emergency department, OT = other, n/a = not applicable

^2^ See appendix for listing of clinical codes for each study parameter

^3^ Specify whether a diagnosis code is required to be in the primary position (main reason for encounter)

- 1. Data analysis

#### 7.5.1 Context and rationale for analysis plan

A self-controlled case series design will be used to avoid bias from confounding by indication.

#### Table 10. Primary, secondary, and subgroup analysis specification

1. **Primary analysis**

| **Hypothesis:** | Palivizumab is associated with a decreased risk of RSV |
| --- | --- |
| **Exposure contrast:** | Palivizumab exposed time vs unexposed time |
| **Outcome:** | RSV |
| **Analytic software:** | STATA 15.1 |
| **Model(s):**  ***(provide details or code)*** | *Outcome model:* conditional Poisson (adjustment for age and RSV season ) |
| **Confounding adjustment method** | ***Name method and provide relevant details, e.g. bivariate, multivariable, propensity score matching (specify matching algorithm ratio and caliper), propensity score weighting (specify weight formula, trimming, truncation), propensity score stratification (specify strata definition), other.*** |
|  | We will use a self-controlled model with partitions by age and RSV season. The self-controlled analysis will remove confounding from characteristics that do not vary over time within individual. The partitions by age and season will adjust for confounding by these factors. |
| **Missing data methods** | ***Name method and provide relevant details, e.g. missing indicators, complete case, last value carried forward, multiple imputation (specify model/variables), other.*** |
|  | Only patients with measured outcome, age, and RSV season contributed to the analysis. Patients who cannot be linked to each of the data sources will be excluded. |
| **Subgroup Analyses** | ***List all subgroups*** |
|  | Not applicable. |

#### Table 11. Sensitivity analyses – rationale, strengths and limitations

|  | **What is being varied? How?** | **Why?  (What do you expect to learn?)** | **Strengths of the sensitivity analysis compared to the primary** | **Limitations of the sensitivity analysis compared to the primary** |
| --- | --- | --- | --- | --- |
| Sensitivity Analysis 1 | Primary analysis restricted by year of birth, restricted to births during 2010-2013 | Because prevalence of palivizumab use has increased in NICU in more recent years. | Exposed patients in time restricted population is reflective of the general NICU population instead of an unusual population that is treated before palivizumab was commonly used in NICU. | Smaller sample size; power is lower |
| Sensitivity Analysis 2 | Palivizumab doses were all grouped together, ignoring which dose it was (e.g., 1^st^ dose received, 2^nd^ dose received, etc.) | Because of small numbers | Increases sample size, leading to more power to detect an effect | Unable to evaluate dose effects |

- 1. Data sources

#### 7.6.1 Context and rationale for data sources

**Reason for selection:** The study cohort will be identified using the WA NICU database. Maternal and infant demographic information will be obtained by linking the study cohort to the Midwives’ Notifications System. Deaths will be identified by linking the study cohort to registered deaths. Exposure to palivizumab will be identified by linking to hospital-based and state-based pharmacy dispensing datasets. Hospitalizations will be identified from the Hospital Morbidity Data Collection. The outcome of RSV will be identified from the PathWest Laboratory Database of routine pathology testing. will be extracted and probabilistically linked by the Western Australian Data Linkage Branch using demographic identifiers as described in Holman et al.^5^

**Strengths of data source(s):** All babies in the NICU will be identified because there are only 2 tertiary hospitals with NICU in WA. PathWest Laboratory Medicine WA is the only public pathology provider in WA. Rich data on maternal and infant characteristics will be available rom the electronic health records.

**Limitations of data source(s):** The data from numerous sources will be probabilistically linked, and there may be error in the linkage.

**Data source provenance/curation:** Each data provider provided documentation about the data content and characteristics (see appendices).

#### Table 12. Metadata about data sources and software

|  | **Data 1** | **Data 2** | **Data 3** |
| --- | --- | --- | --- |
| **Data Source(s):** | WA NICU database | Midwives’ Notifications System | Death registrations |
| **Study Period:** | January 1, 2002 - December 31, 2013 | January 1, 2002 - December 31, 2013 | 2002-2015 |
| **Eligible Cohort Entry Period:** | January 1, 2002 - December 31, 2013 | January 1, 2002 - December 31, 2013 | n/a |
| **Data Version (or date of last update):** | TBD | TBD | TBD |
| **Data sampling/extraction criteria:** | Cohort of newborns in NICU – includes all infants admitted to either of the 2 tertiary NICUs at King Edward Memorial Hospital and Princess Margaret Hospital immediately after birth | All data linkable to cohort of newborns in NICU | All deaths linkable to cohort of newborns in NICU |
| **Type(s) of data:** | Administrative claims | Maternal and infant demographic information | Death data |
| **Data linkage:** | Yes (Data were extracted and probabilistically linked by the Western Australian Data Linkage  Branch using a series of demographic identifiers) | Yes (Data were extracted and probabilistically linked by the Western Australian Data Linkage  Branch using a series of demographic identifiers) | Yes (Data were extracted and probabilistically linked by the Western Australian Data Linkage  Branch using a series of demographic identifiers) |
| **Conversion to CDM*:** | n/a | n/a | n/a |
| **Software for data management:** | Stata v 15.1 | Stata v 15.1 | Stata v 15.1 |

|  | **Data 4** | **Data 5** | **Data 6** |
| --- | --- | --- | --- |
| **Data Source(s):** | Hospital Morbidity Data Collection | PathWest Laboratory Database | Historical and current hospital-based and state-based pharmacy dispensing datasets |
| **Study Period:** | January 2002-June 2015 | January 2002-December 2015 | January 1, 2002 - December 31, 2015 |
| **Eligible Cohort Entry Period:** |  |  |  |
| **Data Version (or date of last update):** | TBD | TBD | TBD |
| **Data sampling/extraction criteria:** | All data linkable to cohort of newborns in NICU | All data linkable to cohort of newborns in NICU | All data linkable to cohort of newborns in NICU |
| **Type(s) of data:** | Administrative claims | Lab data | Pharmacy data |
| **Data linkage:** | Yes (Data were extracted and probabilistically linked by the Western Australian Data Linkage  Branch using a series of demographic identifiers) | Yes (Data were extracted and probabilistically linked by the Western Australian Data Linkage  Branch using a series of demographic identifiers) | Yes (Data were extracted and probabilistically linked by the Western Australian Data Linkage  Branch using a series of demographic identifiers) |
| **Conversion to CDM*:** | n/a | n/a | n/a |
| **Software for data management:** | Stata v 15.1 | Stata v 15.1 | Stata v 15.1 |

*CDM = Common Data Model

- 1. Data management

The research team operates a secure, state-of-the-art, computing facility. The computer cluster is Linux-based and offers SAS 9.4, Stata 15.1, and R. The data center is a secure facility that houses both our computing environment as well as clinical systems and electronic medical records for several large hospitals in WA. Entry into the computer room requires passing through staffed building security, a successful palm scan, and then passing through staffed computer room security. The research machines are connected to the networking backbone with 10 gigabit-per-second fiber links. Network security is overseen by Information Security, who apply the same standards used for the hospitals electronic medical records systems to the research teams data. All data are transmitted to programmers' workstations in an encrypted state. Backups are created using 256-bit AES encryption, the current Department of Defense standard for data security, and are stored in a locked facility.

The Data Manager will securely download data from the various sources to the servers in the computing cluster via secure SFTP. Data location, contents and data use agreements will be logged. Access to the servers are strictly controlled via physical and technical means to ensure that only individuals with proper clearance and authorization are able to access research data. When a project is closed, the research data are destroyed using a “shred” secure file deletion tool to ensure that sensitive data can never be retrieved.

ata cleaning and descriptive analyses were performed in IBM

SPSS (version 23). Regression models were developed in Stata Corp.

STATA (version 14.1).

ata cleaning and descriptive analyses were performed in IBM

SPSS (version 23). Regression models were developed in Stata Corp.

STATA (version 14.1).

Data cleaning, descriptive and regression analyses will be performed with STATA (version 15.1).

- 1. Quality control

The data sources have been through extensive quality control procedures and documentation of the data and collection procedures is provided (see appendices). The research group has an internal quality check process which includes assessment of reliability and conformance to expected plausible values. Issues are flagged for review by the data quality team and resolved with documentation of decisions made to clean the data (see appendices).

- 1. Study size and feasibility

Based on a prior publication looking at this question with this data^1^, we expect to identify 1500 infants with laboratory confirmed RSV during the first 2 years of life with about 2.5% exposed to pavlizumab, we expect more than 90% power to detect a 75% reduction in RSV associated with pavlizumab exposure. Power calculation based on formula from Musonda et al.^6^

#### Table 13. Power and sample size

| Power | 90% |
| --- | --- |
| Type 1 error | 5% |
| Relative incidence | 0.25 |
| Ratio of risk to observation length | 0.038 |
| Sample size | 277 |

1. Limitation of the methods

There are several potential limitations with the methods specified in this protocol.

1. Some important variables may not be collected or will be measured imperfectly
   1. Timing is important for SCCS designs, however the data provides only dispensing data which may not always be the same as the date of administration
   2. The outcome definition is very specific but may miss RSV cases, especially because not all infants are tested
2. The SCCS design requires some assumptions^7^:
   1. Developing RSV will not affect future palivizumab exposure or increase mortality such that observation windows are censored
   2. The event rate of RSV is constant within compared time windows (with the exception of the effect of exposure)
   3. RSV must be rare or recurrences independent
3. Protection of human subjects

The study proposal has been reviewed and approved by the ABC ethics review board to ensure ethical treatment of human subjects as well as privacy protections. The proposed study is observational research that makes secondary use of data collected as part of routine care as well as patient reported outcomes. The project does not involve any intervention, alteration in standard clinical care or use of any procedure in patients. Therefore, there will be no adverse events related to the study itself. All personal identifiers will be encrypted. This encryption minimizes the risk of patient reidentification in the unlikely event of a breach in data security. The institution’s uses standard-issue virus protection software and access to data is controlled through the use of individual passwords known only to study staff. Study staff are required to complete the ABC training prior to being allowed to work on any data and are regularly re-certificated. As a further layer of privacy protection, cell sizes less than 11 will be suppressed in results tables.

1. Reporting of adverse events

The proposed study is observational research that makes secondary use of data collected as part of routine care and does not involve any intervention or alteration in clinical care. Therefore, reporting of adverse events related to this study is not applicable. Safety evaluations for this study are limited to the specified safety outcomes stated in section 4.4.2.

1. References

1. Moore HC, de Klerk N, Richmond PC, et al. Effectiveness of Palivizumab against Respiratory Syncytial Virus: Cohort and Case Series Analysis. *The Journal of pediatrics*. Nov 2019;214:121-127.e1. doi:10.1016/j.jpeds.2019.06.058

2. Shi T, McAllister DA, O'Brien KL, et al. Global, regional, and national disease burden estimates of acute lower respiratory infections due to respiratory syncytial virus in young children in 2015: a systematic review and modelling study. *The Lancet*. 2017/09/02/ 2017;390(10098):946-958. doi:<https://doi.org/10.1016/S0140-6736(17)30938-8>

3. Palivizumab, a humanized respiratory syncytial virus monoclonal antibody, reduces hospitalization from respiratory syncytial virus infection in high-risk infants. The IMpact-RSV Study Group. *Pediatrics*. Sep 1998;102(3 Pt 1):531-7.

4. Lim FJ, Blyth CC, Fathima P, de Klerk N, Moore HC. Record linkage study of the pathogen-specific burden of respiratory viruses in children. *Influenza Other Respir Viruses*. Nov 2017;11(6):502-510. doi:10.1111/irv.12508

5. Holman CD, Bass AJ, Rosman DL, et al. A decade of data linkage in Western Australia: strategic design, applications and benefits of the WA data linkage system. *Aust Health Rev*. Nov 2008;32(4):766-77. doi:10.1071/ah080766

6. Musonda P, Farrington CP, Whitaker HJ. Sample sizes for self-controlled case series studies. *Stat Med*. Aug 15 2006;25(15):2618-31. doi:10.1002/sim.2477

7. Petersen I, Douglas I, Whitaker H. Self controlled case series methods: an alternative to standard epidemiological study designs. *Bmj*. 2016;354:i4515. doi:10.1136/bmj.i4515

1. Appendices

See excel files.

Appendix A - study population entry criteria (exposure)

Appendix B - drug, diagnosis and procedure based inclusion/exclusion criteria

Appendix C - drug, diagnosis and procedure based covariates

Appendix D - outcomes

Appendix E - care setting

Appendix F – Data dictionaries and documentation

Appendix G – Quality control and data cleaning decisions
