# Supplementary material for: HARmonized Protocol Template to Enhance Reproducibility of hypothesis evaluating real‐world evidence studies on treatment effects: A good practices report of a joint ISPE/ISPOR task force
Source: Pharmacoepidemiol Drug Saf. 2022 Oct 10;32(1):44–55. doi: 10.1002/pds.5507 (PMC9771861; doi:10.1002/pds.5507)
Supplement: Supplementary file 4 — Appendix 4. Supporting Information. [file PDS-32-44-s002.docx]

Helper text to assist with using the harmonized protocol template is provided in green. This helper text may be deleted after filling out the template.

If a section is not applicable, enter “n/a”.

# 1. Title Page

| Title | <Text> |
| --- | --- |
| Research question & Objectives | <Text> |
| Protocol version | <Text> |
| Last update date | <Text> |
| Contributors | **Primary investigator contact information:**  <Text>  **Contributor names:**  <Text> |
| Study registration | **Site:** <Text>  **Identifier:** <Text> |
| Sponsor | **Organization:** <Text>  **Contact:** <Text> |
| Conflict of interest | <Text> |

Table of contents

[1. Title Page 1](#_Toc102631281)

[2. Abstract 3](#_Toc102631282)

[3. Amendments and updates 3](#_Toc102631283)

[4. Milestones 4](#_Toc102631284)

[Table 1 Milestones 4](#_Toc102631285)

[5. Rationale and background 4](#_Toc102631286)

[6. Research question and objectives 4](#_Toc102631287)

[Table 2 Primary and secondary research questions and objective 4](#_Toc102631288)

[7. Research methods 6](#_Toc102631289)

[7.1. Study design 6](#_Toc102631290)

[7.2. Study design diagram 6](#_Toc102631291)

[7.3. Setting 6](#_Toc102631292)

[7.3.1 Context and rationale for definition of time 0 (and other primary time anchors) for entry to the study population 6](#_Toc102631293)

[Table 3 Operational Definition of Time 0 (index date) and other primary time anchors 6](#_Toc102631294)

[7.3.2 Context and rationale for study inclusion criteria: 8](#_Toc102631295)

[Table 4. Operational Definitions of Inclusion Criteria 8](#_Toc102631296)

[7.3.3 Context and rationale for study exclusion criteria 9](#_Toc102631297)

[Table 5. Operational Definitions of Exclusion Criteria 9](#_Toc102631298)

[7.4. Variables 11](#_Toc102631299)

[7.4.1 Context and rationale for exposure(s) of interest 11](#_Toc102631300)

[Table 6. Operational Definitions of Exposure 11](#_Toc102631301)

[7.4.2 Context and rationale for outcome(s) of interest 12](#_Toc102631302)

[Table 7. Operational Definitions of Outcome 12](#_Toc102631303)

[7.4.3 Context and rationale for follow up 13](#_Toc102631304)

[Table 8. Operational Definitions of Follow Up 13](#_Toc102631305)

[7.4.4 Context and rationale for covariates (confounding variables and effect modifiers, e.g. risk factors, comorbidities, comedications) 14](#_Toc102631306)

[Table 9. Operational Definitions of Covariates 15](#_Toc102631307)

[7.5. Data analysis 16](#_Toc102631308)

[7.5.1 Context and rationale for analysis plan 16](#_Toc102631309)

[Table 10. Primary, secondary, and subgroup analysis specification 16](#_Toc102631310)

[Table 11. Sensitivity analyses – rationale, strengths and limitations 18](#_Toc102631311)

[7.6. Data sources 18](#_Toc102631312)

[7.6.1 Context and rationale for data sources 18](#_Toc102631313)

[Table 12. Metadata about data sources and software 18](#_Toc102631314)

[7.7. Data management 19](#_Toc102631315)

[7.8. Quality control 20](#_Toc102631316)

[7.9. Study size and feasibility 20](#_Toc102631317)

[Table 13. Power and sample size 20](#_Toc102631318)

[8. Limitation of the methods 20](#_Toc102631319)

[9. Protection of human subjects 20](#_Toc102631320)

[10. Reporting of adverse events 20](#_Toc102631321)

[11. References 21](#_Toc102631322)

[12. Appendices 21](#_Toc102631323)

2. Abstract

Include a brief description of the background, research question and objectives, study design, and data sources.

<Text>

3. Amendments and updates

Indicate amendments and updates to the study protocol in a table as indicated below. In the “reason” column, not whether the amendment occurred after protocol registration/finalization/approval.

Version date: Date of the protocol version change

Version number: Number or other identifier for the protocol version

Section of protocol: Brief text description of which sections of the protocol were amended

Amendment or update: Brief text description of what changes were involved with the protocol amendment

*Reason:* Brief text description of the rationale for the protocol amendment

| **Version date** | **Version number** | **Section of protocol** | **Amendment or update** | **Reason** |
| --- | --- | --- | --- | --- |
| <Text> | <Text> | <Text> | <Text> | <Text> |
| <Text> | <Text> | <Text> | <Text> | <Text> |
| <Text> | <Text> | <Text> | <Text> | <Text> |
| <Text> | <Text> | <Text> | <Text> | <Text> |

1. Milestones

Enter planned dates for study milestones.

*Milestone:* Brief text description of the milestone or deliverable

*Date:* Anticipated date of completion

#### Table 1 Milestones

| **Milestone** | **Date** |
| --- | --- |
| <Text> | <Text> |
| <Text> | <Text> |
| <Text> | <Text> |
| <Text> | <Text> |

1. Rationale and background

**What is known about the condition:** <Text>

**What is known about the exposure of interest:** <Text>

**Gaps in knowledge:** <Text>

**What is the expected contribution of this study?** <Text>

1. Research question and objectives

#### Table 2 Primary and secondary research questions and objective

The primary objective(s) defines the main aim of the study whereas the hypothesis refers to the main question being tested. This is usually what the study will be powered to detect. Secondary objectives are ancillary questions that may provide more information about the effects of treatment. Exposure refers to the therapeutic of interest whereas the comparator refers to an alternative option. The outcome refers to the clinical or other condition for which you are interested in understanding the effect of exposure. Time refers to when follow up begins and ends. Setting refers to the care settings that are relevant for the study (e.g. inpatient, ambulatory, emergency department). The main measure of effect refers to how you will estimate the effect of the exposure compared to the comparator on the outcome (e.g. hazard ratio, risk ratio, risk difference).

Duplicate table sections A and B as necessary for co-primary objectives or multiple secondary objectives.

1. **Primary research question and objective**

| **Objective:** | <Text> |
| --- | --- |
| **Hypothesis:** | <Text> |
| **Population *(mention key inclusion-exclusion criteria):*** | <Text> |
| **Exposure:** | <Text> |
| **Comparator:** | <Text> |
| **Outcome:** | <Text> |
| **Time *(when follow up begins and ends):*** | <Text> |
| **Setting:** | <Text> |
| **Main measure of effect:** | <Text> |

1. **Secondary research question 1 and objective**

| **Objective:** | <Text> |
| --- | --- |
| **Hypothesis:** | <Text> |
| **Population *(mention key inclusion-exclusion criteria):*** | <Text> |
| **Exposure:** | <Text> |
| **Comparator:** | <Text> |
| **Outcome:** | <Text> |
| **Time *(when follow up begins and ends):*** | <Text> |
| **Setting:** | <Text> |
| **Main measure of effect:** | <Text> |

1. Research methods
   1. Study design

**Research design (e.g. cohort, case-control, etc.):** <Text>

**Rationale for study design choice:** <Text>

- 1. Study design diagram

Create a design diagram to describe time 0 and relevant study windows used in the study design. A recommended framework is outlined in Schneeweiss et al. Graphical Depiction of Longitudinal Study Designs in Health Care Databases. Ann Intern Med. 2019; 170:398–406. The diagram following this framework can be created using power point templates or other software program of choice (www.repeatinitiative.org/projects). It is intended to be read from top to bottom, reflecting the order of operations to create an analytic cohort from a source longitudinal healthcare database. Temporality of assessment windows are clearly shown relative to the study entry (“index”) date, which is considered time 0. Bracketed number ranges denote the inclusive time windows for washout, inclusion/exclusion, and covariate assessment windows as well as follow up. Whether or not time 0 is included in an assessment window can also be visually distinguished by whether it overlaps the vertical arrow representing the primary anchor.

The diagram may include footnotes specifying the inclusion/exclusion criteria, covariates, and censoring criteria relevant to each assessment window.

Alternative design visualization approaches can be used as appropriate.

- 1. Setting

#### 7.3.1 Context and rationale for definition of time 0 (and other primary time anchors) for entry to the study population

The criteria that define the primary temporal anchor(s) that define how patients enter the study population are specified in this section. Other assessment windows used in the study design are defined relative to the primary temporal anchor(s). For example, assessment windows for inclusion-exclusion criteria or the follow up window. The primary temporal anchor is sometimes referred to as ‘time 0’, an ‘index date’ or ‘cohort entry date’.

<Text>

#### Table 3 Operational Definition of Time 0 (index date) and other primary time anchors

There should be (at least) one row for each study population. A key primary anchor is time 0, for when patients enter the study population and/or begin treatment, depending on the study. A primary time anchor is used as an anchor for defining assessment windows for inclusion-exclusion criteria, baseline characteristics, or follow up. If the study is descriptive, there may only be one primary temporal anchor for the study population. An active comparator study may have 2 rows, one for the exposure of interest and one for the comparator. Other, more complex study designs may have multiple primary temporal anchors. For example, a nested case-control study may have a primary anchor defined for the base cohort, and another for the case-defining event. A pregnancy study may have anchors defined by last menstrual period, trimester boundaries, and delivery date. See examples in Appendix 3.

The table below requests the following operational details:

*Study population name(s):* A brief text descriptor naming the study population that is identified for a primary or secondary analysis (e.g. exposed, comparator, pregnant women, patients with diabetes)

*Time anchor description:* A brief text description of the criterion used to define the time anchor, including at least one entry to define time 0, the point at which the patient enters the cohort (e.g. date of incident dispensation of Drug X, date of heart failure diagnosis, last menstrual period)

*Number of entries:* Indicate whether patients are allowed to enter the study population only once or multiple times (e.g. single entry, multiple entry)

*Type of entry*: Indicate whether the criterion for entry to the study population reflects an incident, prevalent, or other condition.

*Washout window*: If entry to the study population is defined as incident, use bracketed numbers representing time intervals anchored on a primary anchor (usually time 0) to specify the washout window. For example, [-180, -1] would reflect a washout window of 180 days prior to time 0, where the brackets indicate that the window is inclusive of the endpoints.

*Care setting:* Specify the care setting(s) that are used in the algorithm to define the time 0 (or other primary anchor) criterion. For example, IP = inpatient, ED = emergency department, etc.

*Code type*: Specify the type(s) of clinical codes that are used to define the time 0 (or other primary anchor) criterion. For example, ICD10 = International Classification of Diseases 10^th^ edition, NDC = National Drug Classification, etc.

*Diagnosis position:* If the algorithm to define the time 0 (or other primary anchor) criterion used inpatient codes, specify whether the algorithm restricts to primary diagnosis codes (indicating that the code is the primary reason for the encounter) or allows codes in secondary or any position (e.g. primary, secondary, any, n/a)

*Incident with respect to:* If the type of entry is defined as incident, provide a brief text description of what the patient is required to be incident to. For example, when identifying incident users of Drug X (oral formulation only), the investigator may wish to require that patients be incident with respect to Drug X (oral formulation) as well as Drug X (intravenous formulation) and Drug Y. This would be operationalized as having no record of exposure to any of these drugs during the specified washout period.

*Measurement characteristics/validation:* If there are measurement characteristics for the outcome algorithm (e.g. PPV, sensitivity, specificity) from publications, or from outcome validation within the study population (e.g., medical record review), provide this information.

*Source of algorithm:* Specify the source of algorithms to define the time 0 or primary anchor criteria.

| **Study population name(s)** | **Time Anchor Description**  **(e.g. time 0)** | **Number of entries** | **Type of entry** | **Washout window** | **Care Setting^1^** | **Code Type^2^** | **Diagnosis position** | **Incident with respect to…** | **Measurement characteristics/**  **validation** | **Source of algorithm** |
| --- | --- | --- | --- | --- | --- | --- | --- | --- | --- | --- |
|  |  |  |  |  |  |  |  |  |  |  |
|  |  |  |  |  |  |  |  |  |  |  |
|  |  |  |  |  |  |  |  |  |  |  |

^1^ IP = inpatient, OP = outpatient, ED = emergency department, OT = other, n/a = not applicable

^2^See appendix for listing of clinical codes for each study parameter

#### 7.3.2 Context and rationale for study inclusion criteria:

The study inclusion criteria are described conceptually, and the context or rationale for applying these criteria are described in this section. Note that defining “observable” patient time in the healthcare data source will almost always be relevant as an inclusion criterion. When using administrative claims data, this can be measured with dates of enrolment in insurance coverage, with or without bridging of short gaps in enrolment. When using electronic health record data, defining observable patient time may require making some strong assumptions. For example, assuming that patient encounters are always observable, that patients are observable between the first and last recorded encounter in the record, that patients are observable for X days before and after any recorded encounter, etc. Alternatively, one could specify inclusion based on algorithms to measure “loyalty” to a healthcare provider or EHR system.

<Text>

#### Table 4. Operational Definitions of Inclusion Criteria

The table below requests the following operational details:

*Criterion:* A brief text entry naming the inclusion criterion (e.g. baseline observable time, age, sex, atrial fibrillation)

*Details:* An optional brief text entry to provide more information about the inclusion criterion (e.g. age in years defined by (time 0 – year of birth)/365, baseline enrolment measured by Part A, B, D and no HMO insurance coverage with 30 day gaps allowed), 2 codes required

*Order of application:* Specify whether the inclusion criterion is applied before or after selection of the study entry date. For example, enter “before” if you will identify all possible study entry dates and apply the inclusion criterion to each possible entry date before choosing one or more study entry dates that has met the inclusion criterion. Enter “after” if you select the first possible study entry date and then apply the inclusion criterion. If the patient does not meet the inclusion criterion, then the patient will not be included. These decisions can impact which samples of person-time are included in the study.

*Assessment window*: Use bracketed numbers representing time intervals anchored on a primary anchor (usually time 0) to specify the window over which to evaluate patient data relevant for the inclusion criterion. For example, [-180, 0] would reflect an assessment window of 180 days prior to and including time 0, where the brackets indicate that the window is inclusive of the endpoints.

*Care setting:* Specify the care setting(s) that are used in the algorithm to define the inclusion criterion. For example, IP = inpatient, ED = emergency department, etc.

*Code type*: Specify the type(s) of clinical codes that are used to define the inclusion criterion. For example, ICD10 = International Classification of Diseases 10^th^ edition, NDC = National Drug Classification, etc.

*Diagnosis position:* If the algorithm to define the inclusion criterion used inpatient codes, specify whether the algorithm restricts to primary diagnosis codes (indicating that the code is the primary reason for the encounter) or allows codes in secondary or any position (e.g. primary, secondary, any, n/a)

*Applied to study populations:* Indicate which study populations the inclusion criterion should be applied to (study population names are specified in Table 2).

*Measurement characteristics/validation:* If there are measurement characteristics for the outcome algorithm (e.g. PPV, sensitivity, specificity) from publications, or from outcome validation within the study population (e.g., medical record review), provide this information.

*Source of algorithm:* Specify the source of algorithms to define the inclusion criteria.

| **Criterion** | **Details** | **Order of application** | **Assessment window** | **Care Settings¹** | **Code Type^2^** | **Diagnosis position^3^** | **Applied to study populations:** | **Measurement characteristics/**  **validation** | **Source for algorithm** |
| --- | --- | --- | --- | --- | --- | --- | --- | --- | --- |
|  |  |  |  |  |  |  |  |  |  |
|  |  |  |  |  |  |  |  |  |  |
|  |  |  |  |  |  |  |  |  |  |
|  |  |  |  |  |  |  |  |  |  |
|  |  |  |  |  |  |  |  |  |  |

^1^ IP = inpatient, OP = outpatient, ED = emergency department, OT = other, n/a = not applicable

^2^ See appendix for listing of clinical codes for each study parameter

^3^ Specify whether a diagnosis code is required to be in the primary position (main reason for encounter)

#### 7.3.3 Context and rationale for study exclusion criteria

The study inclusion criteria are described conceptually, and the context or rationale for applying these criteria are described in this section.

<Text>

#### Table 5. Operational Definitions of Exclusion Criteria

The table below requests the following operational details:

*Criterion:* A brief text entry naming the exclusion criterion (e.g. age, sex, atrial fibrillation)

*Details:* An optional brief text description to provide more information about the exclusion criterion (e.g. age in years defined by (time 0 – year of birth)/365, baseline enrolment measured by Part A, B, D and no HMO insurance coverage with 30 day gaps allowed), 2 codes required

*Order of application:* Specify whether the exclusion criterion is applied before or after selection of the study entry date. For example, enter “before” if you plan to apply the criterion, identify all possible study entry dates, and then choose one or more. Enter “after” if you select the first possible study entry date and then apply the exclusion criterion. If the patient does not meet the criterion, then the patient drops out. These decisions can impact which samples of person-time are included in the study.

*Assessment window*: Use bracketed numbers representing time intervals anchored on a primary anchor (usually time 0) to specify the window over which to evaluate patient data relevant for the exclusion criterion. For example, [-180, 0] would reflect an assessment window of 180 days prior to and including time 0, where the brackets indicate that the window is inclusive of the endpoints.

*Care setting:* Specify the care setting(s) that are used in the algorithm to define the exclusion criterion. For example, IP = inpatient, ED = emergency department, etc.

*Code type*: Specify the type(s) of clinical codes that are used to define the exclusion criterion. For example, ICD10 = International Classification of Diseases 10^th^ edition, NDC = National Drug Classification, etc.

*Diagnosis position:* If the algorithm to define the exclusion criterion used inpatient codes, specify whether the algorithm restricts to primary diagnosis codes (indicating that the code is the primary reason for the encounter) or allows codes in secondary or any position (e.g. primary, secondary, any, n/a)

*Applied to study populations:* Indicate which study populations the exclusion criterion should be applied to (study population names are specified in Table 2).

*Measurement characteristics/validation:* If there are measurement characteristics for the outcome algorithm (e.g. PPV, sensitivity, specificity) from publications, or from outcome validation within the study population (e.g., medical record review), provide this information.

*Source of algorithm:* Specify the source of algorithms to define the exclusion criteria.

| **Criterion** | **Details** | **Order of application** | **Assessment window** | **Care Settings¹** | **Code Type^2^** | **Diagnosis position^3^** | **Applied to study populations:** | **Measurement characteristics/**  **validation** | **Source for algorithm** |
| --- | --- | --- | --- | --- | --- | --- | --- | --- | --- |
|  |  |  |  |  |  |  |  |  |  |
|  |  |  |  |  |  |  |  |  |  |
|  |  |  |  |  |  |  |  |  |  |
|  |  |  |  |  |  |  |  |  |  |
|  |  |  |  |  |  |  |  |  |  |

^1^ IP = inpatient, OP = outpatient, ED = emergency department, OT = other, n/a = not applicable

^2^ See appendix for listing of clinical codes for each study parameter

^3^ Specify whether a diagnosis code is required to be in the primary position (main reason for encounter)

- 1. Variables

Context, rationale, and operation definition of exposures, outcomes, and other variables including measured risk factors, co-morbidities, co-medications, potential confounding variables and effect modifiers, etc. are specified in this section.

#### 7.4.1 Context and rationale for exposure(s) of interest

The study exposure (and comparator) are described conceptually, and the context or rationale for the choices are provided in this section.

<Text>

**Algorithm to define duration of exposure effect:**

If relevant, describe how duration of exposure will be defined operationally. Consider stockpiling algorithms, allowable gaps, and hypothesized duration of biological effect beyond the last available dose.

<Text>

#### Table 6. Operational Definitions of Exposure

Populate table if exposure is not already defined in Table 1 as a primary anchor criterion for defining time 0.

The table below requests the following operational details:

*Exposure group names:* A brief text entry naming the exposure groups (e.g. Drug X, Drug Y)

*Details:* An optional brief text description to provide more information about the exposure (e.g. oral formulation of Drug X)

*Assessment window*: Use bracketed numbers representing time intervals anchored on a primary anchor (usually time 0) to specify the window over which to evaluate patient data relevant for defining exposure status. For example, [-180, 0] would reflect an assessment window of 180 days prior to and including time 0, where the brackets indicate that the window is inclusive of the endpoints.

*Washout window*: If entry to the study population is defined as incident, use bracketed numbers representing time intervals anchored on a primary anchor (usually time 0) to specify the washout window. For example, [-180, -1] would reflect a washout window of 180 days prior to time 0, where the brackets indicate that the window is inclusive of the endpoints.

*Care setting:* Specify the care setting(s) that are used in the algorithm to define exposure. For example, IP = inpatient, ED = emergency department, etc.

*Code type*: Specify the type(s) of clinical codes that are used to define exposure. For example, ICD10 = International Classification of Diseases 10^th^ edition, NDC = National Drug Classification, etc.

*Diagnosis position:* If the algorithm to define exposure used inpatient codes, specify whether the algorithm restricts to primary diagnosis codes (indicating that the code is the primary reason for the encounter) or allows codes in secondary or any position (e.g. primary, secondary, any, n/a)

*Applied to study populations:* Indicate which study populations the exposure definition should be applied to (study population names are specified in Table 2).

*Incident with respect to:* If exposure is defined as incident, provide a brief text description of what the patient is required to be incident to. For example, when identifying incident users of Drug X (oral formulation only), the investigator may wish to require that patients be incident with respect to Drug X (oral formulation) as well as Drug X (intravenous formulation) and Drug Y. This would be operationalized as having no record of exposure to any of these drugs during the specified washout period.

*Measurement characteristics/validation:* If there are measurement characteristics for the outcome algorithm (e.g. PPV, sensitivity, specificity) from publications, or from outcome validation within the study population (e.g., medical record review), provide this information.

*Source of algorithm:* Specify the source of algorithms to define the exposure.

| **Exposure group name(s)** | **Details** | **Washout window** | **Assessment Window** | **Care Setting^1^** | **Code Type^2^** | **Diagnosis position^3^** | **Applied to study populations:** | **Incident with respect to…** | **Measurement characteristics/ validation** | **Source of algorithm** |
| --- | --- | --- | --- | --- | --- | --- | --- | --- | --- | --- |
|  |  |  |  |  |  |  |  |  |  |  |
|  |  |  |  |  |  |  |  |  |  |  |
|  |  |  |  |  |  |  |  |  |  |  |

^1^ IP = inpatient, OP = outpatient, ED = emergency department, OT = other, n/a = not applicable

^2^ See appendix for listing of clinical codes for each study parameter

^3^ Specify whether a diagnosis code is required to be in the primary position (main reason for encounter)

#### 7.4.2 Context and rationale for outcome(s) of interest

The study outcome(s) are described conceptually, and the context or rationale for the choices are provided in this section.

<Text>

#### Table 7. Operational Definitions of Outcome

The table below requests the following operational details:

*Outcome name:* A brief text entry naming the outcome (e.g. myocardial infarction, death)

*Details:* An optional brief text description to provide more information about the outcome (e.g. 2^nd^ of 2 codes within 7 days)

*Primary outcome?:* Indicate whether the outcome is a primary or secondary outcome (e.g. Yes, if primary; no, if secondary)

*Type of outcome:* Specify the variable type for the outcome (e.g. time-to-event, binary, count, continuous)

*Washout window*: If the outcome is required to be incident, use bracketed numbers representing time intervals anchored on a primary anchor (usually time 0) to specify the washout window. For example, [-180, -1] would reflect a washout window of 180 days prior to time 0, where the brackets indicate that the window is inclusive of the endpoints.

*Care setting:* Specify the care setting(s) that are used in the algorithm to define the outcome. For example, IP = inpatient, ED = emergency department, etc.

*Code type*: Specify the type(s) of clinical codes that are used to define the outcome. For example, ICD10 = International Classification of Diseases 10^th^ edition, NDC = National Drug Classification, etc.

*Diagnosis position:* If the algorithm to define the outcome used inpatient codes, specify whether the algorithm restricts to primary diagnosis codes (indicating that the code is the primary reason for the encounter) or allows codes in secondary or any position (e.g. primary, secondary, any, n/a)

*Applied to study populations:* Indicate which study populations the outcome should be evaluated in (study population names are specified in Table 2).

*Measurement characteristics/validation:* If there are measurement characteristics for the outcome algorithm (e.g. PPV, sensitivity, specificity) from publications, or from outcome validation within the study population (e.g., medical record review), provide this information.

*Source of algorithm:* Specify the source of algorithms to define the exclusion criteria.

| **Outcome name** | **Details** | **Primary outcome?** | **Type of outcome** | **Washout window** | **Care Settings¹** | **Code Type^2^** | **Diagnosis Position^3^** | **Applied to study populations:** | **Measurement characteristics/**  **validation** | **Source of algorithm** |
| --- | --- | --- | --- | --- | --- | --- | --- | --- | --- | --- |
|  |  |  |  |  |  |  |  |  |  |  |
|  |  |  |  |  |  |  |  |  |  |  |

^1^ IP = inpatient, OP = outpatient, ED = emergency department, OT = other, n/a = not applicable

^2^ See appendix for listing of clinical codes for each study parameter

^3^ Specify whether a diagnosis code is required to be in the primary position (main reason for encounter)

#### 7.4.3 Context and rationale for follow up

The study follow up is described conceptually, and the context or rationale for the choices are provided in this section.

<Text>

#### Table 8. Operational Definitions of Follow Up

The table below requests the following operational details:

*Follow up start:* Specify when follow up begins relative to time 0 (e.g. day 1). Consider whether an induction/latency window is relevant for the hypothesized onset of the effect of exposure on the outcome (operationally, this would mean delaying the start of follow up (e.g. follow up start on day 30).

*Follow up end:* Indicate ‘Yes’ or ‘No’ for each of the options. Specify additional details as relevant.

(day 0) and select each criterion that ends follow up.

|  |  |  |  |
| --- | --- | --- | --- |
| **Follow up start** | <Text> |  |  |
| **Follow up end^1^** | **Select all that apply** |  | **Specify** |
| **Date of outcome** | <Text> |  | <Text> |
| **Date of death** | <Text> |  | <Text> |
| **End of observation in data** | <Text> |  | <Text> |
| **Day X following index date**  *(specify day)* | <Text> |  | <Text> |
| **End of study period**  (specify date) | <Text> |  | <Text> |
| **End of exposure**  *(specify operational details,*  *e.g. stockpiling algorithm, grace period)* | <Text> |  | <Text> |
| **Date of add to/switch from exposure**  *(specify algorithm)* | <Text> |  | <Text> |
| **Other date** *(specify)* | <Text> |  | <Text> |

^1^ Follow up ends at the first occurrence of any of the selected criteria that end follow up.

#### 7.4.4 Context and rationale for covariates (confounding variables and effect modifiers, e.g. risk factors, comorbidities, comedications)

The study covariates are described conceptually, and the context or rationale for the choices are provided in this section. Description of data driven methods to select covariates through machine learning algorithms can be described here, with detail on operational choices including parameter settings for the macro/function that is used.

<Text>

#### Table 9. Operational Definitions of Covariates

The table below requests the following operational details:

*Characteristic:* A brief text entry naming the characteristic (e.g. age, sex, atrial fibrillation)

*Details:* An optional brief text description to provide more information about the covariate (e.g. age in years defined by (time 0 – year of birth)/365)

*Type of variable:* Specify the variable type for the covariate (e.g. binary, count, continuous)

*Assessment window*: Use bracketed numbers representing time intervals anchored on a primary anchor (usually time 0) to specify the window over which to evaluate patient data relevant for the covariate. For example, [-180, 0] would reflect an assessment window of 180 days prior to and including time 0, where the brackets indicate that the window is inclusive of the endpoints.

*Care setting:* Specify the care setting(s) that are used in the algorithm to define the covariate. For example, IP = inpatient, ED = emergency department, etc.

*Code type*: Specify the type(s) of clinical codes that are used to define the covariate. For example, ICD10 = International Classification of Diseases 10^th^ edition, NDC = National Drug Classification, etc.

*Diagnosis position:* If the algorithm to define the covariate used inpatient codes, specify whether the algorithm restricts to primary diagnosis codes (indicating that the code is the primary reason for the encounter) or allows codes in secondary or any position (e.g. primary, secondary, any, n/a)

*Applied to study populations:* Indicate which study populations the covariate should be measured in (study population names are specified in Table 2).

*Measurement characteristics/validation:* If there are measurement characteristics for the outcome algorithm (e.g. PPV, sensitivity, specificity) from publications, or from outcome validation within the study population (e.g., medical record review), provide this information.

*Source of algorithm:* Specify the source of algorithms to define the covariates.

| **Characteristic** | **Details** | **Type of variable** | **Assessment window** | **Care Settings¹** | **Code Type^2^** | **Diagnosis Position^3^** | **Applied to study populations:** | **Measurement characteristics/**  **validation** | **Source for algorithm** |
| --- | --- | --- | --- | --- | --- | --- | --- | --- | --- |
|  |  |  |  |  |  |  |  |  |  |
|  |  |  |  |  |  |  |  |  |  |

^1^ IP = inpatient, OP = outpatient, ED = emergency department, OT = other, n/a = not applicable

^2^ See appendix for listing of clinical codes for each study parameter

^3^ Specify whether a diagnosis code is required to be in the primary position (main reason for encounter)

- 1. Data analysis

#### 7.5.1 Context and rationale for analysis plan

The inferential data analysis plan is described conceptually, and the context or rationale for the choices are provided in this section.

<Text>

#### Table 10. Primary, secondary, and subgroup analysis specification

The table below requests the following operational details for each sub-table that reflect the primary and secondary analyses:

*Hypothesis:* State the hypothesis being tested

*Exposure contrast:* State the exposure contrast being evaluated

*Outcome:* State the outcome being evaluated

*Analytic software:* Name the software being used (include packages, version numbers, etc.)

*Model(s):* Provide details about the models involved in inferential analyses (e.g. Cox proportional hazards outcome model: followuptime*status(0) = exposure; Logistic regression propensity score model: exposure = COV1 + COV2)

*Confounding adjustment method:* Check the relevant methods and provide relevant details as prompted in the table.

*Missing data methods:* Check the relevant methods and provide relevant details as prompted in the table.

*Subgroup analysis:* List subgroup analyses that will be conducted based on the primary analysis parameters

1. **Primary analysis**

| **Hypothesis:** | <Text> |
| --- | --- |
| **Exposure contrast:** | <Text> |
| **Outcome:** | <Text> |
| **Analytic software:** | <Text> |
| **Model(s):**  ***(provide details or code)*** | <Text> |
| **Confounding adjustment method** | ***Name method and provide relevant details, e.g. bivariate, multivariable, propensity score matching (specify matching algorithm ratio and caliper), propensity score weighting (specify weight formula, trimming, truncation), propensity score stratification (specify strata definition), other.*** |
|  | <Text> |
| **Missing data methods** | ***Name method and provide relevant details, e.g. missing indicators, complete case, last value carried forward, multiple imputation (specify model/variables), other.*** |
|  | <Text> |
| **Subgroup Analyses** | ***List all subgroups*** |
|  | <Text> |

1. **Secondary Analysis 1**

| **Hypothesis:** | <Text> |
| --- | --- |
| **Exposure contrast:** | <Text> |
| **Outcome:** | <Text> |
| **Analytic software:** | <Text> |
| **Model(s):**  ***(provide details or code)*** | <Text> |
| **Confounding adjustment method** | ***Name method and provide relevant details, e.g. bivariate, multivariable, propensity score matching (specify matching algorithm ratio and caliper), propensity score weighting (specify weight formula, trimming, truncation), propensity score stratification (specify strata definition), other.*** |
|  | <Text> |
| **Missing data methods** | ***Name method and provide relevant details, e.g. missing indicators, complete case, last value carried forward, multiple imputation (specify model/variables), other.*** |
|  | <Text> |
| **Subgroup Analyses** | ***List all subgroups*** |
|  | <Text> |

#### Table 11. Sensitivity analyses – rationale, strengths and limitations

The table below is used to detail the sensitivity analyses, the rationale for conducting them (in other words, stating what the investigator intends to learn from doing the sensitivity analysis), and any strengths or limitations of the sensitivity analysis relative to the primary analysis.

|  | **What is being varied? How?** | **Why?  (What do you expect to learn?)** | **Strengths of the sensitivity analysis compared to the primary** | **Limitations of the sensitivity analysis compared to the primary** |
| --- | --- | --- | --- | --- |
| <Text> | <Text> | <Text> | <Text> | <Text> |
| <Text> | <Text> | <Text> | <Text> | <Text> |

- 1. Data sources

This section includes structured prompts to state the reasons for selecting the data, strengths and limitations of the data source(s) with respect to the study objective and capturing inclusion-exclusion criteria, exposure, outcome, covariates, and follow up time. Include context about the potential impact of missing data and information about data source/provenance/curation. This may include a detailed evaluation of the fitness-for-purpose of data source options.

#### 7.6.1 Context and rationale for data sources

**Reason for selection:** <Text>

**Strengths of data source(s):** <Text>

**Limitations of data source(s):** <Text>

**Data source provenance/curation:** <Text>

#### Table 12. Metadata about data sources and software

The table below requests the following meta-data details:

*Data source(s):* Provide the name of the data source

*Study period:* Provide the calendar time boundaries for data used to create the analysed study dataset, including exposures, inclusion and exclusion criteria, covariates, outcome, and follow-up.

*Eligible cohort entry period:* Provide the calendar time boundaries for during which patients could enter the study population.

*Data version (or date of last update):* Provide information on the version of the source data. This may be a version number, date of last extract-transform-load (ETL) or other meta-data to identify which version of the data were (or will be) used for the research.

*Data linkage*: Indicate whether the data will be linked to another data source (and if so, how and provide linkage performance characteristics if available). Details can be provided in an appendix that is referenced in the table.

*Conversion to common data model:* Indicate whether the data is converted to a common data model and which version (e.g. Sentinel CDM v 8.0.0, OMOP CDM v 6.0).

*Software for data management:* Name software that is used to manage or maintain the data.

|  | **Data 1** | **Data 2** | **Data 3** |  |
| --- | --- | --- | --- | --- |
| **Data Source(s):** | <Text> | <Text> | <Text> | |
| **Study Period:** | <Text> | <Text> | <Text> | |
| **Eligible Cohort Entry Period:** | <Text> | <Text> | <Text> | |
| **Data Version (or date of last update):** | <Text> | <Text> | <Text> | |
| **Data sampling/extraction criteria:** | <Text> | <Text> | <Text> | |
| **Type(s) of data:** | <Text> | <Text> | <Text> | |
| **Data linkage:** | <Text> | <Text> | <Text> | |
| **Conversion to CDM*:** | <Text> | <Text> | <Text> | |
| **Software for data management:** | <Text> | <Text> | <Text> | |

*CDM = Common Data Model

- 1. Data management

Describe the data management procedures used in the study, including procedures, policies, and infrastructure for data storage, transfer, back up, and information security.

<Text>

- 1. Quality control

Describe steps to ensure data quality including quality assurance and quality check procedures, double programming, source data verification, validation of endpoints, data transformation and linkages, assessment of the reliability of the data (e.g. missing or miscoded data, lags in data capture).

<Text>

- 1. Study size and feasibility

Provide projected study size, precision sought for study estimates or calculation of the sample size that can minimally detect a pre-specified risk with a pre-specified statistical precision. All assumptions used to calculate the study size or precision of the study should be presented and justified (including whether feasibility counts were generated).

<Text>

#### Table 13. Power and sample size

< Provide table with relevant information >

1. Limitation of the methods

Discuss potential limitations of the study design, and analytic methods, including issues relating to confounding, bias, generalisability, and random error. Discuss the steps that will be taken to reduce the potential impact of these limitations, e.g. in terms of design or sensitivity analyses.

<Text>

1. Protection of human subjects

If relevant, discuss privacy safeguards, ethics or human subjects review board processes taken to comply with requirements for minimizing the risks and ensuring the privacy of patients whose data is used for non-interventional studies involving secondary use of real-world data. If the study is considered exempt by the relevant ethics board this should be stated with the reason it is considered exempt.

<Text>

1. Reporting of adverse events

If relevant, describe procedures for the collection, management and reporting of individual cases of adverse events/adverse reactions (see GVP Module VI) and any new information that might influence the evaluation of the benefit-risk balance of the product while the study is being conducted. Arrangements made between marketing authorization holders for the management and reporting of adverse events/reactions in joint post-authorization safety studies should be specified. For studies where reporting is not required, this should be stated.

The European Medicines Agency has published guidance on when this section is required: *Guideline on good pharmacovigilance practices (GVP) Module VI – Collection, management and submission of reports of suspected adverse reactions to medicinal products (Rev 2)*. 2017. Accessed 11/18/2021. <https://www.ema.europa.eu/en/human-regulatory/post-authorisation/pharmacovigilance/good-pharmacovigilance-practices>

“*The requirements provided in this Module do not apply to non-interventional post-authorisation studies conducted by organisations such as academia, medical research charities or research organisations in the public sector. These organisations should follow the local requirements as regards the submission of cases of suspected adverse reactions to the competent authority in the Member State where the reaction occurred. However, where a study conducted by one of these organisations is directly initiated, managed, or financed by a marketing authorisation holder, or where its design is controlled by a marketing authorisation holder (voluntarily or pursuant to obligations imposed in accordance with Article 21a and 22a of Directive 2001/83/EC, or Article 9(4) and 10a of Regulation (EC) No 726/2004), the requirements provided in this Module are applicable.”*

<Text>

1. References

Provide a numbered list of references for any works cited in the protocol. Sufficient information must be provided to retrieve the cited work.

<Numbered list >

1. Appendices

Provide a list of appendices that are provided as separate documents. The expectation is that machine readable appendices that list out the actual clinical code algorithms used to define study parameters outlined in the protocol tables will be provided. Other appendices may be included to provide more detail on other things such as decisions made when converting source data to a common data model or details on data linkage and performance.

< List of appendices >
